# Longitudinal multiplexity and structural constraints of online emergency collaborative networks: A tale of two Chinese societies

**DOI:** 10.1371/journal.pone.0289277

**Published:** 2023-07-27

**Authors:** Xiao Wang

**Affiliations:** School of Journalism and Communication, Nanjing University, Nanjing, Jiangsu, China; Shandong University of Science and Technology, CHINA

## Abstract

Drawing upon the concept of longitudinal multiplexity and the Institutional Collection Action (ICA) framework, this article conducts a longitudinal observation of online emergency collaborative networks (ECNs) built and sustained among organizational actors within Shenzhen and Hong Kong in response to Typhoon Mangkhut. In addition to investigating the multiplex relationships among three types of online ECNs (i.e., preparedness networks, response networks, recovery networks), this article takes a comparative approach to examining how the structural constraints imposed by distinct emergency management systems (EMSs) influence the network formation and evolution as the disaster evolves over time. Findings obtained from a series of inferential network analyses reveal that preexisting online collaborative ties formed at the disaster preparedness stage are important for increasing organizations’ tendency to build and sustain online collaborations during disaster response and recovery. Moreover, the decentralized EMS in Hong Kong exhibits more effectiveness in facilitating online network changes during both transitional periods. These findings demonstrate a pressing need for emergency researchers and frontline communication managers to understand the dynamic relationships among online ECNs across different disaster phases and further explore potential opportunities to facilitate online emergency collaboration on a broader scale.

## Introduction

In addressing natural or man-made disasters, organizational actors work together to achieve collective goals that cannot be reached by independent efforts. Given this form of behavioral interdependency, that is, actions and decision-making taken by one actor influence those of others, emergency collaborative networks (ECNs) prove to be of critical importance to cope with emergencies across organizational, sectoral, and jurisdictional boundaries. Over the past decade, social media sites and applications (e.g., Facebook, Twitter) represent prevailing information and communication technologies (ICTs) that expedite timely and transparent information sharing among ECN actors [1–5]. Emergency managers have made extensive use of these applications to meet their common needs to quickly seek and share messages (e.g., disaster warnings, on-site news updates, preparedness guidance, personnel recruitment, opportunities for drills/training programs/joint exercises).

Also, the rapid development of social media applications provides a new venue for navigating sources of interorganizational relationships in emergency collaboration and analyzing such network data in empirical investigations [6–8]. These network data have enabled the access to not only relevant attributes of organizational users (e.g., names, types, history), but also social media metrics and interactions through which researchers are able to analyze the information sharing and resource mobilization among network actors in disastrous situations. Methodologically, using social network data renders the opportunity to move beyond the egocentric self-reports that could bias network measures [9] and to track the formation and development of ECNs at different stages of emergency management.

Intergovernmental and cross-sectoral collaboration during emergencies encompasses not only the mobilization of resources, personnel, knowledge, and information, but also the development of multilevel interorganizational relationships [10]. The ECN is a social structure of strong embeddedness [11], and its performance in addressing disastrous situations is largely a result of organizational actors’ multiple types of connections and interactions with one another before, during, and after disasters [12, 13]. Although most existing research captures only a snapshot of ECN without any consideration of temporality, an emerging body of empirical studies recognizes the multiplex relationships between organizations during disaster preparedness, response, and recovery (e.g., [10, 14, 15]). Yet so far, limited attention has been paid to investigating the dynamic formation and evolution of ECNs, let alone those being conducted against the social media environment.

Furthermore, although the geographic scope of research on emergency collaboration has recently been greatly expanded to countries other than the U.S. (e.g., [15–17]), direct comparison of network formation and evolution is largely bounded by the difficulty of harmonizing multi-country or multi-region comparative research projects on the same topic utilizing similar research designs. In practice, ECNs reflect how a broadly defined social system responds to natural or man-made disasters in a given socio-political context. It is thus expected that the formation and evolution of collaborative ties and networks are inevitably influenced by structural constraints unfolded in emergency management systems (EMSs). Adopting EMS as a context-specific factor to examine its conditional effects on the development and evolution of ECNs represents an effective response to the recent call for more comparative studies on emergency collaboration [18].

Drawing upon the concept of longitudinal multiplexity and the Institutional Collective Action (ICA) framework developed by Feiock and his colleagues (e.g., [19–21]), this article employs the case of Typhoon Mangkhut and conducts a longitudinal observation of online ECNs built and sustained among organizational actors within Shenzhen and Hong Kong, two adjacent Chinese metropolitan areas with similar vulnerability to natural disasters while practicing different EMSs. In addition to examining the relationship among three types of online ECNs (i.e., preparedness networks, response networks, recovery networks), this study also affords a comparative approach to explore how the structural constraints imposed by EMSs influence the network formation and evolution as the disaster evolves over time.

## Conceptualization

### Longitudinal multiplexity of online ECNs

*Multiplexity* denotes the extent to which organizations are committed to diverse collaborative activities [22] as well as the strength of ties between them [23]. The emergency management cycle, one of FEMA’s most frequently used frameworks, consists of four phases: mitigation, preparedness, response, and recovery. Specifically, mitigation and preparedness occur before the disaster ever strikes. In these two phases, organizations prepare for potential disasters and hazards through formulating emergency plans, raising public awareness, and enhancing infrastructure quality. During the response phase, organizations provide emergency assistance, maintain transportation and communication, and operate other support functions. Immediately after the disaster, organizational actors enter the long-term recovery by restoring public services to full or sufficient functional capabilities and rebuilding infrastructures and institutions damaged in the devastation. In practice, effective disaster responses typically rely on the synergy of multiple ECNs generated and sustained across different phases of emergency management, while the coupling failure of various relational types is likely to compound ECNs’ overall vulnerability.

As the disaster evolves, participating actors’ engagement in the social media environment may exhibit diverse communication patterns, and collaborative network structures constructed online are also likely to change in accordance with how actors leverage various social media features to achieve their collective goals [24]. Messages sent during emergency response, for instance, incorporate more representations of information broadcasting and brokerage (e.g., retweets) while fewer displays of interactive activities (e.g., replies) when compared to ordinary Twitter use [4]. The engagement of organizational accounts may reveal a different communication pattern. A content analysis of the organizational Twitter use shortly after the Haiti earthquake in 2010 revealed a decreased use of information dissemination strategies (e.g., links) and a consistent use of techniques to involve other organizations (e.g., retweets, mentions) [25]. In the context of China, social organizations such as NPOs and NGOs were found to be more likely to adopt WeChat groups as a major platform for disseminating and sharing disaster information, whereas government agencies and private enterprises used WeChat generally as a supplementary communication tool [26]. This illustrates the fact that as the emergency unfolds over time, multiple ECNs built upon diverse technological features of social media may present distinct structural patterns. Hence, this study scrutinizes three types of online ECNs that can be identified in line with the emergency management cycle: online preparedness networks, online response networks, and online recovery networks.

Within the online preparedness network, organizational actors keep regular and informal online interactions with other public, private, and non-profit organizations staying active in or beyond the domain of emergency management. This type of network involves scant formal collaborative activities and programs designed to cope with disastrous situations. To take advantage of unexploited personnel, expertise, resources, and information, organizations need to better grasp the informal network structures in order to align them with organizational goals and to comprehend the underlying logic of informal networks that have not been acknowledged in any form of cooperative agreement [27]. Moreover, informal networks formed during this phase may mimic friendship networks in rendering venues for organizations to channel resources, share information, and strengthen capacity [10, 28]. Such informal networks are likely to formalize over the long haul and further facilitate the information sharing and resource mobilization when the disaster really strikes [29].

Previous research on *structural embeddedness* demonstrates how actors’ network positions in terms of preexisting or shared third-party ties contribute to the formation of new ties and economic cooperation [30–32]. Embedded in a densely multiplex network, organizations seek a variety of means to access fit links [33], obtain relational benefits [34], and minimize the uncertainty of collaborative activities [35]. They thus tend to sustain collaborative relationships with other organizations such that all participating actors present interdependency in sharing resources and information [36]. Similarly, empirical efforts on collective action networks (e.g., social movement coalition) suggest that the preestablished connections between organizational members help promote the communication, trust, and common values, which in turn encourage collaborative behaviors in collective actions [37].

In the context of emergency management, researchers have also stressed the importance of preexisting relationships in intergovernmental and cross-sectoral collaboration and coordination [38]. Confronted with disasters, organizations come to the rescue of afflicted areas and communities, frequently resorting to preexisting ties for prompt emergency planning and coordination. These ties are often built and developed in the preparedness phase, when a broad spectrum of organizations have been mobilized to take action to support and enhance the emergency response (e.g., trainings, drills, joint exercises). An examination of the partnership between public and non-profit organizations revealed that pre-disaster ties would enable affected communities to perform better, owing to the fact that mutual trust and commitment can be built and enhanced among network actors in their daily working relationships [39]. This study argues that the importance of structural embeddedness remains constant online such that the symbolic forms of interorganizational relationships rely on organizations’ actual connections in the real world [40]. For example, the hyperlink network constructed by Chinese environmental organizations reveals high density, which moves beyond the general features of Internet-based technologies yet reflecting the close offline connections between organizational actors in the domain of environmental protection [41]. Therefore, the first set of hypotheses is proposed as follows:

**Hypothesis 1 (H1)**: Organizational actors’ online preparedness networks positively correlate with their online response networks.
**Hypothesis 2 (H2)**: Organizational actors’ online preparedness networks positively correlate with their online recovery networks.

Unlike informal preparedness networks, online networks built in the response and recovery phases usually incorporate more formal collaboration and coordination. During actual disasters, interorganizational ties constructed through various social media features may enable organizations to share common goals and values on account of varying levels of participation in the collaborative network. Network ties formed at the preparedness stage have also been enhanced and developed because actors prefer to seek new partners to address emergent needs and achieve immediate goals [42]. Therefore, online preparedness ties are not necessarily transformed into effective working relationships in response to disasters since preexisting connections may not always be exploited in line with the resource interdependency shaped in disastrous scenarios.

Nonetheless, the collaborative approach to sustainable recovery after disasters indicates that the post-disaster development of impacted communities and areas is a rather complex and non-linear social process driven by both political-administrative arrangements [43] and multiple accessible resources [44]. The involvement of various organizations and sectors forms an intricate post-disaster ECN maintained by common interests and shared resources, in which external aid and assistance is embedded. As a result, the structural inertia of online ECNs may persist even after external assistance withdraws from the affected areas in a geographic sense. In other words, organizations’ needs for resources and information at the post-disaster recovery stage can be better accommodated through response networks. Therefore, the following hypothesis is posited to investigate the correlation between online ECNs built during disaster response and those sustained in the post-disaster recovery:

**Hypothesis 3 (H3)**: Organizational actors’ online response networks positively correlate with their online recovery networks.

As discussed earlier, the sustainability of external aid and assistance poses the principal challenge for achieving a sustainable recovery; that is, the doubt remains about whether the long-term disaster recovery of impacted areas and communities can be sustained once the outside help subsides. Prior research accentuates the combination of local reliance on internal capacity and flexible management of external assistance in addressing post-disaster needs (e.g., [45–47]). A systematic review of the disaster assistance framework in the U.S. concluded that post-disaster aid and assistance should rely on response operations with numerous accessible resources rather than on plans and arrangements stipulated prior to the disaster [44]. It also questioned the current U.S. disaster recovery policy because involved organizations have conflicting assistance plans on the process before the focal event, thus lacking sufficient collaboration and coordination among public, private, and non-profit sectors. Following this stream of research, this study contends that online collaborative ties built in response operations are more likely to contribute to facilitating sustained interorganizational interactions during post-disaster recovery because the structural inertia may come into existence between two types of formal connections, and more importantly, further reinforce as collaborative efforts during response and recovery are both made to address actual disastrous situations. Hence, a hypothesis comparing correlations among three types of online ECNs is proposed as follows:

**Hypothesis 4 (H4)**: The correlations between online response networks and online recovery networks are higher than the correlations between online preparedness networks and online response networks and between online preparedness networks and online recovery networks.

### Structural constraints on network formation and evolution

In a disaster setting, each EMS entails a certain level of authority in constraining the autonomy of organizational actors and tackles collaboration dilemmas differently. For example, actors working under a coercive EMS tend to forgo more localized autonomy than those operating under a more voluntary and self-organized system [48]. Although actual response networks may differ substantially from planned networks institutionalized in political-administrative arrangements, this gap can be more salient in a centralized and coercive system than in a decentralized and voluntary system [26].

In general, there are three types of EMSs: centralized/vertical, decentralized/horizontal, and a hybrid of both [10, 49]. In the U.S., there are two major systems operating across different levels of the federal structure: the Incident Command System (ICS) and the Emergency Support Function (ESF) System. The ICS-based approach represents a centralized and command-and-control coordination mechanism, which highlights the hierarchy of authority and standard operations for managing emergencies. When a disaster occurs, the system allows the federal government to exert direct control over local emergency management actions [50, 51]. State and local governments are required to conform to the unified command framework in managing resources, assigning tasks, and making decisions. Despite its capacity to swiftly issue orders and return real-time situations [52], the ICS has long been criticized for its lack of flexibility in managing interjurisdictional and cross-sectoral collaborations as well as the utilization of community resources [53]. The delayed rescue efforts in conjunction with the collapse of communication and coordination among the U.S. government agencies after Hurricane Katrina have manifested the inadequacies of such a centralized coordination mechanism [54]. In contrast to the ICS, the ESF-based approach embodied in the National Response Framework (NRF) represents a decentralized coordination mechanism emphasizing the crucial role of state and local government agencies, as well as private and nonprofit sectors. Within the ESF-based system, organizational actors operate according to 15 ESFs, which necessitate a more horizontal and collaborative approach to an enhanced coordination across public, private, and nonprofit sectors [51, 55].

According to the ICA framework, coordination mechanisms are most effective when they overcome collective action problems while incurring the lowest transaction costs [20, 21, 56]. By and large, the centralized EMS operates a typical command-and-control approach, which categorizes response organizations and emergency tasks into ordered levels and forms a hierarchical power structure to ensure the efficiency of workflow at all levels [53, 57]. Although this system incurs high autonomy costs on actors, it can mitigate the uncertainty of disastrous situations and the associated *defection risks* (i.e., the probability of commitment violation; [15]). Furthermore, the coercive nature of the centralized system may reinforce the enforcement mechanism for mutual trust and commitments, thus limiting the possibility of actors engaging in opportunistic behavior. As thus, the centralized EMS can be more effective in facilitating network changes during the transition from disaster preparedness to response, which is characterized by an abrupt increase of complexity and uncertainty.

Compared with the centralized approach to emergency collaboration, the decentralized structure demonstrates more flexibility and resilience because it eases interactions between organizations across different sectors and jurisdictions, allowing actors to reach out to organizations of partnership and adapt to one another in alignment with agreed goals and shared values [57]. In addition to the low autonomy costs incurred on actors, the decentralized system can mitigate resource insufficiency and the associated *coordination risks* (i.e., probability of inaction; [21]) by securing access to nonoverlapping resources and information and helping actors identify mutually beneficial situations. Hence, the decentralized EMS appears more effective to facilitate network changes during the transition from disaster response to recovery, which is characterized by resource scarcity and interdependency. Yet so far, there have been few comparative studies on network development and evolution across different EMSs, not to mention those focusing on structural constraints on the social media landscape. The following set of hypotheses is thus proposed:

**Hypothesis 5 (H5)**: Online preparedness networks within the centralized EMS have stronger predictive power in the formation of online response networks than do their counterparts within the decentralized EMS.
**Hypothesis 6 (H6)**: Online response networks within the decentralized EMS have stronger predictive power in the formation of online recovery networks than do their counterparts within the centralized EMS.

### Attribute-based network homophily

Prior interorganizational network research suggests a salient homophily effect that actors sharing similar attributes are more likely to build and sustain connections with each other [58]. Due to differences in organizational attributes, the internal structure, mobilizing capacity, and designated roles and responsibilities can vary greatly across organizations and sectors [39]. Hence, the amount and type of accessible information together with rescue resources are likely to cluster actors throughout different emergency phases. From the ICA perspective, organizational attributes can influence how actors perceive collaboration risks [48]. Collaboration and coordination among similar actors can reduce their perceived risks while raising their estimation of net expected benefit (i.e., the difference between expected benefit and expected cost) because previously shared behavioral norms and authority may reinforce interorganizational trust and operational relationships in response to a disaster [52]. The openness and transparency of online communication activities make it easier for organizations to locate those with similar interests and more difficult to conceal their preferences, thereby strengthening the homophily effect [59].

This study has identified organization type, geographic scale, and geo-location as three organizational attributes with the potential to contribute to network homophily. In response to major disasters, organizational actors of the same type share common goals and network constraints, which are usually considered as prerequisites for interorganizational alliance [37, 60]. For instance, governments at all levels play a leading role in the institutionalized emergency management. On account of the frequent flow of political-administrative arrangements and commands, government agencies are more likely to build connections with one another, rather than contacting other types of participating organizations. Similar geo-locations of two organizations indicate that they may face common external environment and similar vulnerability to natural disasters, which prompt them to form interlocal collaborative ties to exchange disaster experiences and lessons in response to floods or hurricanes [15, 17, 61]. Conversely, a greater geographic distance may increase the cost of communication and coordination, thus impeding the creation and development of network relationships between actors. Moreover, network links between organizational actors with the same geographic scale are considered fit because they share similar authorities, commands, identities, and legitimacy. Nevertheless, technological features of social media may afford a different communication pattern that transcends geographic boundaries through more accessible connections and relationship management [62]. Also, it remains unclear whether the homophily effect of geographic scale applies because collaboration in disastrous situations is largely fortified by intergovernmental and cross-sectoral partnership. Due to the inconclusive evidence, an exploratory research question is proposed as follows:

#### Research Question 1 (RQ1)

How does the attribute-based homophily effect in terms of organization type, geo-location, and geographic scale apply to the formation and evolution of online ECNs?

## Context of the study: The case of Typhoon Mangkhut

To examine the hypotheses and research questions proposed above, this study focuses on online ECNs within Shenzhen and Hong Kong, two regions practicing different EMSs. Both situated in the Pearl River Delta (PRD) which is one of the world’s most densely populated and disaster-prone metropolitan areas [63], Shenzhen demonstrates a more centralized EMS, whereas Hong Kong presents a relatively decentralized network structure for emergency responses.

On September 14, 2018, Super Typhoon Mangkhut made landfall in the northeast of Luzon Island as a Category-5 super typhoon on the U.S. scale and subsequently impacted southern China. It then became the most powerful tropical cyclone in the Philippines since the record-breaking Typhoon Haiyan in 2013 and the most intense storm to batter South China since records began in 1946. On September 15, the intensity of Mangkhut called for the highest warning signal No.10 remaining in Hong Kong and the highest level of alerts (i.e., red alerts) issued by the Meteorological Bureau of Shenzhen. Also, the extensive devastation resulted in comparable social disturbance and economic losses to both regions. Based on disaster modeling analytics, the damage bill inflicted on Hong Kong and mainland China could amount to US$50 billion, making Mangkhut the costliest destruction in the history of PRD [64]. While Mangkhut is a natural disaster of less severity compared to Haiyan, if offers emergency researchers and practitioners a rare opportunity to scrutinize structural constraints on the formation and evolution of online ECNs, and more importantly, to reflect how these effects can induce more context-sensitive understandings about emergency management practice.

### Centralized EMS in Shenzhen

Located on the eastern shore of PRD, Shenzhen was established as the first special economic zone in China and has become a fast-growing city known for its vibrant economy and technology industry. The EMS in Shenzhen complies with the *One Planning plus Three Systems* (一案三制) framework, which requires one preceding emergency response plan to be detailed in the later emergency legislative, institutional, and regulatory systems. Within a hierarchical structure at national, provincial, municipal, and district/county levels, roles and responsibilities of a broad range of key stakeholders were defined in the emergency operation plans. From the network perspective, the centralized EMS in Shenzhen has two distinguishable characteristics. Horizontally, government agencies play a dominant role in building and maintaining network relationships with other types of organizations. Public institutions and state-owned enterprises are in practice affiliated to government agencies regardless of whether they have been literally included in the pre-disaster emergency plan. Hierarchically, superior governments have absolute authority over government agencies at lower levels. This means that once the emergency response is activated nationwide, the district/county-level government at the place of emergent incident shall instantly take prompt action to control the potential social damage and immediately report the operational status to the government of the next higher level in a timely manner. As a result, governments at all levels, state-owned enterprises, and public institutions have been bundled into an institutionalized emergency network.

### Decentralized EMS in Hong Kong

Bordering Shenzhen to the north, Hong Kong is a special administrative region of China (HKSAR) with separate economic and political systems from that of mainland China under the “one country, two systems” principle. Serving a population of around 7.4 million, the HKSAR government is led by the Chief Executive (CE), who nominates other principal officials for appointment of the central government. Immediately after the handover in 1997, the HKSAR government developed a Three-Tier EMS to regulate emergency preparedness and response in line with the scope and severity of a given natural disaster. Despite the consistent guidance provided throughout different emergency phases, the Three-Tier System is mainly operated on the basis of intergovernmental coordination and liaison. Specifically, it has explicitly defined the roles and responsibilities of involved emergency response organizations. It resembles the ESF-based system which takes a more collaborative approach to disaster response and allows for more flexibility enabling response organizations to reach out to other interest-related actors in the network to mobilize resources and share information. Moreover, a departmental liaison officer system is executed to ensure direct and proper communication between the Emergency Monitoring and Support Centre (EMSC) and key departments at all levels. Liaison officers who have ample knowledge and expertise within their areas will be called into the EMSC as necessary.

Compared with Shenzhen, the EMS in Hong Kong exhibits a more decentralized and inter-departmental collaborative structure. Nonetheless, it should be noted that these two systems are both conceptually and practically intertwined rather than mutually exclusive. Even the centralized system in Shenzhen has embraced an increasingly collaborative approach to emergency management since the SARS outbreak in 2003. As thus, the contrast between two systems is not absolute—it is rather a continuous than a dichotomous differentiation defined by the extent to which a region’s political-administrative arrangements in emergency management exhibit more coerciveness or more voluntariness. Table 1 presents the context of Typhoon Mangkhut in Shenzhen and Hong Kong.

**Table 1:** The context of Typhoon Mangkhut in Shenzhen and Hong Kong.

	Shenzhen	Hong Kong
Administrative level	Sub-provincial city	Special Administrative Region
Area (*km*^2^)	1,997	1,107
Population	13,026,600	7,486,400
Density of population (/*km*^2^)	6,523	6,763
GDP per capita (*USD*, 2017)	28,647	48,717
Warning Signal	Red alert (highest level)	Hurricane Signal No.10
Economic value loss (*USD*)	1.99 billion (mainland China)	930 million
Affected areas	Philippines, South China, Hong Kong, Macau, Taiwan, Vietnam, Guam, Northern Mariana Islands	

Data on area, population, and GDP per capita were retrieved from sz.gov and gov.hk, respectively.

## Materials and methods

### Ethics statement

All the data collected including government documents, newspaper articles, and social media data are open for the public. Due to the relatively modest data size, data obtained from Sina Weibo and Twitter were manually captured and analyzed complying with the terms and conditions of both platforms.

### Identifying network actors

This study constructs online ECNs with Sina Weibo data of 71 organizational actors in Shenzhen and Twitter data of 38 organizational actors in Hong Kong. As network researchers have emphasized the importance of both theoretical aims and case specificity in delineating social network boundaries [24, 65], online network actors were identified with the following few steps.

First, a content analysis of secondary data obtained from publicly available government documents and six weeks of newspaper articles was conducted to identify organizations involved in the ECN during the response to Typhoon Mangkhut. Most government agencies across the world, either central or local, have executed strict policies of information disclosure over emergency and disaster news. Moreover, news reports of natural disasters are of high transparency and continuity, thus providing opportunities for researchers to identify multiple types and dynamics of ECNs [39, 54, 55].

Government documents including situation reports and after-action reports were captured from government news websites of Shenzhen (www.sz.gov.cn) and Hong Kong (www.news.gov.hk). After carefully reading through these documents, 113 reports were identified from sz.gov and 46 from gov.hk as relevant for the emergency management of Mangkhut (see S1 Table). With respect to newspaper articles, *Southern Metropolis Daily-Shenzhen Edition* (SMD-SZ) and *Shenzhen Evening News* (SEN) were selected to include media coverage from both market-driven and party-affiliated media outlets in Shenzhen; while *Ming Pao* (MP) and *Oriental Daily News* (ODN) were chosen to obtain a representative sample from Hong Kong newspaper market. WiseNews, a widely used Chinese-language database that provides full-text newspaper articles, was employed to search articles on Typhoon Mangkhut that were published in a six-week period starting from two weeks before the disaster to four weeks after (September 2, 2018 to October 13, 2018). The keyword “Mangkhut” (山竹) was used for search queries and after removing duplicates and irrelevant contents, 116 newspaper articles in Shenzhen and 301 in Hong Kong remained for further analysis (see S2 Table).

Second, by means of a systematic content analysis of government documents and newspaper articles, a list of 330 organizations in Shenzhen and 195 in Hong Kong were identified to have participated in the on-the-ground ECN during and immediately after the disaster. Third, among these response organizations, 100 were found to manage verified accounts on Weibo while 43 were found to manage Twitter accounts with a moderate to considerable number of followers (see S3 Table). Finally, after data screening, those accounts engaging in no communication activities in any direct or indirect forms (i.e., tweets, hashtags, mentions, retweets) during the response and initial recovery were further excluded, resulting in a total of 71 organizational accounts in Shenzhen and 38 in Hong Kong. These accounts represent a cross-section of public, for-profit, and non-profit sectors, as well as a collection of emergency collaborative relationships managed by organizations.

### Data collection

The next step is to obtain the research data. All tweets posted by the 71 Shenzhen-based Weibo accounts and the 38 Hong Kong-based Twitter accounts over the aforementioned six-week period were manually captured. These social media data were then broken into three sub-datasets for both regions, each of which represents a two-week observation of online interactions and collaborative activities (T1-preparedness phase: September 2, 2018 to September 15, 2018; T2-response phase: September 16, 2018 to September 29, 2018; T3-recovery phase: September 30, 2018 to October 13, 2018).

During actual disasters, multiple interorganizational ties constructed through various social media features may enable participating actors to share common goals and group norms on account of different levels of engagement in online ECNs. One established means of observing network ties is based on affiliations of organizational actors, for example, shared membership in boards of directors [66], co-authorship in academic publications [67], common references in media reports [68], and collaborative partnership in interlocal government agreements [69] or cooperative initiatives [70]. Following several recent empirical efforts that have applied such an affiliation approach to observing networks in online emergency collaboration [8, 24], this study captures the evidence of interorganizational communication activities operated among network actors through two types of affiliation network relationships: shared hashtags and mention contacts. In each case, the affiliation network includes two modes: organizational actors and hashtags/mentions, and network ties between any two actors are measured in terms of the observed hashtags/mention contacts they share in common. Specifically, hashtags represent actors’ communication practice in organizing words or multi-words phases that categorize and track topics in a tweet (i.e., a tag preceded by the hash mark “#” on Twitter and two hash marks between which a tag presents on Weibo). As thus, links observed through shared hashtags may reflect actors’ intention to follow the same discussion as well as similar online communities discussing the marked topic that interest them [71]. Two organizations were thus connected by means of using common hashtags. On the other hand, organizational actors can mention other users by using the symbol “@” immediately followed by the target username within the text. Links observed through shared mention contacts may refer to actors’ intention to engage in the same conversation, build common connections, or draw awareness within the social media community. Similarly, two organizations were considered connected provided they share common mentions.

It is worth noting that the present study focused on investigating shared contacts as indirect forms of interorganizational communication activities for several practical reasons. First, sharing common contacts with other organizations can reflect how actors promptly take advantage of various social media features to facilitate information flow, enhance the likelihood of substantive collaboration, and engage with the larger stakeholder community [24]. This form of communication can be addressed as strategic communication since it demonstrates “the purposeful use of communication by an organization to fulfill its mission” ([72 p. 3]). Second, although actors sharing common contacts may not be aware of each other, the publicly exhibited mediators provide opportunities for actors to engage in a series of representational collaborative networks to draw awareness from the broader organizational community [73]. Moreover, the inspection mechanism afforded by the social media application ensures that contacted users receive a notification from social media alert when they are mentioned or replied to. It can be therefore assumed that contacted users are more likely to notice those communication activities which give rise to sustained inspection and continuous information exchange between organizational accounts. Third, online ECNs established on the basis of direct ties among organizational actors using a similar set of social media features (e.g., mentions, retweets) were found to be loosely connected with an extremely large proportion of isolated nodes. The highly skewed network structure together with the low network density were likely to bias the results of data analysis.

Several steps were then carried out to generate network matrices for two regions, respectively. First, all unique hashtags (N_SZ_ = 740; N_HK_ = 1244) and mentioned contacts (N_SZ_ = 974; N_HK_ = 373) used by organizational accounts were identified from the raw data (see S4 Table). The next step was to generate six two-mode matrices (3 × 2) for both regions to measure each established online ECN over a two-week period of observation. In these matrices, the rows represented organizational accounts while the columns represented unique hashtags and mentioned contacts, respectively. For all 12 binary network matrices, a “1” was entered into the cell when a given organization used a particular shared contact in their tweets during the response to Typhoon Mangkhut and a “0” was entered into the cell otherwise. In addition, the aggregate of shared contacts was not accounted due to the potential bias of multi-valued networks and the relatively short period of network observation. Finally, 12 two-mode matrices were converted to 24 one-mode square matrices. For the current concern, only those one-mode organization-by-organization matrices representing the shared communication relationships between two network actors were used for further analysis (see S1 and S2 Files).

### Data analysis

#### Organizational attributes

Three categorical attributes were defined and manually assigned to each sampled organizational account, including organization type, geographic scale, and geo-location. Specifically, network actors in both regions were categorized as to whether they belong to a government agency, a public institution (e.g., university, hospital, blood station), a for-profit business (e.g., state-owned, private, and foreign enterprises), an armed force (e.g., PLA Hong Kong Garrison, People’s Armed Police Forces at local levels), or an NGO/NPO. The addition of armed forces to this categorization reflects unique features of China’s EMS that were not considered in prior network studies conducted under the federal system. Geographic scale was coded as the coverage within which an organization operates following five categories: central-level, provincial-level, municipal-level, district/county-level, and below district/county-level. As an ECN incorporates social sectors other than government agencies, geographic scale proves to be more suitable than government jurisdiction in describing the footprint of a network actor’s emergency management practice [8, 74]. It should be noted that the geographic scale was not accounted for the Hong Kong data because nearly all identified actors operate within the HKSAR. In addition, the geo-location was measured by the place in which organizational actors are located or headquartered. Network actors were classified into five groups in line with the jurisdictional division of Shenzhen: Shenzhen East (Longgang District, Yantian District, Pingshan District, Dapeng New Area, and Shen-Shan Special Cooperation Zone), Shenzhen West (Baoan District, Guangming District, and Nanshan District), Shenzhen South (Futian District and Luohu District), Shenzhen North (Longhua District), and non-local; and five groups according to the jurisdictional division of Hong Kong: Hong Kong Island, New Territories, Kowloon Peninsula, Islands District, and non-local.

The organizational attribute files were then transformed to network matrices using the attribute/matrix converting function afforded in UCINET [75]. Table 2 presents the attributes of identified organizational actors in Shenzhen and Hong Kong.

**Table 2:** Attributes of online participating organizations in Shenzhen and Hong Kong.

	Shenzhen (Weibo)	Hong Kong (Twitter)
***Organization type***		
Government agency	42 (59.2%)	4 (10.5%)
Public institution	9 (12.7%)	10 (26.3%)
Business	14 (19.7%)	12 (31.6%)
Military	1 (1.4%)	--
NGO/NPO	5 (7.0%)	12 (31.6%)
***Geographic scale***		
Central	2 (2.8%)	--
Provincial	4 (5.6%)	--
Municipal (SAR level)	35 (49.3%)	38 (100%)
District/County-level	29 (40.8%)	--
Below district/county-level	1 (1.4%)	--
***Geo-location***		
South (Hong Kong Island)	35 (49.3%)	19 (50.0%)
North (New Territories)	2 (2.8%)	4 (10.5%)
East (Kowloon Peninsula)	12 (16.9%)	12 (31.6%)
West (Islands District)	16 (22.5%)	3 (7.9%)
Non-local	6 (8.5%)	--
Total	71	38

#### MR-QAP analyses

Hypotheses and research questions posited above were examined using the multiple regression with quadratic assignment procedure (MR-QAP). Specifically, MR-QAP was conducted to analyze the relationships among online preparedness networks, online response networks, and online recovery networks (H1-H4) as well as the conditional effect of EMSs on the longitudinal network evolution (H5 and H6). As an inferential statistical procedure, QAP performs random permutation on identical network matrices of the same group of actors and investigates the association between matrices through calculating their standard errors [76]. It differs from conventional statistical techniques (e.g., linear and logistic regression) as it does not assume data independency, thus making this method suitable for analyzing network data. MR-QAP resembles multiple regression analyses and can be used to examine the predictive power of both structural and attribute factors on the network relationship, which is often considered as the dependent variable. In addition, three homophily variables (i.e., organization type, geographic scale, and geo-location) were set as covariates to control for potential extraneous effects. MR-QAP analyses were conducted on UCINET [75].

## Results

### Descriptive statistics

Whole network characteristics of online ECNs across both EMSs were firstly reported. As shown in Table 3, both hashtag networks and mention networks within the centralized EMS were consistently denser than their counterparts in the decentralized EMS as the disaster evolved over time, suggesting that in Shenzhen, network actors forged more online communication ties with other organizations throughout the emergency management cycle. In both EMSs, online recovery networks were sparser than online preparedness networks and online response networks. In the centralized EMS, the degree centralization index of online recovery networks was lower than that of preparedness and response networks. In contrast, the opposite was true for the decentralized EMS wherein the centralization score of the recovery network characterized by shared hashtags was slightly higher than that of its two counterparts. This implies that the network tendency to center around a couple of key actors may be subject to structural variations.

**Table 3:** Descriptive statistics of online ECNs.

Online networks	Density		Centralization	
	Hashtag	Mention	Hashtag	Mention
Shenzhen: Centralized EMS				
Online preparedness network	.192	.114	.346	.383
Online response network	.186	.206	.324	.346
Online recovery network	.093	.078	.257	.272
Hong Kong: Decentralized EMS				
Online preparedness network	.131	.064	.290	.275
Online response network	.131	.063	.318	.276
Online recovery network	.075	.044	.320	.239

**Fig 1:**
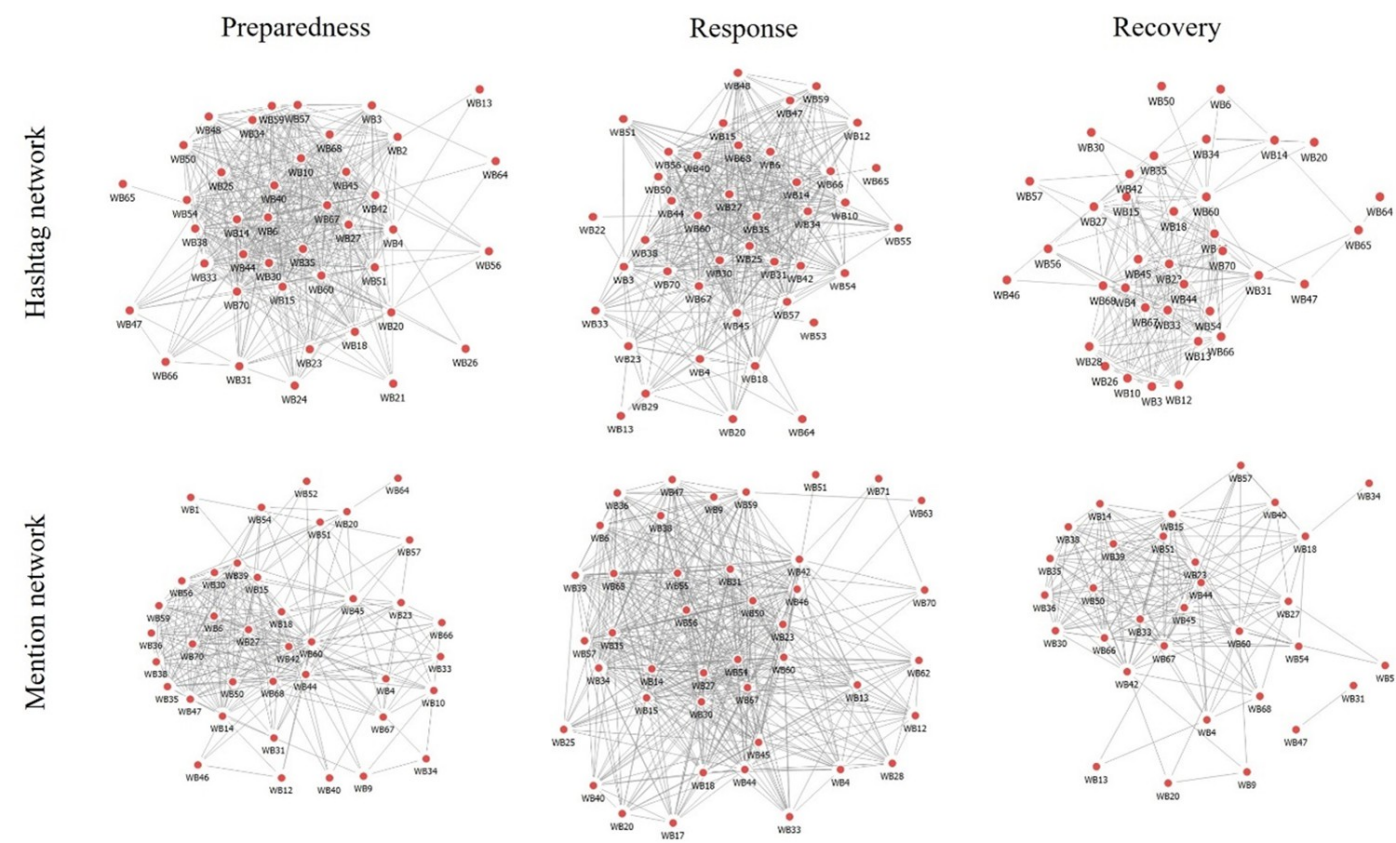
Structural characteristics of two types of online networks in the centralized EMS in Shenzhen. Isolated nodes without any shared hashtag or mention contact with other network actors are excluded for visualization parsimony.

**Fig 2:**
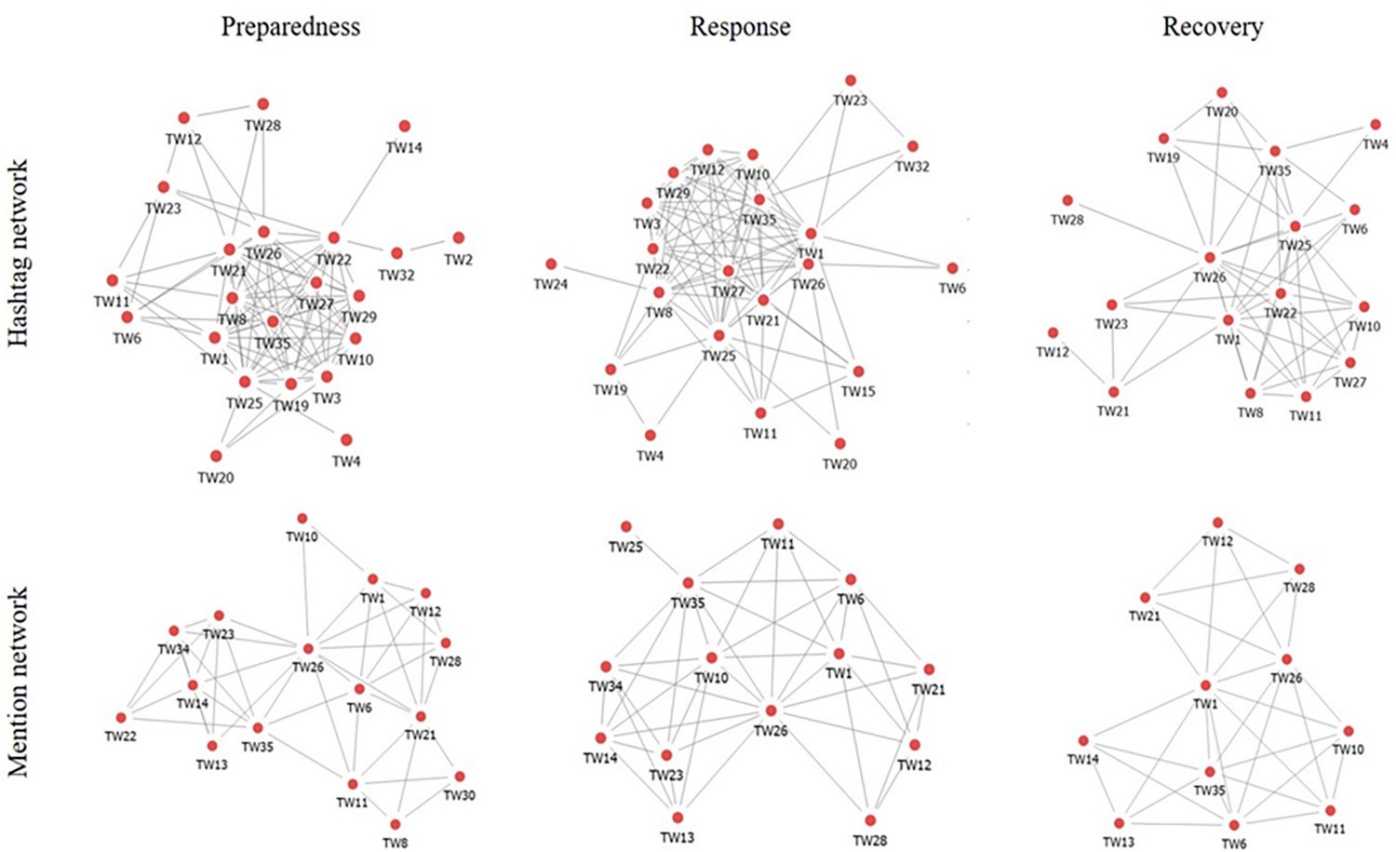
Structural characteristics of two types of online networks in the decentralized EMS in Hong Kong. Isolated nodes without any shared hashtag or mention contact with other network actors are excluded for visualization parsimony.

Structural characteristics of online ECNs were visualized for Shenzhen data and Hong Kong data in Figs 1 and 2, respectively. Longitudinal variations can be more explicitly observed that online preparedness and response networks were denser than recovery networks across both EMSs.

### QAP correlations among multiple networks

QAP correlations among online preparedness networks, response networks, and recovery networks were reported to cast a preliminary spotlight on the longitudinal multiplexity of online ECNs. Since the network data (i.e., one-mode matrices) analyzed in this study are binary, Jaccard correlation coefficients were used to evaluate the QAP correlations among multiple networks. Specifically, Jaccard (similarity) coefficients measure the similarity of two networks by comparing the total number of common dyads to the total number of distinct dyads [76]. The coefficient values range from 0 to 1 and the higher the coefficients, the more similar the two networks. As shown in Table 4, online preparedness ties were positively associated with online collaborative ties during disaster response and immediate recovery in both EMSs. Within the centralized EMS in Shenzhen, the Jaccard correlation coefficients between preparedness and response networks and between preparedness and recovery networks were 0.571 (hashtag)/0.370 (mention) and 0.255 (hashtag)/0.339 (mention), respectively. Correlations between response and recovery networks were 0.265 (hashtag)/0.255 (mention). All these coefficients were significantly positive at .001 level. Similarly, the Jaccard correlation coefficients were significantly positive between preparedness networks and response networks (0.614/0.618), between preparedness networks and recovery networks (0.422/0.382), and between response networks and recovery networks (0.381/0.531) within the decentralized EMS in Hong Kong. By and large, compared with the centralized EMS, correlations between multiple networks were relatively stronger in the decentralized EMS. The relationship between online preparedness and response networks was consistently higher than two other comparisons of network similarities. Nonetheless, the contrast of network similarities varied depending on the forms of online communication activities (i.e., shared hashtag or mention contacts).

**Table 4:** QAP correlations between three types of online ECNs.

Online networks	Online response network		Online recovery network	
	Hashtag	Mention	Hashtag	Mention
Shenzhen: Centralized EMS				
Online preparedness network	.571***	.370***	.255***	.339***
Online response network			.265***	.255***
Hong Kong: Decentralized EMS				
Online preparedness network	.614***	.618***	.422***	.382***
Online response network			.381***	.531***

Jaccard correlation coefficients are reported because the network matrices are binary. QAP = quadratic assignment procedure.

^***^p < .001.

### MR-QAP analyses

Although QAP correlations appear easy and straightforward to interpret, they may present erroneous results due to their sensitivity to small sample sizes. Therefore, MR-QAP was conducted to further test the evolving correlations among multiple online ECNs. By doing so, the effects of correlations between preparedness and response networks can be separated and more importantly, the control variables (i.e., homophily effects) can be considered to obtain a more comprehensive picture of network dynamics. Double Semi-Partialing (DSP) permutation method, which is robust against a broad spectrum of statistical biases [77], was used to reduce the sensitivity of MR-QAP analyses to network autocorrelation and collinearity.

H1 posits that organizational actors’ online preparedness networks positively correlate with their online response networks. As presented in Table 5, online collaborative ties within response networks were set as dependent variable in Model I. The regression coefficients of online preparedness ties for predicting response ties were 0.643 (hashtag)/0.463 (mention) within the centralized EMS and 0.724 (hashtag)/0.748 (mention) within the decentralized EMS. All coefficients were significantly positive at .001 level, thus supporting H1 that online collaborative ties forged at the preparedness phase were conducive to the formation of collaborative ties during the response to Typhoon Mangkhut.

**Table 5:** Model I: MR-QAP results for predicting online response networks.

	Model I: Centralized EMS in Shenzhen				Model I: Decentralized EMS in Hong Kong			
	Hashtag		Mention		Hashtag		Mention	
Organization type	.246***	.082**	.235***	.155**	.019	.005	.045	-.046
Geographic scale	-.011	-.023	-.018	-.007	--	--	--	--
Geo-location	-.016	.004	.069	.052	.051	.020	.130*	.056
Online preparedness ties	--	.643***	--	.463***	--	.724***	--	.748***
Intercept	.000	.000	.000	.000	.000	.000	.000	.000
*R* ^2^	.061	.447	.058	.265	.003	.526	.019	.565
Adjusted *R*^2^	.061	.447	.057	.265	.002	.525	.018	.564
No. of observations	4,970	4,970	4,970	4,970	1,406	1,406	1,406	1,406
No. of permutations	5,000	5,000	5,000	5,000	5,000	5,000	5,000	5,000

The dependent variable in the multiple regression analysis is the online collaborative ties within response networks. Results for the base model entering only homophily variables are presented on the left side of each regression. Coefficients of the geographic scale are ignored for Hong Kong data because all organizational accounts identified from Hong Kong are locally operated and managed. All input variables are standardized.

^*^p < .05.

^**^p < .01.

^***^p < .001.

H2 examines the association between online preparedness networks and recovery networks. As shown in Table 6, online collaborative ties within recovery networks were set as dependent variable in Model II. The regression coefficients of online preparedness ties were 0.169 (hashtag)/0.357 (mention) within centralized networks and 0.408 (hashtag) within decentralized networks and significantly positive at .001 level. However, the coefficient of preparedness ties built upon shared mention contacts was not statistically significant for the Hong Kong data. Hence, H2 was partially supported.

**Table 6:** Model II: MR-QAP results for predicting online recovery networks.

	Model II: Centralized EMS in Shenzhen				Model II: Decentralized EMS in Hong Kong			
	Hashtag		Mention		Hashtag		Mention	
Organization type	.237***	.141**	.220***	.117**	-.023	-.035	-.036	-.072**
Geographic scale	.005	.004	.041	.053*	--	--	--	--
Geo-location	-.052	-.044	-.005	-.031	.028	-.001	.163**	.074*
Online preparedness ties	--	.169***	--	.357***	--	.408***	--	.067
Online response ties	--	.214***	--	.182***	--	.233***	--	.632***
Intercept	.000	.000	.000	.000	.000	.000	.000	.000
*R* ^2^	.060	.173	.051	.264	.001	.359	.028	.485
Adjusted *R*^2^	.059	.172	.050	.264	-.000	.357	.026	.484
No. of observations	4,970	4,970	4,970	4,970	1,406	1,406	1,406	1,406
No. of permutations	5,000	5,000	5,000	5,000	5,000	5,000	5,000	5,000

The dependent variable in the multiple regression analysis is the online collaborative ties within recovery networks. Results for the base model entering only homophily variables are presented on the left side of each regression. Coefficients of the geographic scale are ignored for Hong Kong data because all organizational accounts identified from Hong Kong are locally operated and managed. All input variables are standardized.

^*^p < .05.

^**^p < .01.

^***^p < .001.

H3 assumes a positive relationship between online response networks and recovery networks. As Table 6 shows, the regression coefficients of collaborative ties formed at the stage of disaster response for predicting the collaborative ties during immediate disaster recovery were 0.214 (hashtag)/0.182 (mention) within the centralized system and 0.233 (hashtag)/0.632 (mention) within the decentralized system. All reported coefficients were significantly positive at .001 level. Therefore, H3 was supported that organizational actors’ online response ties contributed to the formation of post-disaster recovery ties.

H4 assumes that the correlations between online response and recovery networks are higher than those between the other two pairs of online networks. As shown in Model I together with Model II, the regression coefficients of preparedness ties for predicting response ties were consistently higher than those of preparedness ties for predicting recovery ties and of response ties for predicting recovery ties. H4 was thus unsupported.

Structural constraints represented by political-administrative arrangements were further examined as a context-specific factor in uncovering the logics of online network formation and evolution. Descriptive statistics presented in Table 3 showed that with the exception of the hashtag network built during disaster recovery, centralization scores of all other five types of online networks were higher for the Shenzhen data than those for the Hong Kong data. This contrast corroborated the current division of centralized versus decentralized coordination mechanism against the social media context. That is, the structural constraints for emergency collaboration on the ground can apply to online interorganizational interactions such that Shenzhen demonstrated a more centralized online network structure, whereas Hong Kong exhibited a more decentralized network pattern for emergency management.

More specifically, H5 assumes that online preparedness ties within the centralized EMS have stronger predictive power in the formation of online response ties than do their counterparts within the decentralized EMS. As presented in Table 5 (Model I), the regression coefficients of preparedness ties for predicting response ties within the centralized system were 0.643 (hashtag)/0.463 (mention), which were lower than their counterparts within the decentralized system (0.724 for the hashtag network; 0.748 for the mention network). Therefore, H5 was not supported.

H6 posits that online response networks within the decentralized EMS have stronger predictive power in the formation of online recovery networks than do their counterparts within the centralized EMS. As shown in Table 6 (Model II), compared with the centralized system within which regression coefficients were 0.214 for the hashtag network and 0.182 for the mention network, online collaborative ties forged at the response phase had higher predictive power in the formation of post-disaster recovery ties within the decentralized system (regression coefficients were 0.233 for the hashtag network and 0.632 for the mention network). As thus, H6 was supported.

Regarding the homophily variables (RQ1) which were controlled for the current concern, regression coefficients of the organization type similarity with the centralized EMS were 0.082 (hashtag)/0.155 (mention) for Model I and 0.141 (hashtag)/0.117 (mention) for Model II. All these coefficients were significantly positive at .01 level, suggesting that organizational actors of the same type may share common goals and network constraints and thus are more likely to collaborate with one another in mobilizing resources and exchanging information in the disaster response and post-disaster recovery. The openness and transparency of communication activities on social media applications may make it easier for communication managers to seek partners sharing similar organizational values and identities, therefore strengthening such homophily effects. However, the opposite was evidenced for the Hong Kong data that the regression coefficients of the organization type similarity within the decentralized system were mostly negative. This indicates that throughout the Typhoon Mangkhut, online interorganizational networks in Hong Kong may exhibit a more horizontal structural pattern with more cross-sectoral information sharing and resource mobilization than their counterparts in Shenzhen.

## Discussion

Results of the longitudinal multiplexity, especially the significantly positive relationships among online ECNs across disaster stages of preparedness, response, and recovery in both regions, have reiterated the notion that emergency management network represents a social structure of strong embeddedness [11]. Although extant research on emergency management taps into the concept of structural embeddedness by categorizing emergency collaborations in a functional manner (e.g., [52, 78]), this study uses a longitudinal observation to demonstrate a pressing need for emergency researchers and frontline communication managers to understand the dynamic relationships among online ECNs across different time periods and to explore potential opportunities to facilitate collaboration on a broader scale.

The findings of longitudinal multiplexity also speak to prior research that has emphasized the importance of preexisting relationships in intergovernmental and cross-sectoral collaboration and coordination (e.g., [10, 38]). For instance, the density of hashtag networks at the preparedness stage (Shenzhen: 0.192; Hong Kong: 0.131) is close to or even slightly higher than those at the response stage (Shenzhen: 0.186; Hong Kong: 0.131) in both EMSs investigated. This suggests that in their daily operations, organizational actors reach out to keep regular online connections with other partners staying active in or beyond the domain of emergency management. These preestablished collaborative ties can be understood as the stock of social capital deriving from the shared history of collective action [79]. Organizations build social capital through using either bonding strategies to form ties to partners that are closely connected with one another (i.e., bonding social capital) or bridging strategies to form ties to centrally connected partners (i.e., bridging social capital). This stock of social capital primarily accumulated through online preparedness ties can further enhance mutual trust and commitment among network actors, thus alleviating their perceived collaboration risks and increasing their tendency to build and sustain future collaborations during disaster response and recovery.

The potential gap between formal and informal coordination mechanisms has long attracted scholarly attention in the field of emergency management. The formal-informal division was typically delineated in accordance with planned versus actual response networks [13, 80]. Situated in the social media environment, this study focuses on the evolving features of formal-informal division by arguing that online networks constructed at the preparedness stage may resemble informal friendship networks in providing communication channels for actors to share information and strengthen capacity [10, 28], while those built during disaster response and recovery generally incorporate more formal collaboration and coordination. It is intriguing that the correlations between informal preparedness networks and formal response networks are consistently higher than those between the other two pairs of online networks. This implies that formal and informal coordination mechanisms are not mutually exclusive in practice and can be understood using, for example, different levels of complexity and authority to better overcome dynamic collaboration dilemmas. Granted, whether and how actors make decisions to collaborate with each other can evolve over time. Only through exploring the combination and evolution of coordination mechanism can researchers and practitioners achieve a better understanding of the relationships between informal and formal network patterns and how these patterns jointly resolve collective action problems.

This study also incorporates EMS into the conceptual framework as an external constraint to examine its effects on how organizational actors make coordination mechanism choices to overcome collaboration problems. Specifically, EMS in which actors operate and collaborate determine the extent to which they have to forgo their autonomy for the sake of enhancing mutual trust and facilitating interorganizational commitments. In other words, autonomy costs incurred on actors vary along with the level of authority imposed by the EMS. The findings demonstrate that structural variations unfolded in EMSs are crucial for deciphering the longitudinal multiplexity of online ECNs across different political-administrative contexts. More specifically, this study finds evidence for the assumption that online response networks within the decentralized system can better predict the formation of online recovery networks than do their counterparts within the centralized system. This can be explained by the advantage of decentralized network structures in facilitating cross-sectoral and interjurisdictional interactions and motivating actors to reach out to partners with common goals and shared values [81]. As argued earlier, the evolution of network structures during the transition from disaster response to recovery is typically characterized and constrained by resource scarcity. Apart from the low autonomy costs incurred on involved actors, the decentralized system exhibits more flexibility and voluntariness to foster online network changes during this period because of its effectiveness to mitigate resource insufficiency and coordination risks by securing access to nonoverlapping information and helping actors identify mutually beneficial circumstances.

In contrast, this study finds no evidence for the assumption that online preparedness networks within the centralized system have stronger predictive power in the formation of online response networks than do their counterparts within the decentralized system. One plausible explanation is that online information sharing on social media applications examined in the present study has introduced substantial changes to the communication and coordination among organizational actors. On the one hand, the use of social media applications provides opportunities for communicators to transcend sectoral and geographic boundaries to reach a larger community and engage in conversations with target organizations in a direct and dialogic manner [62, 71]. The multiple interorganizational ties constructed through diverse features afforded by social media may enable actors to share common goals and values on account of different levels of participation in the online ECNs. Hence, actors connected through online activities may have weak awareness of hierarchical constraints exerted by the centralized network structure. On the other hand, social media applications provide information communication channels and features for actors to reduce uncertainty-induced decision costs, especially in seeking and sharing timely information (i.e., information cost) and reaching mutual agreements (i.e., negotiation costs). Moreover, although the limited cognitive capacity prevents emergency managers from consuming all relevant emergency information on social media, the transparency and openness of online communication activities may lead to a new form of monitoring mechanism for mutual commitment. For example, actors receive an automatic notification whenever they are mentioned or retweeted (with a reply). These features may contribute to a sustained or even threaded conversation once the interactions draw continued attention and facilitate information monitoring and exchanging among organizational accounts. It is thus difficult for collaborators to conceal their opportunistic behaviors such as information withdrawal and non-response. All these possibilities are likely to lower the efficiency of the centralized system in fostering online network changes during the transition from disaster preparedness to response, which is characterized by an acute increase of complexity and uncertainty.

### Practical implications

Emergency managers and practitioners can also find some takeaways from this study. First, the significance of preexisting relationships also extends to emergency management practices. After the Great Flood of 1993 that occurred in Midwestern areas of the U.S., a comparison of five communities indicated that immediate aid and assistance at the response stage were available from public and private sources in all communities, but the situation differed greatly during disaster recovery [82]. In communities with preexisting interorganizational networks, local leaders and organizations actively shouldered the responsibility of reconstruction and rebuilding, thus ensuring a sustainable post-disaster recovery. In the absence of preestablished collaborative ties, however, self-organized emergency management teams failed to retain residents’ enthusiasm of collaboration as the flood crisis decayed because they lacked collective learning experiences which essentially contributed to the reconstruction progress. Results from the present study once again uphold the importance of playing out in early emergency preparedness because a wide variety of joint planning activities, training programs, and drills may help enhance collective learning and develop online collaborative ties during disaster response and recovery.

Second, there has long been a heated debate among emergency researchers and practitioners about whether a centralized/coercive power structure is more appropriate for managing emergencies or a decentralized/voluntary power structure prevails (e.g., [53, 83]). This study complements prior research in clarifying the comparison between centralized and decentralized structural constraints by furthering a contingency perspective to explore their varying effects on online network changes as the disaster evolves over time. The decentralized network structure appears more suitable to manage the disaster concerned in this study because it incurs lower autonomy costs while illustrating more effectiveness in facilitating online networks changes during both transitional periods. Nonetheless, the findings exhibit that the correlations between preparedness and response networks and between response and recovery networks are strong in both EMSs, even though they are stronger in the decentralized system. Also, it remains neither clear nor systematic about the extent to which the cross-platform divergence between Twitter and Weibo would influence the results of this study. Hence, more efforts are expected to be made to explicate how each system copes with collective action problems differently against the social media environment and what contributes to the priority of one system over the other.

Moreover, global crises represented by the Covid-19 pandemic have also posed rapidly shifting challenges for leadership, which refers to the ability of top executives and senior managers to unite and persuade a group of individuals and organizations to collaborate and coordinate effectively in the pursuit of collective goals. These emerging challenges, which require leaders in all industries to strategically pivot amid disrupted operations, to promptly embrace a social media mindset, and to effectively manage scattered and remote workforce, are likely to widen the leadership gap between leaders who are purposefully prepared for constantly changing circumstances and those who are not. Findings obtained from this study have crucial implications for leaders to reassess priorities across different phases of a crisis and continuously adapt to a climate of purpose and commitment. Learning and development (L&D) professionals and teams are also expected to help cultivate specific capabilities and skills for response organizations to close the leadership gap in the face of extreme complexities and uncertainties.

### Limitations and future directions

Several limitations of this study should be pointed out. First, the temporal dimension represented by decisions about how to demarcate three disaster phases may bias online ECN boundaries. Although the temporal cutting points were set in line with the case specificity and personal observations of Typhoon Mangkhut, it should be recognized that network dynamics are subject to how online ECNs are operationalized over time. For example, it is plausible that a in a catastrophic event with a much larger scale than Mangkhut, the frequency, durability, and distribution of online interorganizational interactions across different disaster phases would differ greatly such that actors tend to forge ties to foster a sustainable post-disaster recovery, and more importantly, the correlation between formal networks built during disaster response and recovery would be greater than that between informal and formal networks. Future studies are expected to either conduct sensitivity tests to remedy this limitation or employ a more innovative research design in collecting longitudinal online network data.

Second, the current investigation primarily focuses on collective efforts at (inter)organizational levels, while overlooking individual desires and strategic motivations of emergency managers, especially frontline communicators who are actually responsible for managing routinized emergency operations and making improvised decisions on behalf of their organizations and departments on social media platforms. Apart from organizational preferences and response capacities, it merits attention that communication managers’ personal mindset in aligning communication objectives with collaborative goals during emergencies may influence the types of strategy they use to shape collective actions [84]. In this regard, further studies can benefit from retaining the present explanatory pattern but maintaining a certain level of openness and adaptability to incorporate multiple individual motivations.

Finally, despite the efforts made to examine the longitudinal multiplexity and exogenous factors (i.e., EMSs, homophily effects) possibly influencing actors’ coordination mechanism choices, the present investigation suffers from lacking empirical evidence of the effectiveness and performance of collective action, and accordingly, how network outcomes can be improved. To address challenges posed by data availability and an overall lack of direct measures, an emerging body of literature has utilized simulation-based approaches to evaluate network performance and outcomes with crucial indicators such as frequency, speed, and efficacy of information dissemination [85, 86]. Such techniques can broaden the scope of operationalization of network performance and lend researchers methodological support that supplements traditional approaches (e.g., structured/semi-structured survey, text analysis, interview).

## Supporting information

S1 Table Government documents (situation reports and after-action reports) identified from the government news website of Shenzhen/Hong Kong. (DOCX)

S2 Table Newspaper articles identified from Shenzhen/Hong Kong-based newspapers. (DOCX)

S3 Table Organizational actors identified from Shenzhen/Hong Kong on Weibo/Twitter. (DOCX)

S4 Table Hashtags and mention contacts identified from tweets posted by Shenzhen/Hong Kong-based organizational actors. (DOCX)

S1 File One-mode organization-by-organization matrices built upon Shenzhen data. (XLS)

S2 File One-mode organization-by-organization matrices built upon Hong Kong data. (XLS)

## References

[1] Bryer TA, Zavattaro SM. Social media and public administration: theoretical dimensions and introduction to the symposium. Adm Theory Prax. 2011;33(3):325–40.

[2] Castillo C. Big crisis data: social media in disasters and time-critical situations. New York: Cambridge University Press; 2016.

[3] Chatfield AT, Reddick CG. All hands on deck to tweet #sandy: networked governance of citizen coproduction in turbulent times. Gov Inf Q. 2018;35(2):259–72.

[4] Hughes AL, Palen L. Twitter adoption and use in mass convergence and emergency events. Int J Emerg Manag. 2009;6(3–4):248–60.

[5] Wukich C, Mergel I. Reusing social media information in government. Gov Inf Q. 2016;33(2):305–12.

[6] Palen L, Hughes AL. Social media in disaster communication. In: Rodriguez H, Donner W, Trainor JE, editors. Handbook of disaster research. New York: Springer; 2018. p. 497–518.

[7] Plotnick L, Hiltz SR. Barriers to use of social media by emergency managers. J Homel Secur Emerg Manag. 2018;13(2):247–77.

[8] Wukich C, Hu Q, Siciliano MD. Cross-sector emergency information networks on social media: online bridging and bonding communication patterns. Am Rev Public Adm. 2019;49(7):825–39.

[9] Scott J. Social network analysis: a handbook. 3rd ed. Los Angeles: Sage Publications; 2013.

[10] Kapucu N, Hu Q. Understanding multiplexity of collaborative emergency management networks. Am Rev Public Adm. 2016;46(4):399–417.

[11] Kim TY, Oh H, Swaminathan A. Framing interorganizational network change: a network inertia perspective. Acad Manage Rev. 2006;31(3):704–20.

[12] Doerfel ML, Chewning LV, Lai CH. The evolution of networks and the resilience of interorganizational relationships after disaster. Commun Monogr. 2013;80(4):533–59.

[13] Kapucu N, Demiroz F. Measuring performance for collaborative public management using network analysis methods and tools. Public Perform Manag Rev. 2011;34(4):549–79.

[14] Ingold K. How to create and preserve social capital in climate adaptation polices: a network approach. Ecol Econ. 2017;131:414–24.

[15] Jung K, Song M, Park HJ. The dynamics of an interorganizational emergency management network: interdependent and independent risk hypotheses. Public Adm Rev. 2019;79(2):225–35.

[16] Bodin Ö, Nohrstedt D. Formation and performance of collaborative disaster management networks: evidence from a Swedish wildfire response. Glob Environ Change. 2016;41:183–94.

[17] Kim K, Yoon HY, Jung K. Resilience in risk communication networks: following the 2015 MERS response in South Korea. J Contingencies Crisis Manag. 2017;25(3):148–59. 10.1111/1468-5973.12180 PMC7167056 40476947

[18] Zeemering ES. An agenda for comparing local governance and institutional collective action in Canada and the United States. Urban Aff Rev. 2019;55(3):858–86.

[19] Feiock RC. Metropolitan governance: conflict, competition, and cooperation. Washington: Georgetown University Press; 2004.

[20] Feiock RC. The institutional collective action framework. Policy Stud J. 2013;41(3):397–425.

[21] Kim SY, Swann WL, Weible CM, Bolognesi T, Krause RM, Park AY, et al. Updating the Institutional Collective Action Framework. Policy Stud J. 2022;50(1):9–34.

[22] Borgatti SP, Everett MG, Johnson JC. Analyzing social networks. Los Angeles: Sage Publications; 2013.

[23] Provan KG, Milward HB. Do networks really work? A framework for evaluating public‐sector organizational networks. Public Adm Rev. 2001;61(4):414–23.

[24] Lai CH, She B, Tao CC. Connecting the dots: a longitudinal observation of relief organizations’ representational networks on social media. Comput Human Behav. 2017;74:224–34.

[25] Gurman TA, Ellenberger N. Reaching the global community during disasters: findings from a content analysis of the organizational use of Twitter after the 2010 Haiti earthquake. J Health Commun. 2015;20(6):687–96. doi: 10.1080/10810730.2015.1018566 25928401

[26] Zhang H. Emerging risks, social media, and crisis management in China: a comparative study on the cases of the Wenzhou high speed train accident and the Jiaoji railway accident. Cambridge: Ash Center, Harvard Kennedy School; 2013.

[27] Krackhardt D, Hanson JR. Informal networks. Harv Bus Rev. 1993;71(4):104–11. 10127036

[28] Adams LM. Exploring the concept of surge capacity. Online J Issues Nurs. 2009 Mar 31;14(2). doi: 10.3912/OJIN.Vol14No02PPT03

[29] Isett KR, Mergel IA, LeRoux K, Mischen PA, Rethemeyer RK. Networks in public administration scholarship: understanding where we are and where we need to go. J Public Adm Res Theory. 2011;21 Suppl 2:157–73.

[30] Granovetter M. Economic action and social structure: the problem of embeddedness. Am J Sociol. 1985;91(3):481–510.

[31] Uzzi B. Social structure and competition in interfirm networks: the paradox of embeddedness. Adm Sci Q. 1997;42(2):35–67.

[32] Granovetter M. Problems of explanation in economic sociology. In: Nohria N, Eccles R, editors. Networks and organizations: structure, form, and action. Boston: Harvard Business School Press; 1992. p. 25–56.

[33] Doerfel ML, Taylor M. The story of collective action: the emergence of ideological leaders, collective action network leaders, and cross-sector network partners in civil society. J Commun. 2007;67(6):920–43.

[34] Duysters G, Lemmens C. Alliance group formation enabling and constraining effects of embeddedness and social capital in strategic technology alliance networks. Int Stud Manag Organ. 2003;33(2):49–68.

[35] Gulati R, Gargiulo M. Where do interorganizational networks come from? Am J Sociol. 1999;104(5):1439–93.

[36] Kapucu N, Garayev V. Designing, managing, and sustaining functionally collaborative emergency management networks. Am Rev Public Adm. 2012;43(3):312–30.

[37] McCammon HJ, Van Dyke N. Applying qualitative comparative analysis to empirical studies of social movement coalition formation. In: Van Dyke N, McCammon HJ, editors. Strategic alliances: coalition building and social movements. Minneapolis: University of Minnesota Press; 2010. p. 292–315.

[38] Kapucu N, Hawkins CV, Rivera FI. Disaster resiliency: interdisciplinary perspectives. New York: Routledge; 2013.

[39] Kapucu N. Public‐nonprofit partnerships for collective action in dynamic contexts of emergencies. Public Adm. 2006;84(1):205–20.

[40] Gonzalez-Bailon S. Opening the black box of link formation: social factors underlying the structure of the web. Soc Networks. 2009;31(4):271–80.

[41] Sullivan J, Xie L. Environmental activism, social networks and the internet. China Q. 2009;198:422–32.

[42] Robinson SE, Eller WS, Gall M, Gerber BJ. The core and periphery of emergency management networks. Public Manag Rev. 2013;15(3):344–62.

[43] Rubin CB, Popkin R. Disaster recovery after Hurricane Hugo in South Carolina (No. 69). Boulder: Natural Hazards Research and Applications Information Center, Institute of Behavioral Science, University of Colorado; 1990.

[44] Smith GP. Planning for post-disaster recovery: a review of the United States disaster assistance framework. Washington: Island Press; 2012.

[45] Berke PR, Kartez J, Wenger D. Recovery after disaster: achieving sustainable development, mitigation and equity. Disasters. 1993;17(2):93–109. doi: 10.1111/j.1467-7717.1993.tb01137.x 20958760

[46] Comfort LK, Birkland TA, Cigler BA, Nance E. Retrospectives and prospectives on Hurricane Katrina: five years and counting. Public Adm Rev. 2010;70(5):669–78.

[47] Mader GG, Tyler MB. Rebuilding after earthquakes: lessons for planners. Portola Valley: Spangle Associates; 1991.

[48] Tavares AF, Feiock RC. Applying an institutional collective action framework to investigate intermunicipal cooperation in Europe. Perspect Public Manag Gov. 2017;1(4):299–316.

[49] Kapucu N, Garayev V. Structure and network performance: horizontal and vertical networks in emergency management. Adm Soc. 2016;48(8):931–61.

[50] Anderson AI, Compton D, Mason T. Managing in a dangerous world—the national incident management system. Eng Manag J. 2004;16(4):3–9.

[51] Roberts P, Ward R, Wamsley G. The evolving federal role in emergency management: policies and processes. In: Rubin CB, editor. Emergency management: the American experience, 1900–2010. Boca Raton: CRC Press; 2012. p. 247–76.

[52] Moynihan DP. The network governance of crisis response: case studies of incident command systems. J Public Adm Res Theory. 2009;19(4):895–915.

[53] Birkland TA. Disasters, lessons learned, and fantasy documents. J Contingencies Crisis Manag. 2009;17(3):146–56.

[54] Comfort LK, Haase TW. Communication, coherence, and collective action: the impact of Hurricane Katrina on communications infrastructure. Public Works Manag Policy. 2006;10(4):328–43.

[55] Hu Q, Knox CC, Kapucu N. What have we learned since September 11, 2001? A network study of the Boston marathon bombings response. Public Adm Rev. 2014;74(6):698–712.

[56] Feiock RC, Scholz JT. Self-organizing federalism: collaborative mechanisms to mitigate institutional collective action dilemmas. New York: Cambridge University Press; 2009.

[57] Kapucu N. Interorganizational coordination in complex environments of disasters: the evolution of intergovernmental disaster response systems. J Homel Secur Emerg Manag. 2009;6(1):1–26.

[58] McPherson M, Smith-Lovin L, Cook JM. Birds of a feather: homophily in social networks. Annu Rev Sociol. 2001;27(1):415–44.

[59] Farrell H. The consequences of the internet for politics. Annu Rev Polit Sci. 2012;15(1):35–52.

[60] Di Gregorio M. Networking in environmental movement organisation coalitions: interest, values or discourse? Env Polit. 2012;21(1):1–25.

[61] Song M, Park HJ, Jung K. Do political similarities facilitate interlocal collaboration? Public Adm Rev. 2018;78(2):261–9.

[62] Yang A, Taylor M. Looking over, looking out, and moving forward: positioning public relations in theorizing organizational network ecologies. Commun Theory. 2015;25(1):91–115.

[63] Re Swiss. Natural catastrophes and man-made disasters in 2017: a year of record-breaking losses [Internet]. Zurich: Swiss Re Management Ltd; 2018 [cited 2019 Sep 18]. Available from: https://www.swissre.com/dam/jcr:1b3e94c3-ac4e-4585-aa6f-4d482d8f46cc/sigma1_2018_en.pdf.

[64] Yap C, Sullivan BK., Calonzo A. Hong Kong on lockdown as Typhoon Mangkhut arrives. Bloomberg. 2018 Sep 15 [cited 2019 Aug 22]. Available from: https://www.bloomberg.com/news/articles/2018-09-14/super-typhoon-mangkhut-slams-philippines-with-category-5-power.

[65] Whelan E, Teigland R, Vaast E, Butler B. Expanding the horizons of digital social networks: mixing big trace datasets with qualitative approaches. Inf Organ. 2016;26(1–2):1–12.

[66] Galaskiewicz J, Wasserman S. A dynamic study of change in a regional corporate network. Am Sociol Rev. 1981;46(4):475–84.

[67] Newman ME. The structure and function of complex networks. SIAM Rev Soc Ind Appl Math. 2003;45(2):167–256.

[68] Yi H, Scholz JT. Policy networks in complex governance subsystems: observing and comparing hyperlink, media, and partnership networks. Policy Stud J. 2016;44(3):248–79.

[69] Andrew SA. Adaptive versus restrictive contracts: can they resolve different risk problems. In: Feiock R, Scholz JT, editors. Self-organizing federalism: collaborative mechanisms to mitigate institutional collective action dilemmas. New York: Cambridge University Press; 2010. p. 91–113.

[70] Berardo R, Scholz JT. Self‐organizing policy networks: risk, partner selection, and cooperation in estuaries. Am J Pol Sci. 2010;54(3):632–49.

[71] Lai CH, She B, Ye X. Unpacking the network processes and outcomes of online and offline humanitarian collaboration. Commun Res. 2019;46(1):88–116.

[72] Hallahan K, Holtzhausen D, Van Ruler B, Verčič D, Sriramesh K. Defining strategic communication. Int J Strateg Commun. 2007;1(1):3–35.

[73] Sun J. The multiplex networks of strategic alliances and follower–followee relations among US technology companies. Int J Commun. 2020;14:4096–116.

[74] Koliba CJ, Meek JW, Zia A, Mills RW. Governance networks in public administration and public policy. New York: Routledge; 2017.

[75] Borgatti SP, Everett MG, Freeman LC. UCINET for Windows: software for social network analysis. Harvard: Analytic Technologies; 2002.

[76] Hanneman RA, Riddle M. Introduction to social network methods. Riverside: University of California, Riverside; 2005.

[77] Dekker D, Krackhardt D, Snijders TA. Sensitivity of MRQAP tests to collinearity and autocorrelation conditions. Psychometrika. 2007;72(4):563–81. doi: 10.1007/s11336-007-9016-1 20084106 PMC2798974

[78] Gidron B, Kramer RM, Salamon LM. Government and the third sector: emerging relationships in welfare states. San Francisco: Jossey-Bass; 1992.

[79] Hawkins CV, Hu Q, Feiock RC. Self‐organizing governance of local economic development: informal policy networks and regional institutions. J Urban Aff. 2016;38(5):643–60.

[80] Choi SO, Brower RS. When practice matters more than government plans: a network analysis of local emergency management. Adm Soc. 2006;37(6):651–78.

[81] Kapucu N, Augustin ME, Garayev V. Interstate partnerships in emergency management: emergency management assistance compact in response to catastrophic disasters. Public Adm Rev. 2009;69(2):297–313.

[82] Sherraden MS, Fox E. The Great Flood of 1993: response and recovery in five communities. J Community Pract. 1997;4(3):23–45.

[83] Drabek TE, McEntire DA. Emergent phenomena and the sociology of disaster: lessons, trends and opportunities from the research literature. Disaster Prev Manag. 2002;12(2):97–112.

[84] Grimmelikhuijsen S, Jilke S, Olsen AL, Tummers L. Behavioral public administration: combining insights from public administration and psychology. Public Adm Rev. 2017;77(1):45–56.

[85] Nowell B, Steelman T. Communication under fire: the role of embeddedness in the emergence and efficacy of disaster response communication networks. J Public Adm Res Theory. 2015;25(3):929–52.

[86] Pleuss JD, Stamm JLH, Ellis JD. Using simulated annealing to improve the information dissemination network structure of a foreign animal disease outbreak response. J Homel Secur Emerg Manag. 2018;15(3):20170018.

