# Supplementary material for: Longitudinal multiplexity and structural constraints of online emergency collaborative networks: A tale of two Chinese societies
Source: PLoS One. 2023 Jul 27;18(7):e0289277. doi: 10.1371/journal.pone.0289277 (PMC10374111; doi:10.1371/journal.pone.0289277)
Supplement: S1 Table — (DOCX) [file pone.0289277.s001.docx]

**Government documents (situation reports and after-action reports) identified from the government news website of Shenzhen**

| No | Information source | Date of publication | Report title |
| --- | --- | --- | --- |
| 1 | 深圳市住房和建設局 | 2018-09-12 | 深圳市住房和建設局關於做好全市住建系統“百里嘉”、“山竹”颱風防禦工作的緊急通知 |
| 2 | 羅湖區 | 2018-09-12 | 羅湖區黃貝街道召開颱風“百里嘉”、“山竹”防禦工作部署會 |
| 3 | 南山區 | 2018-09-12 | 南山區住房和建設局關於轉發《深圳市住房和建設局關於做好全市住建系統“百里嘉”、“山竹”颱風防禦工作緊急通知》的通知 |
| 4 | 大鵬新區 | 2018-09-13 | 大鵬新區機關事務管理局召開颱風“百里嘉”“山竹”防禦工作會議 |
| 5 | 深圳市規劃和國土資源委員會（市海洋局） | 2018-09-13 | 市規劃國土委光明管理局召開防禦“百里嘉”、“山竹”雙颱風緊急工作部署會議 |
| 6 | 深圳市民政局 | 2018-09-13 | 市殯葬管理所緊急召開颱風“山竹”防禦準備工作會議 |
| 7 | 羅湖區 | 2018-09-13 | 羅湖區社會福利中心召開防禦“百里嘉”“山竹”雙颱風暨安全生產工作部署會 |
| 8 | 光明新區 | 2018-09-13 | 光明新區環境保護和水務局關於切實做好“百里嘉”、“山竹”颱風防禦及中秋、國慶假期期間水務安全生產管理工作的通知 |
| 9 | 光明新區 | 2018-09-13 | 光明新區住房和建設局關於做好 “百里嘉”、“山竹”颱風防禦工作的緊急通知 |
| 10 | 鹽田區 | 2018-09-13 | 鹽田街道明珠社區工作站召開防禦颱風“百里嘉”“山竹”工作部署會 |
| 11 | 寶安區 | 2018-09-13 | 查隱患備物資轉人員 寶安全力以赴防禦“山竹” |
| 12 | 光明新區 | 2018-09-13 | 光明新區安監局關於轉發《市安全監管局關於做好颱風“百里嘉”“山竹”防禦工作的緊急通知》 |
| 13 | 深圳市城市管理局 | 2018-09-14 | 市城管局馮增軍副局長率隊到地質公園開展颱風“山竹”防禦工作檢查 |
| 14 | 寶安區 | 2018-09-14 | 超強颱風“山竹”正直奔廣東 寶安全面進入戰時狀態 |
| 15 | 深圳市住房和建設局 | 2018-09-14 | 深圳市住房和建設局關於進一步強化在建工程起重機械和腳手架防禦超強颱風“山竹”管理的緊急通知 |
| 16 | 深圳市氣象局 | 2018-09-14 | 9月14日：超強颱風“山竹”將給我市帶來狂風暴雨 |
| 17 | 深圳市住房和建設局 | 2018-09-14 | 深圳市住房和建設局進一步強化在建工程防禦超強颱風“山竹”工作的緊急通知 |
| 18 | 深圳市規劃和國土資源委員會（市海洋局） | 2018-09-14 | 市規劃國土委坪山管理局全力應對颱風“山竹” |
| 19 | 市前海管理局 | 2018-09-14 | 前海控股公司提前部署 嚴密防範強颱風“山竹” |
| 20 | 深圳市城市管理局 | 2018-09-14 | 深圳市公園管理中心召開超強颱風“山竹”防禦工作專題會議 |
| 21 | 深圳市城市管理局 | 2018-09-14 | 深圳市公園管理中心關於超強颱風“山竹”的溫馨提示 |
| 22 | 深圳市氣象局 | 2018-09-14 | 深圳市氣象局三級回應迎戰颱風“山竹” |
| 23 | 深圳市城市管理局 | 2018-09-14 | 市城管局地質公園全力做好颱風“山竹”防禦工作 |
| 24 | 光明新區 | 2018-09-15 | 光明新區環境保護和水務局關於切實做好超強颱風“山竹”登陸期間環境安全保障工作的緊急通知 |
| 25 | 市水務局 | 2018-09-15 | 全市防禦第22號颱風“山竹”視訊會議召開，王偉中強調 嚴陣以待 嚴防死守 全力以赴打好颱風防禦戰 |
| 26 | 深圳市城市管理局 | 2018-09-15 | 市城管局制定方案防禦超強颱風“山竹” |
| 27 | 深圳市城市管理局 | 2018-09-15 | 市城管局防禦“山竹”在行動 王國賓局長率隊檢查颱風防禦準備工作 |
| 28 | 深圳市氣象局 | 2018-09-15 | 颱風“山竹”來勢洶洶，深圳市氣象局全力應戰 |
| 29 | 深圳市民政局 | 2018-09-16 | 深圳市民政局全力以赴做好颱風“山竹”防禦和救災應急工作 |
| 30 | 市水務局 | 2018-09-16 | 市水務局加強部署防禦颱風“山竹” |
| 31 | 市水務局 | 2018-09-16 | 全市上下嚴陣以待防禦颱風“山竹” |
| 32 | 深圳市城市管理局 | 2018-09-16 | 市城管局召開抗擊颱風“山竹”指揮調度會議 |
| 33 | 深圳市城市管理局 | 2018-09-16 | 市城管局公園管理中心梅村主任帶隊到深圳灣公園現場靠前指揮抗擊“山竹”超強颱風 |
| 34 | 深圳市城市管理局 | 2018-09-16 | 市城管局綠化管理處全力以赴應戰超強颱風“山竹” |
| 35 | 深圳市氣象局 | 2018-09-16 | “四停與我們無關” 深圳氣象人厲兵秣馬迎戰颱風“山竹” |
| 36 | 市前海管理局 | 2018-09-16 | 前海嚴密部署颱風“山竹”防禦工作 最高規格嚴防死守確保安全 |
| 37 | 深圳市民政局 | 2018-09-16 | 颱風“山竹”襲深，社會組織在行動 |
| 38 | 深圳特區報 | 2018-09-17 | 市領導督導防禦颱風“山竹”工作 |
| 39 | 深圳市民政局 | 2018-09-17 | 市軍休二所開展颱風“山竹”災後清理修復工作 |
| 40 | 深圳市住房和建設局 | 2018-09-17 | 深圳市住房和建設局關於建築工地組織開展颱風“山竹”復工檢查的通知 |
| 41 | 寶安區 | 2018-09-17 | 強颱風“山竹”昨來襲，寶安全區上下不舍晝夜全面迎戰 |
| 42 | 寶安區 | 2018-09-17 | 寶安交通運輸局全力抵禦強颱風“山竹”，已處理倒伏路樹17處、道路積水17處 |
| 43 | 羅湖區 | 2018-09-17 | 羅湖區社會福利中心堅守崗位做好颱風“山竹”期間安全生產工作 |
| 44 | 羅湖區 | 2018-09-17 | 山竹無情東湖有情，全力以赴為居民——羅湖區東湖街道全員出動打通生命通道全力救治傷患 |
| 45 | 坪山區 | 2018-09-17 | 坪山區規劃土地監察局積極做好颱風“山竹”防禦工作之部署篇 |
| 46 | 深圳市規劃和國土資源委員會（市海洋局） | 2018-09-17 | 深圳海監漁政支隊全力以赴抗擊颱風“山竹” |
| 47 | 深圳市城市管理局 | 2018-09-17 | 市城管局地質公園全力開展颱風“山竹”災後恢復工作 |
| 48 | 南山區 | 2018-09-17 | 南山區住房和建設局關於建築工地組織開展颱風“山竹”復工檢查的通知 |
| 49 | 深圳市城市管理局 | 2018-09-17 | 市城管局城市廢物處置中心全力防禦颱風“山竹” |
| 50 | 深圳市氣象局 | 2018-09-17 | 9月17日：“山竹”影響已結束 未來十天無颱風影響我市 |
| 51 | 深圳市民政局 | 2018-09-17 | 市民政局皮勇華副局長到市救助管理站檢查指導“山竹”颱風防禦工作 |
| 52 | 深圳市城市管理局 | 2018-09-17 | 市城管局綠化管理處研究部署颱風“山竹”清理善後工作 |
| 53 | 深圳市城市管理局 | 2018-09-17 | 市城管局綠化管理處全力以赴應戰超強颱風“山竹” |
| 54 | 深圳市民政局 | 2018-09-17 | 颱風“山竹”強勢來襲，救助前沿嚴陣以待 |
| 55 | 市水務局 | 2018-09-17 | 市治河辦堅守一線迎戰颱風“山竹” |
| 56 | 市水務局 | 2018-09-17 | 市鐵石管理處全力防禦颱風“山竹”確保水庫安全 |
| 57 | 深圳市城市管理局 | 2018-09-17 | 深圳市城市管理局關於颱風“山竹”災後道路清理恢復的溫馨提示 |
| 58 | 寶安區 | 2018-09-18 | 寶安城管人戰“山竹”獲群眾點贊 |
| 59 | 大鵬新區 | 2018-09-18 | 大鵬新區大鵬交通局“五到位”應對“山竹” 颱風防禦工作 |
| 60 | 大鵬新區 | 2018-09-18 | 大鵬管理局周密部署堅決防禦颱風“山竹” |
| 61 | 深圳特區報 | 2018-09-18 | 深圳召開全市防禦颱風“山竹”情況視頻工作會議 |
| 62 | 羅湖區 | 2018-09-18 | 羅湖區翠竹街道木頭龍社區用行動抵禦颱風“山竹” |
| 63 | 羅湖區 | 2018-09-18 | 羅湖區翠竹街道總工會積極分子參加防禦“山竹”臨時救助點後勤工作 |
| 64 | 深圳市民政局 | 2018-09-18 | 市民政局副巡視員張啟榮到市軍供站等單位檢查颱風“山竹”受災情況 |
| 65 | 寶安區 | 2018-09-18 | 抗擊颱風“山竹” 寶安區人武部出動600餘民兵參與搶險 |
| 66 | 龍華區 | 2018-09-18 | 龍華區民政局九個科室全體幹部職工奮戰在颱風“山竹”防禦救助現場 |
| 67 | 寶安區 | 2018-09-18 | 寶安全力推進防禦颱風“山竹”應急疏散轉移工作 |
| 68 | 寶安區 | 2018-09-18 | 寶安排水公司防禦強颱風“山竹”紀實 |
| 69 | 深圳市規劃和國土資源委員會（市海洋局） | 2018-09-18 | 市規劃國土委羅湖管理局積極落實雙颱風“百里嘉”“山竹”各項防禦工作 |
| 70 | 龍華區 | 2018-09-18 | 龍華區收看收聽全省防禦颱風“山竹”情況回饋會議 |
| 71 | 深圳市城市管理局 | 2018-09-18 | 颱風“山竹”肆虐鵬城，市城管局森林消防奮戰一線 |
| 72 | 深圳市城市管理局 | 2018-09-18 | 市城管局垃圾處理監管中心積極部署颱風“山竹”災後恢復工作 |
| 73 | 市水務局 | 2018-09-18 | 市西麗水庫管理處Ⅰ級回應全力應對颱風“山竹”保安全 |
| 74 | 市水務局 | 2018-09-18 | 深圳市召開全市防禦颱風“山竹”情況視頻工作會議部署下一步防風防汛和災後恢復工作 |
| 75 | 福田區 | 2018-09-18 | 嚴防颱風“山竹” 福田民兵勇擔當 |
| 76 | 深圳市城市管理局 | 2018-09-18 | 市城管局公園管理中心胡振華副主任帶隊親臨公園指導颱風“山竹”災後搶險工作 |
| 77 | 寶安區 | 2018-09-18 | 寶安區城市更新局扎實開展更新專案防颱風“山竹”安全專項檢查 |
| 78 | 光明新區 | 2018-09-18 | 光明新區住房和建設局轉發深圳市住房和建設局關於建築工地組織開展颱風“山竹”復工檢查的通知 |
| 79 | 深圳市城市管理局 | 2018-09-18 | 市城管局公園管理中心謝良生副主任帶隊親臨公園指導颱風“山竹”災後搶險工作 |
| 80 | 鹽田區 | 2018-09-19 | 颱風“山竹”重創鹽田旅遊景點 大小梅沙中秋國慶閉園 |
| 81 | 鹽田區 | 2018-09-19 | 舉全區之力抵禦颱風“山竹” 鹽田築牢防汛防風安全堡壘 |
| 82 | 深圳市城市管理局 | 2018-09-19 | 市城管局張國宏副局長到市綠化處督導颱風“山竹”善後清理工作 |
| 83 | 光明新區 | 2018-09-19 | 市城管局綠化管理處張成堯副書記帶隊巡查深南大道西段 督導颱風“山竹”善後清理工作 |
| 84 | 深圳市城市管理局 | 2018-09-19 | 市城管局綠化處行走深南大道東段 督導颱風“山竹”善後清理工作 |
| 85 | 深圳市城市管理局 | 2018-09-19 | 市城管局監察支隊連夜支援深圳灣公園颱風“山竹”善後清理工作 |
| 86 | 深圳市城市管理局 | 2018-09-19 | 市城管局鐘勇文處長率隊到塘朗山郊野公園進行颱風“山竹”過境後災害情況調研及安全生產檢查 |
| 87 | 深圳市城市管理局 | 2018-09-19 | 深圳市城市管理局關於颱風“山竹”災後注意避讓受損樹木和邊坡的溫馨提示 |
| 88 | 羅湖區 | 2018-09-19 | 羅湖區黃貝U站義工助力颱風“山竹”善後道路清理工作 |
| 89 | 深圳市規劃和國土資源委員會（市海洋局） | 2018-09-19 | 市規劃國土委福田管理局積極落實颱風“山竹”期間各項防禦工作 |
| 90 | 深圳市人民政府口岸辦公室 | 2018-09-20 | 寶安海事局組織召開防抗颱風“山竹”工作評估會 |
| 91 | 市口岸辦 | 2018-09-20 | 颱風“山竹”過境後，深圳邊檢總站認真做好口岸客流疏導工作 |
| 92 | 市口岸辦 | 2018-09-20 | 寶安海事局組織召開防抗颱風“山竹”工作評估會 |
| 93 | 深圳市衛生和計劃生育委員會 | 2018-09-20 | 平凡的工作 非凡的功績——防禦超級風王山竹，職防人在行動！ |
| 94 | 坪山區 | 2018-09-20 | 坪山區石井街道辦事處召開防禦颱風“山竹”工作總結會議 |
| 95 | 羅湖區 | 2018-09-20 | 羅湖區“掃除‘山竹’、美麗羅湖” 市容環境大掃除活動倡議書 |
| 96 | 鹽田區 | 2018-09-21 | 鹽田區政協領導中秋節前走訪慰問優撫對象暨視察颱風“山竹”過後海鮮街受災情況 |
| 97 | 大鵬新區 | 2018-09-21 | 大鵬新區城市建設局開展颱風“山竹”過後建築工地復工前安全檢查工作 |
| 98 | 大鵬新區 | 2018-09-21 | 大鵬新區領導帶隊檢查強颱風“山竹”過境後恢復工作 |
| 99 | 大鵬新區 | 2018-09-21 | “山竹”過後 大鵬新區城市更新局黨員志願者在行動 |
| 100 | 鹽田區 | 2018-09-21 | 省防總秘書長賀國慶一行在鹽田街道調研颱風“山竹”災情 |
| 101 | 鹽田區 | 2018-09-21 | 鹽田街道老年人日間照料中心開展“山竹無情人間有愛”社區探訪活動 |
| 102 | 羅湖區 | 2018-09-21 | 羅湖區數字政府建設管理局積極參與“掃除‘山竹’美麗羅湖”大清掃活動 |
| 103 | 羅湖區 | 2018-09-21 | 羅湖區安監局：掃除“山竹”，清潔羅湖，安監人在行動 |
| 104 | 福田區 | 2018-09-21 | “山竹”過後，福田員警做起了這些事…… |
| 105 | 深圳市民政局 | 2018-09-21 | 眾志成城抵禦風王，精心服務保障新兵運輸——市軍供站積極防禦颱風“山竹”，全力保障新兵運輸 |
| 106 | 鹽田區 | 2018-09-25 | “山竹”過境鹽田國際碼頭開閘 聯勤聯動確保交通疏導高效運轉 |
| 107 | 羅湖區 | 2018-09-25 | 羅湖區工商聯積極參與“掃除‘山竹’、美麗羅湖”大清掃活動 |
| 108 | 寶安區 | 2018-09-25 | 寶安召開防禦颱風“山竹”工作總結會 |
| 109 | 福田區 | 2018-09-25 | 福田區華強北街道全面完成“山竹”災後清障任務 |
| 110 | 深圳市衛生和計劃生育委員會 | 2018-09-25 | 市衛生監督局積極投入颱風“山竹”災後飲用水重點衛生監督工作 |
| 111 | 深圳市城市管理局 | 2018-09-26 | 市城管局張國宏副局長帶隊到地質公園指導颱風“山竹”災後清理恢復暨開展黨日活動 |
| 112 | 鹽田區 | 2018-09-29 | 鹽田區沙頭角街道召開防禦應對“山竹”颱風災害總結表彰大會 |
| 113 | 深圳市城市管理局 | 2018-09-30 | 市城管局深圳灣公園召開“山竹”清障工作會 |

**Government documents (situation reports and after-action reports) identified from the government news website of Hong Kong**

| No | Information source | Date of publication | Report title |
| --- | --- | --- | --- |
| 1 | 治安 | 2018-09-12 | 跨部門會議應對超強颱風 |
| 2 | 社區與健康 | 2018-09-13 | 民政總署加強颱風應變 |
| 3 | 社區與健康 | 2018-09-14 | 嚴陣以待 應對颱風襲港 |
| 4 | 社區與健康 | 2018-09-14 | 超強颱風逼近 應變措施加強 |
| 5 | 社區與健康 | 2018-09-15 | 大澳臨時庇護中心開放 |
| 6 | 社區與健康 | 2018-09-15 | 應對超強颱風 警隊準備充足 |
| 7 | 社區與健康 | 2018-09-15 | 高度戒備 減低風暴影響 |
| 8 | 社區與健康 | 2018-09-15 | 民安隊助低窪地區居民防風 |
| 9 | 社區與健康 | 2018-09-15 | 緊急監援中心啟動 應付颱風威脅 |
| 10 | 財經 | 2018-09-16 | 陳茂波網誌：保險機遇 |
| 11 | 教育與就業 | 2018-09-16 | 教育局宣布明日停課 |
| 12 | 行政與公民事務 | 2018-09-16 | 颱風襲港 特首感謝緊守崗位人員 |
| 13 | 行政與公民事務 | 2018-09-17 | 全力善後 回復本港正常運作 |
| 14 | 社區與健康 | 2018-09-17 | 颱風破壞廣泛 部門加緊善後 |
| 15 | 教育與就業 | 2018-09-17 | 教育局宣布明日繼續停課 |
| 16 | 教育與就業 | 2018-09-17 | 致力盡早恢復全線巴士服務 |
| 17 | 行政與公民事務 | 2018-09-18 | 全面檢視颱風應對措施 |
| 18 | 治安 | 2018-09-18 | 冀10月開會檢討天災應變計劃 |
| 19 | 社區與健康 | 2018-09-18 | 應對登革熱會議跟進防蚊工作 |
| 20 | 社區與健康 | 2018-09-18 | 民商官合作 推動社會共融 |
| 21 | 社區與健康 | 2018-09-19 | 張建宗：研究改善颱風應變措施 |
| 22 | 教育與就業 | 2018-09-19 | 楊潤雄視察受損學校復修進度 |
| 23 | 社區與健康 | 2018-09-19 | 劉江華視察颱風善後工作 |
| 24 | 教育與就業 | 2018-09-19 | 張建宗籲體諒僱員風後上班情況 |
| 25 | 環境 | 2018-09-20 | 渠務署搶修受損排污設施 |
| 26 | 社區與健康 | 2018-09-23 | 三百佳燈 燃亮漁村文化 |
| 27 | 社區與健康 | 2018-09-21 | 各部門繼續跟進颱風善後工作 |
| 28 | 基建與物流 | 2018-09-22 | 調配人手 盡快清理塌樹 |
| 29 | 教育與就業 | 2018-09-23 | 探討改善制度 應對未來天災 |
| 30 | 環境 | 2018-09-24 | 黃錦星視察臨時木料廢物收集處 |
| 31 | 行政與公民事務 | 2018-09-24 | 林鄭月娥西貢視察颱風善後情況 |
| 32 | 行政與公民事務 | 2018-09-25 | 張建宗視察受颱風影響康樂設施 |
| 33 | 行政與公民事務 | 2018-09-26 | 聶德權訪南區特殊學校 |
| 34 | 教育與就業 | 2018-09-27 | 教局為學校提供風災津貼 |
| 35 | 社區與健康 | 2018-09-27 | 黃錦星視察郊野公園清理進度 |
| 36 | 環境 | 2018-09-28 | 山竹之後 齊心善後 |
| 37 | 社區與健康 | 2018-09-28 | 黃偉綸視察西貢發展 |
| 38 | 行政與公民事務 | 2018-09-28 | 特首：巿面恢復正常運作 |
| 39 | 行政與公民事務 | 2018-09-29 | 推新措施必平衡勞資利益 |
| 40 | 基建與物流 | 2018-09-30 | 推試點計劃 更替老化台灣相思 |
| 41 | 環境 | 2018-10-03 | 山竹襲港 十號信號歷時次高 |
| 42 | 社區與健康 | 2018-10-04 | 張建宗：跨部門檢討風災措施 |
| 43 | 社區與健康 | 2018-10-06 | 140名民安隊員協助風災善後 |
| 44 | 社區與健康 | 2018-10-18 | 9月誘蚊產卵器指數降 |
| 45 | 社區與健康 | 2018-10-21 | 張建宗：颱風善後從未間斷 |
| 46 | 行政與公民事務 | 2018-10-23 | 百名優秀公務員獲嘉許 |
