# Supplementary material for: Longitudinal multiplexity and structural constraints of online emergency collaborative networks: A tale of two Chinese societies
Source: PLoS One. 2023 Jul 27;18(7):e0289277. doi: 10.1371/journal.pone.0289277 (PMC10374111; doi:10.1371/journal.pone.0289277)
Supplement: S2 Table — (DOCX) [file pone.0289277.s002.docx]

**Newspaper articles identified from Shenzhen-based newspapers**

| No | Newspaper | Date of publication | Layout | News title |
| --- | --- | --- | --- | --- |
| 1 | SMD-SZ | 2018-09-10 | AII02 | 台風“山竹”上路 等它的一周雨雨雨 |
| 2 | SMD-SZ | 2018-09-11 | AII07 | “山竹”與“23號”將到 明後日大暴雨 第23號颱風週三掠過，“山竹”16-17日嚴重影響廣東 |
| 3 | SMD-SZ | 2018-09-12 | AII02 | 受颱風“山竹”影響 廣深港等多趟列車昨起停售或調整 |
| 4 | SMD-SZ | 2018-09-18 | AII10 | 逆風而行、冒雨前進，全力保障全市安全穩定供氣 深圳燃氣多措並舉 抗擊颱風“山竹” |
| 5 | SMD-SZ | 2018-09-18 | AII02, AII03 | 深圳遭遇“山竹”風卷雲湧的72小時 |
| 6 | SMD-SZ | 2018-09-18 | AII14 | 受理超強颱風報案逾3400宗，與千萬深圳人心手相連 深圳人保財險衆志成城迎風而上，抗擊916“山竹” |
| 7 | SMD-SZ | 2018-09-19 | AII01 | “山竹”重創東部 旅行社忙著改綫路 |
| 8 | SMD-SZ | 2018-09-20 | LG08 | “山竹”來襲奮戰一綫 龍崗街道全員勇當“逆風者” 黨組織成為搶險備戰的主心骨 |
| 9 | SMD-SZ | 2018-09-20 | FT01 | “山竹”來勢洶洶 福田沉著“應戰” |
| 10 | SMD-SZ | 2018-09-20 | AII08 | “山竹”過後，大鵬“紅馬甲”街清障 |
| 11 | SMD-SZ | 2018-09-20 | FT02 | 福田全力以赴防禦“山竹” 衆志成城恢複家園 |
| 12 | SMD-SZ | 2018-09-20 | FT03 | 致敬！“山竹”襲來，感恩這些最可愛的人在身邊 |
| 13 | SMD-SZ | 2018-09-20 | LG06 | 強台風“山竹”肆虐，為了守護阪田安全，他們在狂風暴雨中堅守 搶險救災者成最堅定的“風暴逆行者” |
| 14 | SMD-SZ | 2018-09-20 | LG02 | 防禦“山竹”，他們是最帥的逆行者 龍崗颱風期間出動近3萬人，轉移了近9萬人 |
| 15 | SMD-SZ | 2018-09-20 | GM06 | 水務抵禦颱風“山竹” 保障安全優質供水 |
| 16 | SMD-SZ | 2018-09-20 | GM03 | 提前數日部署防禦，全面排查隱患、開展人員轉移，嚴陣以待加強值守 “山竹”登陸前後72小時 全區戒備無人傷亡 |
| 17 | SMD-SZ | 2018-09-21 | LHU01 | 掃除“山竹”重整家園 |
| 18 | SMD-SZ | 2018-09-21 | PS04 | “山竹”過境72小時“逆風行者”守護坪山 因防禦得當，坪山在此次風王肆虐的過程中，無一人死傷 |
| 19 | SMD-SZ | 2018-09-21 | LHU08 | 風王“山竹”襲深，誰是最可愛的人？ |
| 20 | SMD-SZ | 2018-09-21 | NS06 | “山竹”期間的感人瞬間（之二）賦予榕樹新生 給學生上一堂生命教育課 |
| 21 | SMD-SZ | 2018-09-21 | LH02 | 颱風“山竹”過後 全面開展清障行動 |
| 22 | SMD-SZ | 2018-09-21 | BA02 | 寶安抵禦“山竹”的72小時 |
| 23 | SMD-SZ | 2018-09-21 | LHU04 | “山竹”過境，羅湖城管第一時間奔赴一線搶險救災 連續奮戰30小時，52歲綠化能手因疲憊不慎被鋸傷 |
| 24 | SMD-SZ | 2018-09-21 | NS02 | 全民防禦“山竹” 凝心聚力傳遞南山大愛 |
| 25 | SMD-SZ | 2018-09-21 | PS02 | “山竹”過境 坪山穩住了坪山區以“戰備狀態”防禦“山竹”，保障居民生命財產安全 |
| 26 | SMD-SZ | 2018-09-28 | AII04 | “山竹”過境布心花園有驚無險 業主期盼舊改早住新房 |
| 27 | SEN | 2018-09-11 | A02 | 本周將有兩個颱風影響深圳（熱帶低壓將加強為今年第23號颱風） |
| 28 | SEN | 2018-09-12 | A02 | “山竹”“百里嘉”雙雙來襲（部分線路列車停止售票，市三防指揮部對防颱風工作作出部署） |
| 29 | SEN | 2018-09-13 | A03 | 颱風“山竹”或給深圳帶來嚴重風雨影響（我市今起風雨減弱，“山竹”16日、17日將正面襲擊廣東） |
| 30 | SEN | 2018-09-14 | A04 | 颱風“山竹”或將嚴重影響深圳（預計16日至17日深圳沿海和高地最大陣風可達12級） |
| 31 | SEN | 2018-09-15 | A02 | 深圳明日或風急雨驟（降水從今日下半夜開始，17日白天降水減弱） |
| 32 | SEN | 2018-09-15 | A02 | 嚴陣以待 嚴防死守 全力以赴打好颱風防禦戰（全市防禦第22號颱風“山竹”視訊會議召開，王偉中強調） |
| 33 | SEN | 2018-09-15 | A02 | 400余處應急避難場所隨時準備開放 |
| 34 | SEN | 2018-09-15 | A02 | 開展地毯式排查 確保隱患點無遺漏（市規劃國土委） |
| 35 | SEN | 2018-09-16 | A01 | 一級（市氣象臺發佈颱風紅色預警 全市防颱風和防汛I級應急回應啟動） |
| 36 | SEN | 2018-09-16 | A02 | 堅決打好打贏超強颱風“山竹”防禦戰（深圳召開全市動員會貫徹落實全省防禦颱風戰前動員會精神 王偉中出席會議並講話 陳如桂主持） |
| 37 | SEN | 2018-09-16 | A03 | 大鵬新區 571艘在冊漁船全部回港 258名漁排人員全部上岸 |
| 38 | SEN | 2018-09-16 | A03 | 西鄉街道 開展多項防颱風工作 |
| 39 | SEN | 2018-09-16 | A03 | 坪山區 進入全力防禦颱風狀態 |
| 40 | SEN | 2018-09-16 | A03 | 市城管局 制訂方案防禦超強颱風“山竹” |
| 41 | SEN | 2018-09-16 | A04 | 全省學校幼稚園停課停學（9月15日18時起至9月17日，全省教育系統防禦颱風I級應急機制啟動） |
| 42 | SEN | 2018-09-16 | A04 | 今日深圳機場航班、省內高鐵全線停運（地鐵高架區段及車站停運） |
| 43 | SEN | 2018-09-16 | A04 | 深圳供電局 全力部署防禦颱風 |
| 44 | SEN | 2018-09-16 | A04 | 全市1745個在建工地停工 |
| 45 | SEN | 2018-09-17 | A01 | 逆風而行（受颱風“山竹”影響，深圳昨日狂風暴雨 各部門積極行動全力以赴奮戰在搶險救災一線 全市尚未收到重大災情和人員因災死亡報告） |
| 46 | SEN | 2018-09-17 | A02 | 深圳市三防指揮部通告 |
| 47 | SEN | 2018-09-17 | A02 | 王偉中陳如桂督導防禦颱風“山竹”工作 |
| 48 | SEN | 2018-09-17 | A02 | 今日白天仍有中到大雨 午後降雨減弱（“山竹”成30多年以來影響深圳最強颱風） |
| 49 | SEN | 2018-09-17 | A02 | 未收到重大災情和人員因災死亡報告（全市中小學、幼稚園今日停課一天） |
| 50 | SEN | 2018-09-17 | A03 | 惠州載73人工程船走錨漂移至大鵬（深圳接報後緊急馳援） |
| 51 | SEN | 2018-09-17 | A03 | 應急避難所為13.8萬人遮風擋雨（食品足量供應，救災物資倉庫24小時值班值守） |
| 52 | SEN | 2018-09-17 | A04 | 深圳警方轉移安置群眾近11萬人（解救被困人員97人，救助群眾2.2萬餘人） |
| 53 | SEN | 2018-09-17 | A04 | 深圳交警全力保障道路交通安全（車主因避險造成交通違法行為經核實後不予處罰） |
| 54 | SEN | 2018-09-17 | A04 | 深圳燃氣今日恢復供氣 |
| 55 | SEN | 2018-09-17 | A04 | 南方電網深圳供電局連夜搶修 |
| 56 | SEN | 2018-09-17 | A04 | 深圳警備區啟動抗災救災緊急預案 |
| 57 | SEN | 2018-09-17 | A04 | 深圳市公安消防支隊共解救被困人員119名 |
| 58 | SEN | 2018-09-17 | A04 | 深圳在建工程轉移超15萬人 |
| 59 | SEN | 2018-09-17 | A04 | 深晚全媒體平臺48小時總閱讀量超6000萬（本報抖音視頻觀看量達4700萬人次） |
| 60 | SEN | 2018-09-17 | A05 | 兩萬余人連夜清理路障（確保今日6時深南、北環、濱河三條主幹道恢復暢通） |
| 61 | SEN | 2018-09-17 | A06 | 深圳各區（新區）防禦颱風平穩有序 |
| 62 | SEN | 2018-09-18 | A02 | 再接再厲 毫不鬆懈 連續作戰 努力奪取颱風“山竹” 防禦戰全面勝利（深圳召開全市防禦颱風“山竹”情況視頻工作會議，王偉中強調） |
| 63 | SEN | 2018-09-18 | A02 | 抗擊颱風“山竹” 見證深圳力量 |
| 64 | SEN | 2018-09-18 | A03 | 眾志成城 這場大考深圳成功應對 |
| 65 | SEN | 2018-09-18 | A04 | 逆風而行 那些平凡身影感動你我 |
| 66 | SEN | 2018-09-18 | A07 | 攜手相助 許多美麗故事溫暖人心 |
| 67 | SEN | 2018-09-18 | A12 | 颱風後學校黑板上留下感謝信（寶安區開放各個學校給轄區企業員工避險） |
| 68 | SEN | 2018-09-18 | A12 | 颱風後這封感謝信刷爆朋友圈 |
| 69 | SEN | 2018-09-18 | A12 | 羅湖區千人連夜奮戰打通道路 |
| 70 | SEN | 2018-09-18 | A12 | 前海嚴密部署成功抵禦颱風“山竹” |
| 71 | SEN | 2018-09-18 | B01 | 福田區城管局全面啟動樹木倒伏清理工作 |
| 72 | SEN | 2018-09-18 | B02 | 福田人行動起來！“人人動手 清潔家園”倡議書 |
| 73 | SEN | 2018-09-18 | B02 | 全員投入防風救災第一線（梅林街道） |
| 74 | SEN | 2018-09-18 | B02 | “全、細、快”防範應對 爭分奪秒恢復路面正常通行（沙頭街道） |
| 75 | SEN | 2018-09-18 | B02 | 合理安置 責任到人 保障人民群眾生命安全（福田街道） |
| 76 | SEN | 2018-09-18 | B02 | 嚴防死守上步碼頭確保萬無一失（南園街道） |
| 77 | SEN | 2018-09-18 | B03 | 連續作戰緊急清理路障保交通暢通（華富街道） |
| 78 | SEN | 2018-09-18 | B03 | 開放11個避難場所 安置居民3800余人（蓮花街道） |
| 79 | SEN | 2018-09-18 | B03 | 緊急待命嚴格部署 頂風冒雨排查隱患（香蜜湖街道） |
| 80 | SEN | 2018-09-18 | B03 | 全民皆兵 全力以赴搶險清障（福保街道） |
| 81 | SEN | 2018-09-19 | A02 | 在守望相助中感受城市力量 |
| 82 | SEN | 2018-09-19 | A03 | 大小梅沙中秋國慶暫停開放（鹽田區多個景點颱風中受損嚴重，鹽田至南澳航線也暫停營運） |
| 83 | SEN | 2018-09-19 | A04 | 暖心“外賣單”刷爆朋友圈（鹽田教師何佳瑜為奮戰在抗擊颱風一線的環衛工和員警點“外賣”） |
| 84 | SEN | 2018-09-19 | A04 | 垂葉榕等速生樹種最不經風（城管部門分析樹木倒伏受損原因，計畫3天后恢復整潔有序城市環境） |
| 85 | SEN | 2018-09-19 | A05 | 因台風致交通違法週六前免罰（全市公共交通已恢復運營，市民響應號召紛紛綠色出行） |
| 86 | SEN | 2018-09-19 | A05 | 武警官兵連夜奮戰打通“最後一公里”（市民晚上回家還要“穿越叢林”，一大早就能開車出門啦） |
| 87 | SEN | 2018-09-19 | A06 | “山竹”過境海濤花園有驚無險（業主期盼加快舊改早住新房，儘早告別提心吊膽的日子） |
| 88 | SEN | 2018-09-19 | A07 | 倒伏樹木將如何處置？（或原地重植或粉碎後用於發電） |
| 89 | SEN | 2018-09-19 | A08 | “這是我的家，清掃樹葉是應該的”（深圳市民自發加入颱風後的道路清障隊伍，加速恢復美麗家園） |
| 90 | SEN | 2018-09-20 | A02 | 颱風過後，給孩子上一堂生命教育課 |
| 91 | SEN | 2018-09-20 | A02 | “山竹”離去，地鐵成市民出行首選交通工具 |
| 92 | SEN | 2018-09-20 | A08 | 深圳抗擊“山竹”十個難忘瞬間 |
| 93 | SEN | 2018-09-20 | A18 | 坪山區防控颱風次生災害 |
| 94 | SEN | 2018-09-21 | A07 | 愛心月餅送給一線環衛工人 |
| 95 | SEN | 2018-09-21 | A08 | 中秋假期儘量別去東部景區（深圳部分道路還處於搶修狀態，鹽田大鵬部分景區正在修復中） |
| 96 | SEN | 2018-09-21 | A11 | 深圳城市管理經受住颱風考驗（目前市容已基本恢復，中秋節前有望還市民乾淨有序城市環境） |
| 97 | SEN | 2018-09-21 | A18 | 血庫告急！白衣天使帶頭獻血（目前龍崗區血庫A型緊缺，B型和O型偏少） |
| 98 | SEN | 2018-09-21 | A24 | 太平洋壽險深圳分公司舉辦愛心公益活動 |
| 99 | SEN | 2018-09-21 | A26 | 廣藥白雲山、海王星辰藥房為環衛工人送愛心大禮包 |
| 100 | SEN | 2018-09-25 | A04 | 3天假期132萬余人次游公園（中秋夜市屬公園入園遊客約30萬人次） |
| 101 | SEN | 2018-09-25 | A11 | 小梅沙抓緊開展景點修復工作 |
| 102 | SEN | 2018-09-25 | A12 | 深圳福彩人走進紅樹林“扶樹” |
| 103 | SEN | 2018-09-25 | B01 | 福田區加快推進災後恢復工作（機關工作人員深入一線清障） |
| 104 | SEN | 2018-09-25 | B02 | 眾志成城修復家園感人瞬間多（華富街道） |
| 105 | SEN | 2018-09-25 | B02 | 多方攜手奮力清障恢復美麗家園（沙頭街道） |
| 106 | SEN | 2018-09-25 | B03 | 聯動社區力量全力清障保暢通（香蜜湖街道） |
| 107 | SEN | 2018-09-25 | B03 | 全面完成“山竹”災後清障任務（華強北街道） |
| 108 | SEN | 2018-09-25 | B03 | 砍掉倒伏大樹太浪費？福田區城管局道出其中原委 |
| 109 | SEN | 2018-09-25 | B03 | 福田機關幹部清掃南園 企業自發送飲料月餅 |
| 110 | SEN | 2018-09-26 | A12 | 深圳人保財險助力“掃除‘山竹’、 美麗羅湖”活動 |
| 111 | SEN | 2018-09-26 | A16 | 東部景區仍在修復 交警取消國慶假期預約通行 |
| 112 | SEN | 2018-09-29 | A04 | 颱風吹倒的樹樁被鋸成“砧板”賣？ 清障人員：這只是一個誤會 |
| 113 | SEN | 2018-09-30 | A04 | 警方提示國慶假期出行注意安全 |
| 114 | SEN | 2018-09-30 | A07 | 3933名白衣天使獻血暖人心（深第七屆“白衣天使捐血月”大型無償獻血活動完美收官） |
| 115 | SEN | 2018-10-08 | A07 | 城市管理者服務國慶長假不停歇（市屬公園接待遊客近300萬人次，燈光秀9天接待觀眾112萬人次） |
| 116 | SEN | 2018-10-09 | A09 | 沙頭角換植“大腹木棉” |

**Newspaper articles identified from Hong Kong-based newspapers**

| No | Newspaper | Date of publication | Layout | News title |
| --- | --- | --- | --- | --- |
| 1 | MP | 2018-09-11 | A06 | 熱帶風暴先到 今料掛一號 兩風接踵 山竹或成超強颱風撲港 |
| 2 | MP | 2018-09-12 | A01 | 百里嘉逼近今或3號波 山竹周日一半機率撲港 |
| 3 | MP | 2018-09-13 | A02 | 超強颱風山竹周日晚逼港 210公里風力超天鴿 鯉魚門民居擋水板加高 |
| 4 | MP | 2018-09-13 | A02 | 鯉魚門防波堤加固 商戶抬高電器 居民隨時撤離 |
| 5 | MP | 2018-09-13 | A22 | 4校周末升小面試 遇打風或改期 |
| 6 | MP | 2018-09-14 | A01 | 山竹勢兇恐現風暴潮 跨部會議今再開 政府籲鯉魚門大澳居民撤離 |
| 7 | MP | 2018-09-14 | A01 | 08年風暴潮大澳水浸心口 泵房改善料下年完工 |
| 8 | MP | 2018-09-14 | A01 | 風力逾200公里 港珠澳橋承受力受考驗 |
| 9 | MP | 2018-09-14 | E01 | 麥偉利要響勝鼓 盼周日馬照跑 |
| 10 | MP | 2018-09-14 | A26 | 山竹襲港「數人頭」改下周三 |
| 11 | MP | 2018-09-15 | A01 | 山竹12級颶風 明勢掛8號波 未入800公里1號風球 港鐵西九站設「war room」 |
| 12 | MP | 2018-09-15 | A02 | 官僚心態防災大忌 主動應變勝「等風到」 |
| 13 | MP | 2018-09-15 | A01 | 大澳長者不願走 鄉委會憂棚屋骨牌式吹走 |
| 14 | MP | 2018-09-15 | B02 | 山竹來襲 實名制延期實施 |
| 15 | MP | 2018-09-15 | A01 | 150水浸黑點 渠署續清理堆沙包 |
| 16 | MP | 2018-09-16 | A01 | 全球最強風暴 山竹今午最接近 逾540航班取消 9.6萬旅客受影響 |
| 17 | MP | 2018-09-16 | A02 | 港珠澳橋專家：可抵時速432公里風力 |
| 18 | MP | 2018-09-16 | A02 | 連鎖食肆8號波照開 勞團促增保障 指勞處指引無約束力 籲修例 |
| 19 | MP | 2018-09-16 | A01 | 棚屋長者撤離：不想最後一刻叫人救 |
| 20 | MP | 2018-09-17 | A02 | 百公里外掠過 並列歷來最遠10號波 大埔滘風暴潮最嚴重 |
| 21 | MP | 2018-09-17 | A01 | 山竹風速超溫黛 歷來次高 僅遜愛倫 10號波掛10句鐘 |
| 22 | MP | 2018-09-17 | A11 | 網上指珠三角將大停電 官媒闢謠 |
| 23 | MP | 2018-09-17 | A11 | 山竹撲至 大亞灣台山核電站平安 2死交通全癱瘓 廣東停工停課 |
| 24 | MP | 2018-09-17 | A02 | 政府風前響鑼做準備 建制泛民罕有齊肯定 |
| 25 | MP | 2018-09-17 | A06 | 日出康城領都爆玻璃 將軍澳成「澤國」 水淹停車場 |
| 26 | MP | 2018-09-17 | A06 | 澳門今停課 政府部門停工 |
| 27 | MP | 2018-09-17 | A02 | 昨889航班取消 重新編配料需兩日 |
| 28 | MP | 2018-09-17 | B07 | 馬場設施受損葉楚航如常開工 |
| 29 | MP | 2018-09-17 | A04 | 城門河氾濫 淹浸停車場 |
| 30 | MP | 2018-09-17 | A01 | 杏花邨再成澤國 渠噴水柱垃圾湧路 |
| 31 | MP | 2018-09-17 | B05 | 颱風吹停澳門9月賭收 分析：降至單位數增幅 |
| 32 | MP | 2018-09-17 | A03 | 大澳水浸及腰 留守商戶再破財 海水高度升至3.8米 沙包難阻 |
| 33 | MP | 2018-09-17 | A06 | 大廈搖晃市民「暈浪」 工程師稱屬正常 |
| 34 | MP | 2018-09-17 | A04 | 逾300市民受傷 冷氣機吹入屋 擊傷兩戶主 |
| 35 | MP | 2018-09-17 | A03 | 海水倒灌曱甴湧鯉魚門 |
| 36 | MP | 2018-09-17 | A04 | 地盤支架吹塌 鐵皮屋險墜街 |
| 37 | MP | 2018-09-18 | A18 | 「山竹」讓人重見香港久違的專業 |
| 38 | MP | 2018-09-18 | A02 | 菜肉魚供應同減 商會料菜價貴兩成 |
| 39 | MP | 2018-09-18 | A01 | 昨交通半癱 政黨責未籲停工 上班無路爆民怨 運署今晨通幹道 |
| 40 | MP | 2018-09-18 | A11 | 林鄭親檢討風後上班混亂 運署港鐵巴士急改善 |
| 41 | MP | 2018-09-18 | A11 | 李家超講錯「山竹」做「天竺」 |
| 42 | MP | 2018-09-18 | A17 | 塌樹爆玻璃 最少59校損毁 教局維修無期 學校盼撥應急資源 |
| 43 | MP | 2018-09-18 | A17 | 男拔生復課須清潔校園 |
| 44 | MP | 2018-09-18 | A02 | 排隊取山水 荃威居民斥水署送水慢 |
| 45 | MP | 2018-09-18 | A04 | 辦公室爆玻璃 經理：似災難片 失大量文件合約 保險界指不賠 |
| 46 | MP | 2018-09-18 | A02 | 專家籲未來一周 勿近樹下斜坡 |
| 47 | MP | 2018-09-18 | A17 | 北區校憂跨境生滯校巴 |
| 48 | MP | 2018-09-18 | A03 | 粉嶺成「孤島」 有人折騰8句鐘方脫身 |
| 49 | MP | 2018-09-18 | A04 | 工會促立法保障災後僱員 |
| 50 | MP | 2018-09-18 | A04 | 僱主會：毋須政府籲停工 |
| 51 | MP | 2018-09-18 | A06 | 智利女發起清理海濱 一呼百應 來港4月住將軍澳 「為香港人感驕傲」 |
| 52 | MP | 2018-09-18 | A02 | 中小幼今續停課 校方須照顧回校生 幼園稱教師復工 返校童可做手工聽故事 |
| 53 | MP | 2018-09-18 | A11 | 偕市民清樹 議員著錯褲蟻咬 |
| 54 | MP | 2018-09-18 | A18 | 高調防風對 忽略善後錯 |
| 55 | MP | 2018-09-18 | A03 | 車廠路不通 七成半巴士線癱瘓 清理一日 34廠已清障礙粉 |
| 56 | MP | 2018-09-19 | A04 | 稱有朋友讚賞政府處理「山竹」來襲 林鄭：拿特首出氣沒問題 檢討天災應對 |
| 57 | MP | 2018-09-19 | A14 | 運署App有字無路况 業界斥無助發放資訊 |
| 58 | MP | 2018-09-19 | A02 | 西貢億元遊艇擱淺 海面見浮屍漂逾10噸垃圾 |
| 59 | MP | 2018-09-19 | A04 | 斥巨資保留 古樹不敵「山竹」倒地 |
| 60 | MP | 2018-09-19 | A26 | 風後安排 考驗政府貼地程度 |
| 61 | MP | 2018-09-19 | A02 | 廢物轉運站風後迫爆 環署捱轟 垃圾車排長龍 司機批欠評估應變 |
| 62 | MP | 2018-09-19 | A02 | 兩成外判清潔工 掛8號波要上班 |
| 63 | MP | 2018-09-19 | A04 | 中小幼今復課 林鄭：可提供維修撥款 |
| 64 | MP | 2018-09-19 | A27 | 風後現形記 「甩」市民之所急 |
| 65 | MP | 2018-09-19 | A04 | 青年自發清理街道 冀消社會標籤 |
| 66 | MP | 2018-09-20 | A04 | 全港泳灘仍關閉 重開無期 深水灣淋浴間堆沙逼近天花 |
| 67 | MP | 2018-09-20 | A11 | 引報料指西九站爆玻璃 鄭松泰稱「錯覺」道歉 |
| 68 | MP | 2018-09-20 | B10 | 遭「山竹」摧毀訓練基地 港賽艇協會眾籌600萬重建 |
| 69 | MP | 2018-09-20 | D05 | 特首的分寸 |
| 70 | MP | 2018-09-20 | A24 | 記者年年打風都要身犯險境 其實有何意義？ |
| 71 | MP | 2018-09-20 | A04 | 解放軍艦交椅洲擱淺 駐港部隊：登陸避險 |
| 72 | MP | 2018-09-20 | A04 | 7校仍停課 黃埔學校三成窗未修好 |
| 73 | MP | 2018-09-20 | A04 | 清潔工會促提供保護裝備 |
| 74 | MP | 2018-09-21 | D05 | 科普與謠言 |
| 75 | MP | 2018-09-21 | A04 | 四成僱員8號風球黑雨要上班 逾半無津貼 |
| 76 | MP | 2018-09-21 | A02 | 西貢處理廠損毁 污水排牛尾海 無二級淨化恐含菌 修復需時 |
| 77 | MP | 2018-09-21 | A02 | 彭福地標樹倒塌 詹志勇：吊直可救回 |
| 78 | MP | 2018-09-21 | A02 | 深水灣粉嶺高球場 風吹損毁暫關閉 |
| 79 | MP | 2018-09-21 | A04 | 避風中心牀墊毛氈用完即棄 |
| 80 | MP | 2018-09-21 | A26 | 學校風後維修 教局特別津貼 |
| 81 | MP | 2018-09-21 | A16 | 「幸福專家」為小麗站台 鄺俊宇「逗號」喻風災善後欠佳 |
| 82 | MP | 2018-09-22 | A06 | 打風重創 泳灘中秋不供賞月 污水排海 西貢食肆照泵水養海鮮 |
| 83 | MP | 2018-09-22 | A06 | 送餐車打風受阻 佛教醫院險斷糧 |
| 84 | MP | 2018-09-22 | A06 | 民航通訊「巨球」穿洞 |
| 85 | MP | 2018-09-23 | P02 | 山竹啟示錄： 怒中有淚的港人傳奇 |
| 86 | MP | 2018-09-23 | A06 | 風後現身 陳帆︰團隊夙夜不眠 |
| 87 | MP | 2018-09-23 | A06 | 林超英：海水升 建人工島逆天行道 前土力處長：可築防波堤解決 |
| 88 | MP | 2018-09-23 | P03 | 廢木去哪兒? |
| 89 | MP | 2018-09-23 | S05 | 「山竹」條例 |
| 90 | MP | 2018-09-23 | A06 | 義工大軍周末出動 多區「大掃除」 |
| 91 | MP | 2018-09-23 | A06 | 斷樹收集處 兩日堆如山 |
| 92 | MP | 2018-09-24 | A13 | 風後停駛被批通報差 巴士業界怨被屈 |
| 93 | MP | 2018-09-24 | A21 | 教局風災津貼今周公布 |
| 94 | MP | 2018-09-25 | A04 | 風後中秋夜石澳冷清 維園依舊摩肩接踵 |
| 95 | MP | 2018-09-25 | A06 | 林鄭風災後首落區 稱復修需時籲忍耐 |
| 96 | MP | 2018-09-25 | D05 | 數字管理堅離地 |
| 97 | MP | 2018-09-26 | A10 | 天文台徵「山竹」片相 感謝卡贈首500人 |
| 98 | MP | 2018-09-26 | A03 | 涉私人地段斜坡 5車被砸求助政府多天無果 山竹塌樹壓車場 負責人查察「災情」被困 |
| 99 | MP | 2018-09-26 | A02 | 陳凱欣「山竹啟示」 宣布積極考慮參選 |
| 100 | MP | 2018-09-26 | A03 | 啟德堆樹區快爆滿 團體倡細枝放社區泥壤 |
| 101 | MP | 2018-09-26 | A03 | 斜坡業權 地署土拓署網頁可查 |
| 102 | MP | 2018-09-27 | A13 | 「山竹啟示」有負評 陳凱欣未出閘先捱嘲 |
| 103 | MP | 2018-09-27 | A10 | 風後民調 林鄭評分新低 |
| 104 | MP | 2018-09-27 | A10 | 山竹餘波 吐露港渡海泳取消 拯溺獨木舟吹走 沙灘海牀遺金屬片 |
| 105 | MP | 2018-09-27 | A13 | 荒廢群組發訊 馬逢國投訴塌樹未清理 |
| 106 | MP | 2018-09-28 | A08 | 西貢污水廠下月提升污水處理 |
| 107 | MP | 2018-09-28 | A26 | 中小學風災津貼15萬 |
| 108 | MP | 2018-09-29 | A04 | 五成受訪市民：山竹翌日應停工 |
| 109 | MP | 2018-09-29 | A04 | 林鄭認風災善後有改善空間 黃金周過半營地續關閉 |
| 110 | MP | 2018-09-29 | A11 | 鄉議局200萬賑風災 劉業強獨捐百萬 |
| 111 | MP | 2018-09-30 | S08 | 四萬宗塌樹 四萬戶停電 塌樹也是基建失靈元兇？ |
| 112 | MP | 2018-09-30 | S03 | 倒下的樹何去何從？ 重植？ 堆填？ 再造？ |
| 113 | MP | 2018-09-30 | A04 | 山竹襲港後兩周 陳茂波清塌樹 |
| 114 | MP | 2018-10-01 | A04 | 風後塌樹4.6萬宗 政府倡補種原生樹 專家：應配合路邊環境 勿一概而論 |
| 115 | MP | 2018-10-01 | A02 | 超強颱風增 人工島中途改設計 |
| 116 | MP | 2018-10-03 | A13 | 山竹過後 611靈糧堂千人受浸 |
| 117 | MP | 2018-10-03 | A12 | 黃偉綸：街頭植樹年底推指南 本地外來品種各半 「樹種非唯一考慮」 |
| 118 | MP | 2018-10-03 | A12 | 深水灣淋浴間重見天日 |
| 119 | MP | 2018-10-04 | A03 | 百年一遇估算 56年兩次「打破」 海堤指引增高 不及山竹溫黛潮位 |
| 120 | MP | 2018-10-04 | A12 | 張建宗率9高官講山竹應對 兩黨提停工私人草案 |
| 121 | MP | 2018-10-04 | A12 | 山竹救災 消防拍片多謝手足 |
| 122 | MP | 2018-10-04 | A03 | 山竹襲港 大廈搖晃屬正常 |
| 123 | MP | 2018-10-04 | D07 | 塌樹枯枝的出路 |
| 124 | MP | 2018-10-04 | A03 | 過去冷待 土拓署研改善杏花邨海堤 |
| 125 | MP | 2018-10-04 | A03 | 發展海綿城市 建築師倡新臨海區建蓄洪池 |
| 126 | MP | 2018-10-05 | A04 | 山竹後不停工 張建宗：絕非憂經濟 議員倡賦權特首 政府稱開放態度 |
| 127 | MP | 2018-10-07 | P09 | 種不修，官之過 樹失救，財之禍 |
| 128 | MP | 2018-10-07 | A04 | 古樹吹移位 專家批修花槽當無事 位處栢麗大道 憂增倒塌風險 |
| 129 | MP | 2018-10-09 | B06 | 颱風爆窗 家居保未必保 山竹過後新單銷售增五成 |
| 130 | MP | 2018-10-09 | A08 | 打風窗戶貼膠紙 工程師︰碎片變大更危險 |
| 131 | MP | 2018-10-13 | A10 | 山竹重創 將軍澳海濱單車徑料修半年 |
| 132 | ODN | 2018-09-11 | A17 | 雙風前後腳襲港 下周恐掛八號波 |
| 133 | ODN | 2018-09-12 | A01 | 超強颱風 勁過天鴿 山竹周末直撲香港 |
| 134 | ODN | 2018-09-12 | A01 | 兩岸嚴陣以待 港府防災歎慢板 |
| 135 | ODN | 2018-09-13 | A02 | 風王撲港禍臨頭政府防災慢半拍 |
| 136 | ODN | 2018-09-13 | A02 | 正論：超強颱風殺到來 預防不力必成災 |
| 137 | ODN | 2018-09-13 | A02 | 威力近年罕見山竹氣壓低見905百帕 |
| 138 | ODN | 2018-09-13 | A02 | 鯉魚門及杏花邨搬沙包拉閘防水浸 |
| 139 | ODN | 2018-09-13 | A02 | 天鴿陰霾 澳門居民「撲水」儲糧 |
| 140 | ODN | 2018-09-14 | A02 | 山竹10級風力 逐步進逼 |
| 141 | ODN | 2018-09-14 | A02 | 周日風暴潮 或掀內港水浸 |
| 142 | ODN | 2018-09-14 | A02 | 部門嚴陣防「風王」清理渠口 加固樹木 |
| 143 | ODN | 2018-09-14 | A14 | 政情：立會訪英團 驚打風冇得走 諗計延期返港 |
| 144 | ODN | 2018-09-14 | A02 | 鯉魚門 商戶堆沙包貼膠紙自救 |
| 145 | ODN | 2018-09-15 | A01 | 12級颶風 隨時10號波 山竹兇猛3000人大疏散 |
| 146 | ODN | 2018-09-15 | A02 | 防洪牆冇影天鴿慘況恐重現 |
| 147 | ODN | 2018-09-15 | A14 | 政情：公民講場：山竹來勢洶洶吹岑智明返港 |
| 148 | ODN | 2018-09-15 | A02 | 跨部門如臨大敵「要有總動員準備」 |
| 149 | ODN | 2018-09-15 | A01 | 鯉魚門商戶與舖共存亡 義工團助大澳老弱避災 |
| 150 | ODN | 2018-09-16 | A01 | 山竹封港 千二航班取消航空大癱瘓 |
| 151 | ODN | 2018-09-16 | A02 | 山竹襲港來勢洶 防風應變大考驗 |
| 152 | ODN | 2018-09-16 | A01 | 240離港團焗調動或取消 |
| 153 | ODN | 2018-09-16 | A02 | 庇護中心少入住大澳居民多留守 |
| 154 | ODN | 2018-09-16 | A02 | 杏花邨車場曾受災 十餘架車照泊 |
| 155 | ODN | 2018-09-16 | A13 | 140義工烏溪沙淨灘 推動保護生態 |
| 156 | ODN | 2018-09-16 | A10 | 政情：公民講場：張建宗廢噏「膠紙加價係教育過程」 |
| 157 | ODN | 2018-09-16 | A01 | 部分路段鐵路 9號波即停服務 |
| 158 | ODN | 2018-09-16 | A15 | 颱風工作無保障 團體批僱主剝削促修例 |
| 159 | ODN | 2018-09-16 | A02 | 流浮山居民 鐵皮屋「五花大綁」 |
| 160 | ODN | 2018-09-17 | A01 | 10號波風王山竹 吹散香港 |
| 161 | ODN | 2018-09-17 | A02 | 嚴陣布防 杏花邨又成澤國 |
| 162 | ODN | 2018-09-17 | A04 | 住宅商廈同食風 爆窗玻璃碎入屋 |
| 163 | ODN | 2018-09-17 | A02 | 大埔多條隧道被淹浸險沒頂 |
| 164 | ODN | 2018-09-17 | A02 | 889航班取消 300人滯留機場 |
| 165 | ODN | 2018-09-17 | A02 | 大澳水深及腰 鯉魚門大停電 |
| 166 | ODN | 2018-09-17 | A14 | 圖爆竊豪宅三蛇匪就擒 |
| 167 | ODN | 2018-09-17 | A04 | 多區大樹倒塌水浸 道路封閉 |
| 168 | ODN | 2018-09-17 | A04 | 地盤升降機鐵軸飛墮壓唐樓 |
| 169 | ODN | 2018-09-17 | A10 | 潤物有聲：人禍比天災可惡 |
| 170 | ODN | 2018-09-18 | A02 | 正論：颱風並非真考驗 善後方是大問題 |
| 171 | ODN | 2018-09-18 | A04 | 山竹風力勁過1979年荷貝 |
| 172 | ODN | 2018-09-18 | A10, A11 | 山竹發惡 香江落泊 |
| 173 | ODN | 2018-09-18 | A28 | 獅子山上：打風後全面執漏 |
| 174 | ODN | 2018-09-18 | A01 | 山竹反轉香江 政府善後無方 癱瘓交通 冇車返工 全城怒火 |
| 175 | ODN | 2018-09-18 | A01 | 塌樹堵路 乏人清理 運輸業促設災後應變部隊 |
| 176 | ODN | 2018-09-18 | A04 | 逾千航班再編配 機場料今復正常 |
| 177 | ODN | 2018-09-18 | A02 | 國泰空廚圖叫員工還風假 |
| 178 | ODN | 2018-09-18 | A02 | 多校嚴重受損 全港今續停課 |
| 179 | ODN | 2018-09-18 | A08 | 垃圾污水周街倒 商戶缺德 |
| 180 | ODN | 2018-09-18 | A06 | 滿街垃圾 市民自發清理贏讚 |
| 181 | ODN | 2018-09-18 | A02 | 停課不停工林鄭被圍攻 |
| 182 | ODN | 2018-09-19 | B02 | 山竹保險賠償超天鴿 |
| 183 | ODN | 2018-09-19 | A19 | 山竹吹襲 港珠澳橋門窗滲漏 |
| 184 | ODN | 2018-09-19 | A01 | 滿城垃圾未清理 等政府 蛇都死 不如靠自己 |
| 185 | ODN | 2018-09-19 | A02 | 僱主扣薪又扣假 狠狠摑林鄭一巴 |
| 186 | ODN | 2018-09-19 | A02 | 停課不停工 交通亂晒龍 議員炮轟陳帆失蹤 |
| 187 | ODN | 2018-09-19 | A02 | 正論：颱風後收拾殘局 靠官僚不如自救 |
| 188 | ODN | 2018-09-19 | A04 | 冧樹布危途 復課存變數 |
| 189 | ODN | 2018-09-19 | A17 | 41公眾泳灘暫停開放 或阻賞月 |
| 190 | ODN | 2018-09-19 | A04 | 停課剩8萬飯盒 免費派街坊 |
| 191 | ODN | 2018-09-19 | A01 | 環保署懶理垃圾圍城 被轟態度官僚 |
| 192 | ODN | 2018-09-19 | A01 | 橫頭磡邨塌樹 樹枝直插單位 |
| 193 | ODN | 2018-09-20 | A13 | 政情：山竹癱瘓交通 連日玩失蹤 陳帆蒲頭零交代 |
| 194 | ODN | 2018-09-20 | A10 | 風災禍港幾回 官患無限輪迴 |
| 195 | ODN | 2018-09-20 | A10 | 政府抗災多甩漏 跨部門未能善後 |
| 196 | ODN | 2018-09-20 | A02 | 不敵山竹狂風 三古樹倒塌 |
| 197 | ODN | 2018-09-20 | A13 | 政情：報錯西九站爆玻璃 鄭松泰認衰道歉 |
| 198 | ODN | 2018-09-20 | A02 | 毓民特區：救災不力 善後無方 林鄭欠港人道歉 |
| 199 | ODN | 2018-09-20 | A02 | 上學越森林 一步一驚心 |
| 200 | ODN | 2018-09-20 | A04 | 牛皮沙村電線杆壓屋 住戶緊急求助 部門唔急 |
| 201 | ODN | 2018-09-20 | A04 | 山竹遺禍 兩鐵騎士一死一傷 |
| 202 | ODN | 2018-09-20 | A02 | 正論：環保署表現垃圾 官無能猛於天災 |
| 203 | ODN | 2018-09-20 | A04 | 議員促立法取代現行打風《工作守則》 |
| 204 | ODN | 2018-09-20 | A01 | 環保署失職 垃圾大堆積 |
| 205 | ODN | 2018-09-20 | A02 | 運頭塘邨半數防煙門吹毀 |
| 206 | ODN | 2018-09-21 | A14 | 中環出更：擔心山竹太強 李鏡波蒙古趕返港防風 |
| 207 | ODN | 2018-09-21 | A02 | 尖沙咀百年老樹遭吹倒 專家：可扶正樹身加金屬支架拯救 |
| 208 | ODN | 2018-09-21 | A01 | 山竹吹毀排污設施 西貢冧廠 政府維修慢 南區爆渠 臭水湧泳灘 |
| 209 | ODN | 2018-09-21 | A04 | 43%僱員八號波黑雨返工 多不獲交通津貼 惡劣天氣遲到要扣薪 |
| 210 | ODN | 2018-09-21 | A01 | 多區海灘泳池損毀嚴重 修復需時 |
| 211 | ODN | 2018-09-21 | A22 | 功夫茶：垃圾圍城未解決 污水長流更不堪 |
| 212 | ODN | 2018-09-21 | A14 | 政情：教聯會 教協鬥法 叫楊潤雄科水救災 |
| 213 | ODN | 2018-09-21 | A02 | 多區需復修及重建 泛民促立會商風災善後 |
| 214 | ODN | 2018-09-21 | A04 | 山竹襲港期間無影 陳帆被促下台 |
| 215 | ODN | 2018-09-21 | A02 | 任由垃圾圍城市民司機鬧爆政府 |
| 216 | ODN | 2018-09-21 | A08 | 鄉村多塌樹 居民自救不靠政府 |
| 217 | ODN | 2018-09-21 | A01 | 一級處理難除大腸桿菌 |
| 218 | ODN | 2018-09-21 | A02 | 庇護中心床墊疑瞓兩晚就丟 網民轟「政府帶頭做大嘥鬼」 |
| 219 | ODN | 2018-09-22 | A36 | 潤物有聲：山竹再猛 吹不倒一眾廢官 |
| 220 | ODN | 2018-09-22 | A18 | 中環出更：張炳良護短 唔信陳帆山竹善後潛水 |
| 221 | ODN | 2018-09-22 | A12 | 探射燈：舊公屋危機四伏老化失修重建龜速 |
| 222 | ODN | 2018-09-22 | A24 | 紅旗封灘69歲婦溺斃 |
| 223 | ODN | 2018-09-22 | A06 | 垃圾處理量飆四成 未分類增壓縮時間 |
| 224 | ODN | 2018-09-22 | A27 | 教局「點人頭」有學校流失跨境生 |
| 225 | ODN | 2018-09-23 | A12 | 中環出更：山竹期間「冇影」 陳帆現身解畫 |
| 226 | ODN | 2018-09-23 | A08 | 促加強沿海防波堤 減風災損毀 |
| 227 | ODN | 2018-09-23 | A08 | 海洋生態恐釀災 善後進展需交代 |
| 228 | ODN | 2018-09-23 | A02 | 山竹吹襲「零死亡」林超英為港慶幸 |
| 229 | ODN | 2018-09-23 | A02 | 政府難靠義工團四出救災 |
| 230 | ODN | 2018-09-23 | A02 | 環署設臨時木料廢物收集處 |
| 231 | ODN | 2018-09-24 | A18 | 氣候暖化 林超英警告 港要面對比山竹更強風暴 |
| 232 | ODN | 2018-09-24 | A28 | 獅子山上：港人自救 拆除危機 |
| 233 | ODN | 2018-09-24 | A08 | 東方民調：風後無緊急應變 政府倒瀉籮蟹 |
| 234 | ODN | 2018-09-24 | A18 | 東龍洲毀容 毒膠遍野 |
| 235 | ODN | 2018-09-24 | A18 | 羅致光對一刀切立法風後停工有保留 |
| 236 | ODN | 2018-09-24 | A08 | 東方民調：官僚麻痹災後懶理 市民無助逼靠自己 |
| 237 | ODN | 2018-09-24 | A16 | 校園受損 教局擬推風災津貼 |
| 238 | ODN | 2018-09-25 | A06 | 探射燈：強颱摧毀天台屋基層無助逼露宿 |
| 239 | ODN | 2018-09-25 | A13 | 收集處暫存千車風災廢木 篩選重用 |
| 240 | ODN | 2018-09-25 | A08 | 災後清理辛勞 工會促津貼外判工 |
| 241 | ODN | 2018-09-26 | A01 | 災後10日政府懶清爛樹圍城 啟德臨時堆樹區爆滿 |
| 242 | ODN | 2018-09-26 | A02 | 死魚未處理 臭爆筷子基 |
| 243 | ODN | 2018-09-26 | A01 | 基建挖泥損樹根 港樹木難抵強風 |
| 244 | ODN | 2018-09-26 | A02 | 唔等政府 自己清樹 六旬漢困山坡 |
| 245 | ODN | 2018-09-27 | A17 | 教局發風災津貼 中小學上限15萬 |
| 246 | ODN | 2018-09-27 | A19 | 山竹襲港後 林鄭民望創新低 |
| 247 | ODN | 2018-09-27 | A16 | 風災後垃圾遍海 吐露港渡海泳取消 |
| 248 | ODN | 2018-09-28 | A19 | 判斷停工避談路況分級制運輸署長捱轟 |
| 249 | ODN | 2018-09-28 | A19 | 西貢污水廠新海堤 料下周三完工 |
| 250 | ODN | 2018-09-28 | A15 | 十一假期災後營未修復多處不宜遠足 |
| 251 | ODN | 2018-09-28 | A12 | 政情：風災襲村郊 鄉議局設善後小組自救 |
| 252 | ODN | 2018-09-28 | A19 | 教局風災津貼 明年一月始發放 |
| 253 | ODN | 2018-09-29 | A32 | 山竹力場 官僚現形 |
| 254 | ODN | 2018-09-29 | A22 | 災後兩周 港府清走八千噸廢物 |
| 255 | ODN | 2018-09-29 | A08 | 房署官僚礙清理 災後危樹藏殺機 |
| 256 | ODN | 2018-09-30 | A04 | 大澳1.5億防洪河堤乏成效 居民倡建大閘 |
| 257 | ODN | 2018-09-30 | A04 | 營地滿目瘡痍 十一假期內地客掉頭走 |
| 258 | ODN | 2018-09-30 | A08 | 中環出更：陳國基鄧以海石澳視察落手清塌樹 |
| 259 | ODN | 2018-09-30 | A08 | 公民講場：波叔清樹自嘲慢 派錢千祈唔好慢 |
| 260 | ODN | 2018-09-30 | A12 | 35義工西貢清95公斤海上垃圾 |
| 261 | ODN | 2018-10-01 | A04 | 陳肇始賴山竹「吹走老鼠竇」 |
| 262 | ODN | 2018-10-01 | A04 | 石澳災情被遺忘 議員促撥款還原 |
| 263 | ODN | 2018-10-01 | A04 | 4.6萬宗塌樹 逾半補植原生品種 |
| 264 | ODN | 2018-10-02 | A24 | 獅子山上：的起心肝 派定心丸 |
| 265 | ODN | 2018-10-02 | A01 | 公屋壞升降機 維修等四日 |
| 266 | ODN | 2018-10-02 | A06 | 社福服務全天運作 施家殷嘆難風後停工 |
| 267 | ODN | 2018-10-02 | A08 | 淨灘行動檢30年前膠樽 |
| 268 | ODN | 2018-10-03 | A10 | 綠色先鋒：清潔香港還原社區 |
| 269 | ODN | 2018-10-03 | A02 | 風災善後不力高官民望插水 |
| 270 | ODN | 2018-10-03 | A02 | 塌樹壓頂無人理 天災無情官無恥 |
| 271 | ODN | 2018-10-03 | A01 | 塌樹毀家園16日無援 政府一味推市民一殼淚 |
| 272 | ODN | 2018-10-03 | A02 | 發展局跪低 年底推種樹指南 |
| 273 | ODN | 2018-10-03 | A28 | 聯合救災體現公民社會 |
| 274 | ODN | 2018-10-03 | A02 | 部門救災大細超偏遠村落等餐死 |
| 275 | ODN | 2018-10-03 | A02 | 政黨促杏花邨增設弱波石 |
| 276 | ODN | 2018-10-03 | A13 | 中環出更：離島風災善後難 陳國基再號召義工清塌樹 |
| 277 | ODN | 2018-10-03 | A13 | 公民講場：黃偉綸補鑊擬種防風樹 |
| 278 | ODN | 2018-10-04 | A19 | 蛇蟲鼠蟻禍港 食環署最窩囊 |
| 279 | ODN | 2018-10-04 | A04 | 吉澳百米垃圾路政府懶理 |
| 280 | ODN | 2018-10-04 | A04 | 災後檢視42.4萬棵樹 僅17%完成善後 |
| 281 | ODN | 2018-10-05 | A01 | 官員塞責議員圍插 山竹天災政府人禍 |
| 282 | ODN | 2018-10-06 | A32 | 潤物有聲：拖到出年風季再算 |
| 283 | ODN | 2018-10-06 | A06 | 迴避生產者責任制 廢膠圍城 |
| 284 | ODN | 2018-10-06 | A13 | 考慮不周 市區屢揭種錯樹 |
| 285 | ODN | 2018-10-06 | A06 | 規管即棄塑膠 欠時間表 |
| 286 | ODN | 2018-10-07 | A01 | 玉器市場外 神秘環保斗 部門拒運走 風災垃圾懶清理政府搞臭油麻地 |
| 287 | ODN | 2018-10-08 | A02 | 天文台長：山竹過後勿放鬆 11月颱風一樣兇 |
| 288 | ODN | 2018-10-08 | B02 | 蘇黎世力拓家居保市場 |
| 289 | ODN | 2018-10-08 | A08 | 取締村屋僭建 迫在眉睫 |
| 290 | ODN | 2018-10-08 | A02 | 山竹吹落楓香樹葉 大棠美景恐不再「紅」 |
| 291 | ODN | 2018-10-08 | A02 | 風災停工 商界反對立法 |
| 292 | ODN | 2018-10-08 | A28 | 官僚卸責 累積怨懟 |
| 293 | ODN | 2018-10-08 | A08 | 執法弱拆彈慢 人禍惡過天災 |
| 294 | ODN | 2018-10-08 | A02 | 斜坡危樹架欄杆 路人心慌 |
| 295 | ODN | 2018-10-09 | A04 | 將軍澳風後海濱變爛地 完成復修遙無期 |
| 296 | ODN | 2018-10-09 | A04 | 工程師指窗貼膠紙 爆裂更危險 |
| 297 | ODN | 2018-10-09 | A06 | 鄉郊塌樹未清 入秋隨時釀火警 |
| 298 | ODN | 2018-10-09 | A04 | 打風缺勤 大埔醫院扣清潔工假 |
| 299 | ODN | 2018-10-10 | A28 | 香港電力供應 未來何去何從 |
| 300 | ODN | 2018-10-11 | A14 | 山竹賠償額料達十億 破保險界紀錄 |
| 301 | ODN | 2018-10-13 | A08 | 山竹摧殘西貢污水廠冀年底修復 |
