# Supplementary material for: Longitudinal multiplexity and structural constraints of online emergency collaborative networks: A tale of two Chinese societies
Source: PLoS One. 2023 Jul 27;18(7):e0289277. doi: 10.1371/journal.pone.0289277 (PMC10374111; doi:10.1371/journal.pone.0289277)
Supplement: S3 Table — (DOCX) [file pone.0289277.s003.docx]

**Organizational actors identified from Shenzhen on Weibo**

| No | Organization | Weibo account | No. of followers | No. of followees | No. of tweets | Tweets during the time frame |
| --- | --- | --- | --- | --- | --- | --- |
| 1 | 宝安区翻身实验学校 | https://weibo.com/u/5050177163 | 286 | 115 | 608 | Y |
| 2 | 宝安区福永人民医院 | https://weibo.com/u/3613716011 | 275 | 58 | 430 | Y |
| 3 | 宝安区公安分局 | <https://weibo.com/u/2736900633> | 30057 | 328 | 4576 | Y |
| 4 | 宝安区交警大队 | https://weibo.com/u/2837663394 | 43191 | 584 | 65548 | Y |
| 5 | 宝安区教育局 | https://weibo.com/baoanedu | 17740 | 104 | 3567 | Y |
| 6 | 宝安区委 | <https://weibo.com/baoanfabu> | 36261 | 286 | 15085 | Y |
| 7 | 宝安人民医院 | <https://weibo.com/barmyy> | 515 | 20 | 359 | Y |
| 8 | 宝安中医院（集团） | <https://weibo.com/u/3538296284> | 564 | 67 | 298 | Y |
| 9 | 大鹏半岛国家地质公园 | https://weibo.com/szgeopark | 864 | 82 | 518 | Y |
| 10 | 大鹏区公安分局 | https://weibo.com/u/5291405391 | 814 | 28 | 666 | Y |
| 11 | 大亚湾核电 | <https://weibo.com/wbofdnmc> | 9138 | 349 | 2246 | Y |
| 12 | 福田区公安分局 | https://weibo.com/futiangongan | 46426 | 395 | 7766 | Y |
| 13 | 福田区交警大队 | https://weibo.com/ftpolice | 23084 | 219 | 67296 | Y |
| 14 | 福田区政府 | <https://weibo.com/u/2858056932> | 68713 | 252 | 18666 | Y |
| 15 | 光明区委 | <https://weibo.com/u/2858056760> | 26209 | 182 | 18594 | Y |
| 16 | 广东省民政厅 | <https://weibo.com/gdsmzt> | 29659 | 105 | 4585 | Y |
| 17 | 广东省水利厅 | https://weibo.com/u/6090441896 | 531 | 100 | 1674 | Y |
| 18 | 广东省政府 | <https://weibo.com/guangdongfabu> | 3321655 | 388 | 30752 | Y |
| 19 | 国家应急管理部 | <https://weibo.com/gjajzj> | 1626201 | 217 | 11087 | Y |
| 20 | 红树林基金会 | <https://weibo.com/szmcf> | 76904 | 806 | 4035 | Y |
| 21 | 华润万家 | <https://weibo.com/crva> | 53593 | 307 | 2444 | Y |
| 22 | 龙岗区公安局 | <https://weibo.com/policelg> | 33606 | 176 | 3091 | Y |
| 23 | 龙岗区交警大队 | <https://weibo.com/u/2861504544> | 70338 | 634 | 83224 | Y |
| 24 | 龙岗区人民医院 | <https://weibo.com/szlgrmyy> | 147 | 69 | 143 | Y |
| 25 | 龙岗区政府 | <https://weibo.com/u/2233211910> | 435736 | 235 | 27139 | Y |
| 26 | 龙华区公安分局民治派出所 | <https://weibo.com/szlhga> | 10731 | 86 | 4458 | Y |
| 27 | 龙华区委 | https://weibo.com/szlhfb | 36716 | 192 | 13255 | Y |
| 28 | 罗湖区公安分局 | <https://weibo.com/szlhfj> | 58892 | 792 | 5934 | Y |
| 29 | 罗湖区人民医院 | https://weibo.com/u/5270564920 | 46176 | 49 | 916 | Y |
| 30 | 罗湖区委 | <https://weibo.com/u/2694355870> | 102194 | 334 | 19398 | Y |
| 31 | 南方电网 | <https://weibo.com/csgcn> | 167627 | 722 | 14294 | Y |
| 32 | 南航深圳分公司 | https://weibo.com/czshenzhen | 13071 | 302 | 1592 | Y |
| 33 | 南山区交警大队 | <https://weibo.com/u/2789608094> | 29526 | 326 | 41411 | Y |
| 34 | 南山区政府 | <https://weibo.com/nanshanfabu> | 116535 | 193 | 14017 | Y |
| 35 | 坪山区政府 | <https://weibo.com/psfb> | 106581 | 127 | 14962 | Y |
| 36 | 前海管理局 | <https://weibo.com/u/3096705901> | 51024 | 187 | 5344 | Y |
| 37 | 前海开发投资控股有限公司（前海控股） | https://weibo.com/qianhaikonggu | 118 | 58 | 670 | Y |
| 38 | 深圳宝安国际机场 | <https://weibo.com/szairport> | 94831 | 198 | 2293 | Y |
| 39 | 深圳地铁义工联 | <https://weibo.com/u/3196922514> | 13380 | 263 | 5677 | Y |
| 40 | 深圳地铁运营总部 | <https://weibo.com/szmcservice> | 1865065 | 153 | 28254 | Y |
| 41 | 深圳蓝天救援队 | https://weibo.com/szbsr | 2313 | 321 | 1917 | Y |
| 42 | 深圳市城管局 | https://weibo.com/u/2858032432 | 99053 | 236 | 17986 | Y |
| 43 | 深圳市儿童医院 | https://weibo.com/szetyy | 13751 | 433 | 1954 | Y |
| 44 | 深圳市公安局 | https://weibo.com/szga | 3793694 | 1404 | 74682 | Y |
| 45 | 深圳市公安局交通警察局 | <https://weibo.com/shenzhenjiaojing> | 2727744 | 763 | 205524 | Y |
| 46 | 深圳市公安消防支队 | https://weibo.com/u/2216688114 | 24662 | 339 | 1877 | Y |
| 47 | 深圳市供电局 | <https://weibo.com/powergrid> | 643453 | 455 | 4722 | Y |
| 48 | 深圳市规划和国土资源委员会（市海洋局） | <https://weibo.com/u/2858032360> | 45158 | 245 | 3631 | Y |
| 49 | 深圳市建筑工务署 | https://weibo.com/szgws | 63645 | 100 | 2148 | Y |
| 50 | 深圳市交通运输委员会 | <https://weibo.com/szjwfb> | 663280 | 339 | 18612 | Y |
| 51 | 深圳市教育局 | <https://weibo.com/u/2859077682> | 896423 | 662 | 31369 | Y |
| 52 | 深圳市乐琪实业发展有限公司 | https://weibo.com/u/5144372621 | 5468 | 59 | 350 | Y |
| 53 | 深圳市民政局 | <https://weibo.com/szsmzj> | 410208 | 968 | 13033 | Y |
| 54 | 深圳市气象局（台） | https://weibo.com/szmb | 2011348 | 281 | 59381 | Y |
| 55 | 深圳市燃气集团股份有限公司 | <https://weibo.com/szgas> | 2951 | 261 | 3500 | Y |
| 56 | 深圳市水务集团 | <https://weibo.com/watergroup> | 8070 | 71 | 1470 | Y |
| 57 | 深圳市卫计委 | https://weibo.com/szhpfpc | 609012 | 436 | 19897 | Y |
| 58 | 深圳市新现代社工服务中心 | https://weibo.com/dalangsq | 525 | 94 | 648 | Y |
| 59 | 深圳市应急办 | https://weibo.com/szsafety2016 | 125616 | 101 | 4380 | Y |
| 60 | 深圳市政府 | https://weibo.com/shenzhenfabu | 2422890 | 1013 | 87698 | Y |
| 61 | 深圳市职业病防治院 | https://weibo.com/SZZYBFZY | 565 | 228 | 499 | Y |
| 62 | 深圳市住房保障署长圳项目建设指挥部 | <https://weibo.com/szzfbzs> | 1413 | 10 | 237 | Y |
| 63 | 深圳市住建局 | https://weibo.com/u/2892809924 | 116122 | 196 | 2706 | Y |
| 64 | 深圳壹基金公益基金会 | https://weibo.com/yijijin | 2197706 | 1379 | 11338 | Y |
| 65 | 天虹股份 | <https://weibo.com/rainbowcn> | 145587 | 740 | 9163 | Y |
| 66 | 盐田区公安分局 | https://weibo.com/u/2839369142 | 3969 | 119 | 4152 | Y |
| 67 | 盐田区交警大队 | https://weibo.com/u/2673690433 | 14846 | 633 | 44644 | Y |
| 68 | 盐田区政府 | <https://weibo.com/220244094> | 17611 | 198 | 11595 | Y |
| 69 | 中国人民财产保险股份有限公司深圳市分公司 | <https://weibo.com/szepicc> | 1811 | 141 | 1887 | Y |
| 70 | 中国铁路广州局集团有限公司 | <https://weibo.com/gztielu> | 1687457 | 1726 | 22559 | Y |
| 71 | 驻港部队（深圳基地） | https://weibo.com/u/1181382331 | 1161876 | 75 | 467 | Y |
| 72 | 宝安二医（集团）二总院（松岗人民医院） | https://weibo.com/u/3516720905 | 333 | 153 | 59 | N |
| 73 | 宝安区滨海小学 | https://weibo.com/u/2819067022 | 145 | 57 | 155 | N |
| 74 | 宝安区经济促进局 | https://weibo.com/u/5127734989 | 41 | 21 | 37 | N |
| 75 | 宝安区中心血站 | <https://weibo.com/u/1373203335> | 199 | 12 | 810 | N |
| 76 | 福田区福保街道党工委 | https://weibo.com/u/2862816881 | 38 | 27 | 9 | N |
| 77 | 福田区福田街道党工委 | <https://weibo.com/u/2889097351> | 64 | 145 | 1794 | N |
| 78 | 福田区华强北街道党工委 | <https://weibo.com/u/2868061795> | 2930 | 75 | 139 | N |
| 79 | 光明区凤凰街道塘家社区党群服务中心 | <https://weibo.com/tjsqfwzx> | 238 | 97 | 464 | N |
| 80 | 国家水利部 | <https://weibo.com/iwater0322> | 845 | 78 | 272 | N |
| 81 | 龙华街道松和小学 | <https://weibo.com/szlhshxx> | 1096 | 9 | 15 | N |
| 82 | 美丽深圳公益基金会 | https://weibo.com/u/6211279041 | 2556 | 92 | 55 | N |
| 83 | 南山二外（集团）海德学校 | <https://weibo.com/u/5320633903> | 126 | 0 | 0 | N |
| 84 | 南山区南风社会工作服务社 | <https://weibo.com/sznanfeng> | 143 | 186 | 111 | N |
| 85 | 南山区三防办 | <https://weibo.com/u/6297347610> | 22398 | 149 | 952 | N |
| 86 | 坪山区实验学校 | <https://weibo.com/u/5673491782> | 290 | 81 | 137 | N |
| 87 | 深圳安琪永记食品有限公司 | https://weibo.com/u/7414575775 | 2 | 65 | 0 | N |
| 88 | 深圳市（光明区）实验学校 | <https://weibo.com/szsy2014> | 1330 | 128 | 75 | N |
| 89 | 深圳市公园管理中心 | https://weibo.com/szparks | 44330 | 69 | 4860 | N |
| 90 | 深圳市观澜第二中学 | https://weibo.com/u/5175970920 | 193 | 34 | 56 | N |
| 91 | 深圳市海王星辰健康药房连锁有限公司 | https://weibo.com/nepstar628 | 44246 | 235 | 1735 | N |
| 92 | 深圳市民治中学 | https://weibo.com/u/5182077472 | 68 | 12 | 83 | N |
| 93 | 深圳市人民医院 | https://weibo.com/szhospital | 876 | 101 | 112 | N |
| 94 | 深圳市社会工作者协会 | https://weibo.com/szswa | 2336 | 118 | 85 | N |
| 95 | 深圳市卫生监督局 | https://weibo.com/szwsjd | 14968 | 266 | 3787 | N |
| 96 | 深圳市义工联 | <https://weibo.com/u/2534521444> | 4650 | 222 | 645 | N |
| 97 | 深圳市中西医结合医院（原沙井人民医院） | https://weibo.com/u/3546344640 | 179 | 42 | 7 | N |
| 98 | 香港大学深圳医院 | https://weibo.com/hkuszh | 13131 | 62 | 1488 | N |
| 99 | 香港中华电力 | https://weibo.com/theclpgroup | 130 | 8 | 10 | N |
| 100 | 中国一治深康学校工程部 | <https://weibo.com/u/3297854150> | 44 | 138 | 71 | N |

**Organizational actors identified from Hong Kong on Twitter**

| No | Organization | Twitter account | No. of followers | No. of followees | No. of tweets | Tweets during the time frame |
| --- | --- | --- | --- | --- | --- | --- |
| 1 | Hong Kong Exchanges and Clearing Limited | https://twitter.com/HKEXGroup | 397 | 9197 | 6662 | Y |
| 2 | MTR Corporation Limited | https://twitter.com/mtrupdate | 4 | 101800 | 23500 | Y |
| 3 | Cathay Pacific Airways Limited | https://twitter.com/cathaypacific | 2899 | 561700 | 37500 | Y |
| 4 | Harbour City | https://twitter.com/hkharbourcity | 732 | 7124 | 13100 | Y |
| 5 | Green Sense | https://twitter.com/hkgreensense | 140 | 505 | 1504 | Y |
| 6 | The Hongkong and Shanghai Banking Corporation Limited | https://twitter.com/HSBC_HK | 41 | 4346 | 13800 | Y |
| 7 | Lingnan University | https://twitter.com/lingnanuni | 94 | 416 | 659 | Y |
| 8 | Greenpeace Hong Kong | https://twitter.com/greenpeace_hk | 432 | 4185 | 3128 | Y |
| 9 | The Hong Kong Jockey Club | https://twitter.com/HKJC_Racing | 196 | 149800 | 12200 | Y |
| 10 | CBRE Hong Kong | https://twitter.com/CBREHongKong | 611 | 2943 | 4474 | Y |
| 11 | World Wide Fund for Nature Hong Kong | https://twitter.com/wwfhk | 272 | 75700 | 2290 | Y |
| 12 | Invest Hong Kong | https://twitter.com/InvestHK | 312 | 13100 | 3476 | Y |
| 13 | City University of Hong Kong | https://twitter.com/CityUHongKong | 22 | 3697 | 1077 | Y |
| 14 | The University of Hong Kong | https://twitter.com/HKUniversity | 1269 | 12100 | 3065 | Y |
| 15 | Hong Kong Golf Association | https://twitter.com/HongKongGolf | 135 | 884 | 2080 | Y |
| 16 | The Hong Kong Golf Club | https://twitter.com/hkgolfclub | 271 | 721 | 846 | Y |
| 17 | The Hong Kong International Film Festival Society | https://twitter.com/HKIFFS | 70 | 45200 | 1390 | Y |
| 18 | Ocean Park HK | https://twitter.com/OCEAN_PARK | 0 | 957 | 1598 | Y |
| 19 | Hong Kong Airlines | https://twitter.com/hkairlines | 143 | 5594 | 989 | Y |
| 20 | Airport Authority Hong Kong | https://twitter.com/hkairport | 24 | 75600 | 947 | Y |
| 21 | Fintech Association of Hong Kong | https://twitter.com/hongkongfintech | 375 | 6888 | 2438 | Y |
| 22 | Hong Kong Baptist University | https://twitter.com/hkbaptistu | 2669 | 1227 | 1031 | Y |
| 23 | The Hong Kong Polytechnic University | https://twitter.com/HongKongPolyU | 372 | 16900 | 6132 | Y |
| 24 | Hong Kong UNESCO Global Geopark | https://twitter.com/HKgeopark | 128 | 378 | 1134 | Y |
| 25 | Hong Kong Tourism Board | https://twitter.com/discoverhk | 95 | 663000 | 5555 | Y |
| 26 | The Hong Kong Trade Development Council | https://twitter.com/hktdc | 3686 | 15600 | 17300 | Y |
| 27 | Lifehouse Hong Kong | https://twitter.com/LifehouseHK | 13 | 222 | 657 | Y |
| 28 | Cyberport | https://twitter.com/cyberport_hk | 1693 | 4050 | 5548 | Y |
| 29 | Four Seasons HK | https://twitter.com/FSHongKong | 1225 | 38300 | 8587 | Y |
| 30 | Hong Kong Observatory | https://twitter.com/HKObservatory | 2 | 125700 | 17000 | Y |
| 31 | Mandarin Oriental HK | https://twitter.com/MO_HKG | 2094 | 25800 | 15300 | Y |
| 32 | HKSAR Government | https://twitter.com/newsgovhk | 66 | 26200 | 16900 | Y |
| 33 | Hong Kong Confederation of Trade Unions | https://twitter.com/HongKongCTU | 318 | 1293 | 808 | Y |
| 34 | The Chinese University of Hong Kong | https://twitter.com/CUHKofficial | 526 | 7134 | 299 | Y |
| 35 | Asia Society Hong Kong Center | https://twitter.com/AsiaSocietyHK | 674 | 2230 | 2752 | Y |
| 36 | Oxfam Hong Kong | https://twitter.com/oxfamhongkong | 45 | 3499 | 2766 | Y |
| 37 | Feeding Hong Kong | https://twitter.com/FeedingHK | 264 | 1346 | 2035 | Y |
| 38 | Information Services Department | https://twitter.com/newsgovhk_epr | 3 | 7080 | 67800 | Y |
| 39 | V Cycle | https://twitter.com/VCYCLEHK | 189 | 23 | 2 | N |
| 40 | Cathay Dragon | https://twitter.com/dragonair | 78 | 3860 | 821 | N |
| 41 | Home Affairs Bureau | https://twitter.com/HKSAR_HAB | 0 | 5 | 0 | N |
| 42 | Hong Kong Police Force | https://twitter.com/hkpoliceforce | 7 | 86800 | 2677 | N |
| 43 | Hong Kong Federation of Youth Groups Lee Shau Kee Primary School | https://twitter.com/lskps | 26 | 37 | 819 | N |
