# Supplementary material for: Longitudinal multiplexity and structural constraints of online emergency collaborative networks: A tale of two Chinese societies
Source: PLoS One. 2023 Jul 27;18(7):e0289277. doi: 10.1371/journal.pone.0289277 (PMC10374111; doi:10.1371/journal.pone.0289277)
Supplement: S4 Table — (DOCX) [file pone.0289277.s004.docx]

**Weibo hashtags identified from tweets posted by Shenzhen-based organizational actors**

| No | Hashtag | No | Hashtag | No | Hashtag | No | Hashtag |
| --- | --- | --- | --- | --- | --- | --- | --- |
| 1 | #国庆小长假路况播报# | 186 | #国庆假期路况提示# | 371 | #女子乘高铁脏话连篇# | 556 | #晚安，盐田# |
| 2 | #《地铁早班车》# | 187 | #国庆假期路况续报# | 372 | #碰瓷保时捷被群众抬走# | 557 | #晚安深圳# |
| 3 | #00后入学新三样# | 188 | #国庆假期我在岗# | 373 | #辟谣# | 558 | #晚安小赫兹# |
| 4 | #0910教师节# | 189 | #国庆节# | 374 | #平安365# | 559 | #晚高峰路况播报# |
| 5 | #12日夜间沿海有7-8级阵风# | 190 | #国庆节猎虎# | 375 | #平安产险“平安树”亮相2018“新博会”# | 560 | #晚会彩排交通保障# |
| 6 | #12日夜间沿海有8级阵风# | 191 | #国庆路况播报# | 376 | #平安出行，交警同行# | 561 | #晚间锻炼# |
| 7 | #15分钟制造一袋垃圾# | 192 | #国庆排队照# | 377 | #平安开学路# | 562 | #晚间分享# |
| 8 | #2018东博会# | 193 | #国庆期间路况播报# | 378 | #平安校园行# | 563 | #万能的消防员# |
| 9 | #2018国庆节# | 194 | #国庆期间路况提示# | 379 | #平安盐田 你我共建# | 564 | #王俊凯0921生日快乐# |
| 10 | #2018抗击双台风# | 195 | #国庆去哪儿# | 380 | #坪山资讯# | 565 | #网民节# |
| 11 | #2018雷霆行动# | 196 | #国庆首日高速路况播报# | 381 | #七重品质保证尽在一个天虹# | 566 | #微博查违法# |
| 12 | #2018年烈士纪念日# | 197 | #国庆我在岗# | 382 | #奇妙的石头# | 567 | #微博辟谣# |
| 13 | #2018年深圳市公交出行宣传周# | 198 | #国庆长假深圳路况# | 383 | #签到领红包# | 568 | #微电影小团圆# |
| 14 | #2018年中秋国庆消费提示# | 199 | #孩子提醒舅舅送错幼儿园# | 384 | #前车之鉴# | 569 | #微感动# |
| 15 | #2018深圳物博会# | 200 | #海铁发布# | 385 | #前海# | 570 | #微公告# |
| 16 | #2018中非合作论坛# | 201 | #海洋天堂# | 386 | #抢修路上# | 571 | #微警示# |
| 17 | #2100万人宅出新境界# | 202 | #寒露# | 387 | #亲子学堂# | 572 | #微警事# |
| 18 | #60万医学生仅10万从医# | 203 | #行人非机动车整治# | 388 | #轻松筹4周年# | 573 | #微镜头# |
| 19 | #6日起降雨逐日增多# | 204 | #航班信息# | 389 | #轻松时刻# | 574 | #微辟谣# |
| 20 | #80后电网技术狂人# | 205 | #航拍晚高峰# | 390 | #轻松一刻# | 575 | #微曝光# |
| 21 | #9.22世界无车日# | 206 | #黑臭水体治理攻坚战# | 391 | #情况通报# | 576 | #微视频# |
| 22 | #920爱牙日# | 207 | #很高兴认识你邻居# | 392 | #请民寻人# | 577 | #微讨论# |
| 23 | #920全国爱牙日# | 208 | #虎门大桥路况# | 393 | #请让道# | 578 | #微提醒# |
| 24 | #95公益周# | 209 | #华润万家&伊利“臻活力”全民健康跑# | 394 | #请勿前往# | 579 | #微喔新品# |
| 25 | #99公益日# | 210 | #华润万家国庆朋友圈摄影大赛# | 395 | #庆祝改革开放40周年# | 580 | #微招聘# |
| 26 | #AI进电网# | 211 | #怀双胞胎基因# | 396 | #秋分# | 581 | #为爱同行# |
| 27 | #BD-53# | 212 | #欢度国庆# | 397 | #秋风舒缓起，露从今夜白# | 582 | #为爱一起捐# |
| 28 | #BD-54# | 213 | #环保水务舆情# | 398 | #秋意浓浓# | 583 | #为吃荤还是吃素起矛盾# |
| 29 | #BD-55# | 214 | #环保小百科# | 399 | #求职季# | 584 | #违法整治# |
| 30 | #BD行动# | 215 | #婚后发胖# | 400 | #区校动态# | 585 | #违停整治# |
| 31 | #MCF保育# | 216 | #活动预告# | 401 | #趣味文字# | 586 | #卫龙等辣条抽检不合格# |
| 32 | #MCF自然教育# | 217 | #活色广东# | 402 | #权威发布# | 587 | #卫龙辣条回应抽检# |
| 33 | #TNC陆地保护# | 218 | #火车上的故事# | 403 | #全国爱牙日# | 588 | #温暖龙岗# |
| 34 | #TNC在关注# | 219 | #货车整治# | 404 | #全国高血压日# | 589 | #温暖南山# |
| 35 | #XIN公益大会# | 220 | #吉林发布粉丝节# | 405 | #全力恢复交通# | 590 | #温暖坪山# |
| 36 | #阿尔茨海默病# | 221 | #记得看10月份工资条# | 406 | #全力迎战台风山竹# | 591 | #温暖中国# |
| 37 | #爱心随行# | 222 | #家校警护航# | 407 | #全球气候峰会# | 592 | #温馨提示# |
| 38 | #爱牙日# | 223 | #家校警交通安全护航队# | 408 | #热点关注# | 593 | #温馨提醒# |
| 39 | #安哥在行动# | 224 | #假期出行安全提示# | 409 | #热烈祝贺光明区揭牌# | 594 | #文化岭南# |
| 40 | #安全婆婆嘴# | 225 | #驾照考试科目一# | 410 | #人才公园灯光秀# | 595 | #文化南山# |
| 41 | #安全视频展播# | 226 | #捡拾中国# | 411 | #日行壹善# | 596 | #文明交通，你我同行# |
| 42 | #安全提示# | 227 | #见圾行事# | 412 | #如懿传# | 597 | #文明养犬# |
| 43 | #安全提示每日一图# | 228 | #剑指诈骗# | 413 | #如懿偷吃酸杏# | 598 | #我爱你中国# |
| 44 | #安全微课堂# | 229 | #健康科普# | 414 | #三微大赛# | 599 | #我们的开学季# |
| 45 | #安全小手册# | 230 | #健康南山# | 415 | #扫黑除恶 福田在行动# | 600 | #我们都是捡星人# |
| 46 | #安全用电温馨提示# | 231 | #健康深圳# | 416 | #扫黑除恶# | 601 | #我与中国铁路40年# |
| 47 | #把20米长的扶梯当滑梯# | 232 | #健康小知识# | 417 | #扫黑除恶，罗湖在行动# | 602 | #我在岗位上# |
| 48 | #霸座男限乘所有火车# | 233 | #健康养生# | 418 | #扫黑除恶进行时# | 603 | #梧桐动态# |
| 49 | #白露# | 234 | #僵尸车整治# | 419 | #扫黑除恶深圳在行动# | 604 | #五进送平安# |
| 50 | #百城销枪保民安# | 235 | #交警动态# | 420 | #扫黑除恶在行动# | 605 | #五进走访送平安# |
| 51 | #宝安# | 236 | #交警队喝茶# | 421 | #扫黑除恶中央督导在广东# | 606 | #五仁月饼翻身# |
| 52 | #宝安天虹购物中心# | 237 | #交警风采# | 422 | #森林防火# | 607 | #午饭时间# |
| 53 | #宝安中医院# | 238 | #交警蜀黍来提醒# | 423 | #山竹# | 608 | #午间分享# |
| 54 | #报警录音 暴雨梨花式# | 239 | #交警说法# | 424 | #山竹”威力仍未减低，爱心永在路上# | 609 | #夏秋季攻势# |
| 55 | #被看见 才安全# | 240 | #交警一线# | 425 | #山竹疯了别出门# | 610 | #仙湖植物园# |
| 56 | #比出我的中国心# | 241 | #交警正能量# | 426 | #山竹过后# | 611 | #现实版红岸基地# |
| 57 | #比心中国# | 242 | #交警重要信息# | 427 | #山竹过后龙岗路况# | 612 | #宪法进万家# |
| 58 | #不到一分钟温暖一车人# | 243 | #交通安全体验课# | 428 | #山竹过后深圳路况# | 613 | #相信小的伟大# |
| 59 | #不凡四十年# | 244 | #交通安全宣传# | 429 | #山竹回顾# | 614 | #消防员连背带抱救人# |
| 60 | #不想上班# | 245 | #交通安全宣传进校园# | 430 | #山竹距离我市129公里# | 615 | #小编在现场# |
| 61 | #不止是李健演唱会# | 246 | #交通百科# | 431 | #山竹距离我市184公里# | 616 | #小翠儿的多彩空间# |
| 62 | #步步为爱，益起行善# | 247 | #交通管制# | 432 | #山竹来了# | 617 | #小孩连续按停2个电梯# |
| 63 | #厕所革命# | 248 | #交通规划建设进展# | 433 | #山竹来袭 深圳在行动# | 618 | #小赫兹# |
| 64 | #畅行中国 交警同行# | 249 | #交通行业圳能量# | 434 | #山竹来袭，龙华公安在行动# | 619 | #小赫兹爱心漂流# |
| 65 | #畅行中国，交警同行# | 250 | #交通路况# | 435 | #山竹台风来袭，深圳交警在行动# | 620 | #小赫兹无处不在# |
| 66 | #倡议近期绿色出行# | 251 | #交通事故定责动画# | 436 | #山竹袭来警徽闪耀# | 621 | #小黑颈鹤# |
| 67 | #超级警情# | 252 | #交通事故警示# | 437 | #陕西宁强5.3级地震# | 622 | #小朋友你多大啦# |
| 68 | #超级晚高峰# | 253 | #交通提示# | 438 | #汕头暴雨# | 623 | #小天生活# |
| 69 | #超强台风“山竹”正在以22km/h靠近广东并且将在周日给我们带来风雨影响而正因为刚好是在周日所以“山竹”不是个好台风# | 254 | #交通违法曝光台# | 439 | #身边暖警# | 624 | #校园风景线# |
| 70 | #超强台风山竹# | 255 | #交通整治直击# | 440 | #深夜放毒# | 625 | #心理健康# |
| 71 | #城管在行动# | 256 | #教师茶座# | 441 | #深圳40周年灯光秀# | 626 | #心意团圆计划# |
| 72 | #城管正能量# | 257 | #教师节# | 442 | #深圳爱卫# | 627 | #新妈妈抱宝宝的姿势不对影响体型# |
| 73 | #城市灯光# | 258 | #教师节快乐# | 443 | #深圳暴雨# | 628 | #新时代·幸福美丽新边疆# |
| 74 | #吃大闸蟹的季节# | 259 | #街区动态# | 444 | #深圳出行小贴士# | 629 | #新时代•铁路榜样# |
| 75 | #持站票男子霸占行李架# | 260 | #街区新闻# | 445 | #深圳大小事# | 630 | #新时代亮生活# |
| 76 | #出行提示# | 261 | #节日我在岗# | 446 | #深圳灯光秀# | 631 | #新时代幸福美丽新边疆# |
| 77 | #传承英烈志·建功新时代# | 262 | #结核病筛查误用卡介苗# | 447 | #深圳电价五连降# | 632 | #星光公益力# |
| 78 | #创建国家森林城市 打造世界著名花城# | 263 | #姐姐上网搜“怎么办”# | 448 | #深圳公安国庆安保掠影# | 633 | #星期三查餐厅# |
| 79 | #创建国家森林城市# | 264 | #今日天气# | 449 | #深圳公共安全指数# | 634 | #幸福南山# |
| 80 | #慈展会# | 265 | #今天吃什么# | 450 | #深圳国际BT领袖峰会# | 635 | #炫富挑战# |
| 81 | #从新生儿到100岁老人的面孔# | 266 | #今天寒露# | 451 | #深圳航空航线# | 636 | #学前教育# |
| 82 | #打造全国最干净城市# | 267 | #津警说# | 452 | #深圳好心人# | 637 | #寻人启事# |
| 83 | #打造世界著名花城# | 268 | #紧急救护# | 453 | #深圳花事# | 638 | #盐田电动自行车燃烧实验# |
| 84 | #大海不能没有你# | 269 | #紧急通告# | 454 | #深圳稽查多次违法车# | 639 | #盐田交警“猎虎”整治行动# |
| 85 | #大美南山# | 270 | #禁摩限电# | 455 | #深圳交警猎虎# | 640 | #盐田交警温馨提示# |
| 86 | #大数据查违法# | 271 | #京东到家1020宅购节# | 456 | #深圳交警提示# | 641 | #盐田交警在行动# |
| 87 | #带你认识深圳# | 272 | #惊艳中国# | 457 | #深圳交警铁骑# | 642 | #盐田警察在现场# |
| 88 | #带着微博去吉林# | 273 | #精彩南山# | 458 | #深圳交警温馨提示# | 643 | #演唱会交通保障# |
| 89 | #带着微博去旅行# | 274 | #精彩预告# | 459 | #深圳交警一线# | 644 | #演出# |
| 90 | #滴滴回应乘客喝到尿# | 275 | #精神卫生日# | 460 | #深圳交警直播# | 645 | #央视新闻微直播# |
| 91 | #地球卫士奖# | 276 | #警察小提示# | 461 | #深圳交通政策# | 646 | #阳春水灾# |
| 92 | #地铁抗风行动# | 277 | #警官微课堂# | 462 | #深圳交通执法# | 647 | #姚景元0913生日快乐# |
| 93 | #地铁早班车# | 278 | #警徽荣耀# | 463 | #深圳禁摩限电# | 648 | #野保动态# |
| 94 | #第23号台风百里嘉# | 279 | #警徽荣耀，共铸平安# | 464 | #深圳警讯# | 649 | #夜间行车秘籍# |
| 95 | #第六届中国慈展会# | 280 | #警情通报# | 465 | #深圳九价HPV疫苗# | 650 | #一路平安# |
| 96 | #第四届深圳国际创客周# | 281 | #警犬执勤累倒# | 466 | #深圳坪山交警# | 651 | #一年违章15次被退婚# |
| 97 | #点赞公大70年# | 282 | #警营正能量# | 467 | #深圳身边事# | 652 | #一起过国庆# |
| 98 | #点赞节日在岗好警察# | 283 | #净水计划# | 468 | #深圳生活# | 653 | #一起过中秋节# |
| 99 | #点赞我的祖国# | 284 | #净网2018# | 469 | #深圳市民中心灯光秀# | 654 | #一线微观# |
| 100 | #电动汽车任我行# | 285 | #敬畏自然# | 470 | #深圳天气# | 655 | #一直播# |
| 101 | #电亮民族美# | 286 | #九一八事变# | 471 | #深圳文艺名家# | 656 | #医院纪事# |
| 102 | #电亮微课堂# | 287 | #救在壹线# | 472 | #深圳新闻# | 657 | #以案析法# |
| 103 | #电亮责任每一度# | 288 | #居家用电# | 473 | #深圳严查不系安全带# | 658 | #艺述福田# |
| 104 | #电网保驾3200公里穿越南北电动行# | 289 | #具字里面到底几横# | 474 | #深圳严查多次违法车# | 659 | #印尼亚运会# |
| 105 | #雕刻月光# | 290 | #距下个长假还有119天# | 475 | #深圳严查酒驾# | 660 | #迎战强台风“山竹”，广东出行大家帮# |
| 106 | #斗破苍穹# | 291 | #聚焦党代会 共建新光明# | 476 | #深圳严查违停# | 661 | #尤长靖0919生日快乐# |
| 107 | #对邪教说不# | 292 | #聚焦光明“两会”# | 477 | #深圳最美的厕所墙# | 662 | #有“福”同享# |
| 108 | #峨眉山跳崖女孩# | 293 | #崛起教育集团# | 478 | #深圳最美灯光秀# | 663 | #有奖征集# |
| 109 | #发现深圳# | 294 | #军人探亲路上救人# | 479 | #神奇保护侠# | 664 | #又到退伍季# |
| 110 | #发现最美铁路# | 295 | #卡口整治行动# | 480 | #生活在此，爱在此# | 665 | #舆情反馈# |
| 111 | #法治南山# | 296 | #开饭啦# | 481 | #生活在深圳# | 666 | #遇见最美劳动者# |
| 112 | #翻身实验学校# | 297 | #开学# | 482 | #施工路况提示# | 667 | #粤创粤新# |
| 113 | #反诈骗进校园# | 298 | #开学典礼# | 483 | #湿地直通车# | 668 | #粤建粤美# |
| 114 | #饭前喝汤,苗条健康;饭后喝汤,越喝越胖# | 299 | #开学季# | 484 | #十点在广州十点在香港# | 669 | #粤来粤好# |
| 115 | #防骗微宣传# | 300 | #看灯光秀今天央视最好# | 485 | #十一平安行# | 670 | #粤事大家谈# |
| 116 | #防台风常识紧急提示# | 301 | #抗击暴雨# | 486 | #实时路况# | 671 | #粤有我粤畅行# |
| 117 | #风王山竹来袭# | 302 | #抗击台风# | 487 | #实用贴# | 672 | #云南普洱墨江5.9级地震# |
| 118 | #扶贫路上# | 303 | #抗击台风山竹# | 488 | #实用小百科# | 673 | #在线访谈# |
| 119 | #服务直通车# | 304 | #抗台风我们在行动# | 489 | #食品安全# | 674 | #臧天朔去世# |
| 120 | #福田好少年# | 305 | #狂风暴雨仍将持续# | 490 | #世界阿尔兹海默症日# | 675 | #早安 深圳# |
| 121 | #福田民生# | 306 | #昆山事件属正当防卫# | 491 | #世界避孕日# | 676 | #早安# |
| 122 | #福田天气# | 307 | #垃圾分类# | 492 | #世界精神卫生日# | 677 | #早安，龙岗# |
| 123 | #福田英语角# | 308 | #来撩天# | 493 | #世界清洁日# | 678 | #早安，你好# |
| 124 | #福田招聘# | 309 | #来自网友的温馨提示# | 494 | #事故微警示# | 679 | #早安，深圳# |
| 125 | #福田正能量# | 310 | #懒婚# | 495 | #视频连连看# | 680 | #早安坪山# |
| 126 | #福田资讯# | 311 | #雷电防御# | 496 | #视知车学院# | 681 | #早安深圳# |
| 127 | #覆车之戒# | 312 | #类雌激素物质# | 497 | #室内空气质量检测# | 682 | #早安心语# |
| 128 | #改革开放40年# | 313 | #历史上de今天# | 498 | #首个自主研发抗癌新药获批上市# | 683 | #早高峰路况播报# |
| 129 | #改革开放40周年# | 314 | #厉害了我的宝企# | 499 | #蜀黍帮寻人# | 684 | #早高峰路况续报# |
| 130 | #感恩老师# | 315 | #两萌娃国庆景区捡垃圾# | 500 | #蜀黍带你涨姿势# | 685 | #长车资讯# |
| 131 | #高速行车宝典# | 316 | #列车上的中秋# | 501 | #数字城管# | 686 | #涨姿势# |
| 132 | #高校之窗# | 317 | #烈士纪念日# | 502 | #双V看盛会# | 687 | #找到你# |
| 133 | #各地交管见闻# | 318 | #凌晨平安# | 503 | #随手拍# | 688 | #圳警说# |
| 134 | #给上司留遗书后失联# | 319 | #凌晨平安整治# | 504 | #台风# | 689 | #正能量# |
| 135 | #公安交管放管服改革# | 320 | #零废弃# | 505 | #台风“山竹”# | 690 | #执法直播台# |
| 136 | #公安民警三微大赛# | 321 | #领航中国# | 506 | #台风“山竹”来袭# | 691 | #直击一线# |
| 137 | #公告公示# | 322 | #刘昊然1010生日快乐# | 507 | #台风“山竹”袭来# | 692 | #植物小讲堂# |
| 138 | #公交出行宣传周及无车日# | 323 | #龙岗一宗死亡交通事故# | 508 | #台风“潭美”# | 693 | #治水提质# |
| 139 | #公交人的小团圆# | 324 | #龙华发现# | 509 | #台风百里嘉# | 694 | #致敬40年# |
| 140 | #公益救援在行动# | 325 | #龙华分享# | 510 | #台风动态# | 695 | #致敬公安英烈# |
| 141 | #公园动态# | 326 | #龙华关注# | 511 | #台风防御# | 696 | #致敬公安英雄# |
| 142 | #鼓励正当防卫# | 327 | #龙华扫黑除恶# | 512 | #台风过后# | 697 | #中国好网民# |
| 143 | #关爱环卫工# | 328 | #龙华提醒# | 513 | #台风红色+暴雨红色预警信号生效中# | 698 | #中国平安品牌大使李健# |
| 144 | #关于台风“山竹”期间全市营业厅暂停营业的通告# | 329 | #龙华通报# | 514 | #台风红色预警# | 699 | #中国人的故事# |
| 145 | #冠心病# | 330 | #龙华新闻# | 515 | #台风红色预警信号生效中# | 700 | #中国人为什么爱喝热水？# |
| 146 | #光明科学城# | 331 | #龙华祝福# | 516 | #台风后# | 701 | #中国速度 领跑未来# |
| 147 | #广东分享# | 332 | #路况播报# | 517 | #台风后返工# | 702 | #中国速度领跑世界# |
| 148 | #广东关注# | 333 | #路况提示# | 518 | #台风来了# | 703 | #中国速度领跑未来# |
| 149 | #广东好人# | 334 | #路况续报# | 519 | #台风来袭 深圳在行动# | 704 | #中国我爱你# |
| 150 | #广东交通# | 335 | #罗湖交警在行动# | 520 | #台风路况播报# | 705 | #中国有我# |
| 151 | #广东骄傲# | 336 | #罗湖区人民医院# | 521 | #台风山竹# | 706 | #中华老字号集体卖萌# |
| 152 | #广东教育# | 337 | #罗湖社区家园报# | 522 | #台风山竹来袭# | 707 | #中秋# |
| 153 | #广东经济# | 338 | #绿色出行# | 523 | #台风山竹袭来# | 708 | #中秋佳节# |
| 154 | #广东民生# | 339 | #妈妈给装的行李箱# | 524 | #台风山竹袭深# | 709 | #中秋佳节舍团圆 民警执勤保平安# |
| 155 | #广东汕头暴雨# | 340 | #卖萌日# | 525 | #台风山竹影响深圳# | 710 | #中秋佳节我在岗# |
| 156 | #广东史上最大规模台风预警# | 341 | #没有感情的广东人# | 526 | #台风时刻的福田正能量# | 711 | #中秋假期路况播报# |
| 157 | #广东双台风# | 342 | #每日精选# | 527 | #台风潭美# | 712 | #中秋假期温馨提示# |
| 158 | #广东提醒# | 343 | #每日警星# | 528 | #台风天气，注意事项# | 713 | #中秋假期我在岗# |
| 159 | #广东天气# | 344 | #美好在发声# | 529 | #台风营业通知# | 714 | #中秋节# |
| 160 | #广东消防抗击台风山竹# | 345 | #美丽深圳志愿者在行动# | 530 | #探寻深圳警队20最# | 715 | #中秋节快乐# |
| 161 | #广东正能量# | 346 | #美丽中国行动者# | 531 | #特别感谢# | 716 | #中秋节猎虎# |
| 162 | #广东政闻# | 347 | #美在广东# | 532 | #提示# | 717 | #中秋快乐# |
| 163 | #广东政务# | 348 | #梦想飞屋第3站# | 533 | #提醒# | 718 | #中秋路况# |
| 164 | #广而告之# | 349 | #梦想飞屋第3站：贵州省安顺市镇宁县本寨镇鱼凹小学# | 534 | #天虹梦想飞屋在行动# | 719 | #中秋路况提示# |
| 165 | #广深港高铁# | 350 | #猕猴桃混进了企鹅群# | 535 | #天虹情报站# | 720 | #中秋天气TIPS# |
| 166 | #广式生活# | 351 | #米脂故意杀人案凶手被执行死刑# | 536 | #天津交警# | 721 | #中秋外围交通# |
| 167 | #广铁出行微提示# | 352 | #明智饮酒 拒绝酒驾# | 537 | #天气提示# | 722 | #中秋我在岗# |
| 168 | #广铁故事# | 353 | #墨江5.9级地震# | 538 | #天天剧场# | 723 | #中秋夜上半夜可见月亮# |
| 169 | #广铁人的故事# | 354 | #那年今日# | 539 | #天天漫画# | 724 | #中秋夜严查酒驾，蜀黍请你吃饼赏月# |
| 170 | #广铁资讯# | 355 | #南山常兴天虹# | 540 | #天天生活# | 725 | #众志成城抗击山竹# |
| 171 | #广站资讯# | 356 | #南山福来day# | 541 | #调查# | 726 | #众志成城抗山竹# |
| 172 | #国际卖萌日# | 357 | #南山交通# | 542 | #铁路播报# | 727 | #重要提示# |
| 173 | #国际女童日# | 358 | #南山路况播报# | 543 | #投票啦# | 728 | #重要提醒# |
| 174 | #国际雪豹保护大会# | 359 | #南山扫黑除恶# | 544 | #投诉回音# | 729 | #重要信息# |
| 175 | #国庆# | 360 | #南山戏剧节# | 545 | #突发# | 730 | #周末路况播报# |
| 176 | #国庆168小时# | 361 | #南山新闻# | 546 | #突发路况提示# | 731 | #祝老师节日快乐# |
| 177 | #国庆7天# | 362 | #南山义工# | 547 | #拖欠农民工工资黑名单# | 732 | #筑梦大秦重载# |
| 178 | #国庆安保·我在岗上# | 363 | #南网快讯# | 548 | #脱贫攻坚在行动# | 733 | #壮阔东方潮 奋进新时代# |
| 179 | #国庆出游，用行为表达爱国# | 364 | #南网手账# | 549 | #外围高速路况# | 734 | #走进西电东送+寻美# |
| 180 | #国庆第七天，深圳交警带您空中看交通# | 365 | #泥头车整治# | 550 | #弯腰的国旗护卫队战士找到了# | 735 | #祖国有你让我很安心!# |
| 181 | #国庆第七天深圳交警带您空中看交通# | 366 | #泥土车整治# | 551 | #晚安 深圳# | 736 | #最安全# |
| 182 | #国庆第三天# | 367 | #你好，警察大学# | 552 | #晚安# | 737 | #最虹星期三# |
| 183 | #国庆高速路况# | 368 | #年度教师# | 553 | #晚安，光明# | 738 | #最美新娘# |
| 184 | #国庆假期高速路况播报# | 369 | #暖心一刻# | 554 | #晚安，龙岗# | 739 | #最新虎门大桥路况# |
| 185 | #国庆假期路况播报# | 370 | #女警出更# | 555 | #晚安，深圳# | 740 | #醉驾对特警称是自己人# |

**Twitter hashtags identified from tweets posted by Hong Kong-based organizational actors**

| No | Hashtag | No | Hashtag | No | Hashtag | No | Hashtag |
| --- | --- | --- | --- | --- | --- | --- | --- |
| 1 | #1o5c | 312 | #ecoparquetalice | 623 | #kix | 934 | #skies |
| 2 | #2018edxprize | 313 | #ecotech | 624 | #knowledgetransfer | 935 | #skiing |
| 3 | #2ndedition | 314 | #eday | 625 | #kol | 936 | #skyline |
| 4 | #40anni | 315 | #editing | 626 | #kols | 937 | #skyporn |
| 5 | #4th | 316 | #edtech | 627 | #kowlooncity | 938 | #skyscraper |
| 6 | #4yoseries | 317 | #education | 628 | #kramervskramer | 939 | #slash |
| 7 | #5g | 318 | #eduhk | 629 | #labourdepartment | 940 | #smartcity |
| 8 | #7eleven | 319 | #edwardyau | 630 | #ladym | 941 | #smarterbusiness |
| 9 | #academy | 320 | #elearning | 631 | #lafamille | 942 | #smartlearning |
| 10 | #academyaward | 321 | #electricity | 632 | #lake | 943 | #smartspace8 |
| 11 | #academyoffilm | 322 | #electronics | 633 | #laliga | 944 | #smes |
| 12 | #accenturefintechinnovationlabapac | 323 | #endcoal | 634 | #land | 945 | #snapshots |
| 13 | #acg2018 | 324 | #endoflifecare | 635 | #landconsultation | 946 | #socialenterprise |
| 14 | #achall | 325 | #endpoaching | 636 | #landsale | 947 | #socialgood |
| 15 | #achievements | 326 | #energy | 637 | #landscape | 948 | #socialmedia |
| 16 | #acs | 327 | #engineering | 638 | #language | 949 | #socialsector |
| 17 | #action | 328 | #entertainment | 639 | #lantau | 950 | #socialworkers |
| 18 | #admission | 329 | #entrepreneur | 640 | #launchevent | 951 | #societal |
| 19 | #admissionsseminars | 330 | #entrepreneurs | 641 | #lcx | 952 | #sofr |
| 20 | #advancedirectives | 331 | #entrepreneurship | 642 | #learncantonese | 953 | #software |
| 21 | #advertising | 332 | #environment | 643 | #lecafedejoelrobuchon | 954 | #solotravel |
| 22 | #aesthetics | 333 | #environmental | 644 | #lecture | 955 | #song |
| 23 | #aff | 334 | #erp | 645 | #letrou | 956 | #sourcing |
| 24 | #aff2021 | 335 | #esg | 646 | #letscleanthemup | 957 | #southchinasea |
| 25 | #africa | 336 | #esports | 647 | #lgbt | 958 | #southeastasian |
| 26 | #african | 337 | #esprit | 648 | #library | 959 | #souvenir |
| 27 | #afternoontea | 338 | #etf | 649 | #licensing | 960 | #spain |
| 28 | #ai | 339 | #etiquette | 650 | #lifehouse | 961 | #spirits |
| 29 | #air | 340 | #etp | 651 | #lifehousehk | 962 | #sports |
| 30 | #airbornetransmission | 341 | #eu | 652 | #lightrail | 963 | #sr15 |
| 31 | #airline | 342 | #euforia | 653 | #lightroomcc | 964 | #srise |
| 32 | #airportexpress | 343 | #eugene | 654 | #limits | 965 | #starbucks |
| 33 | #airports | 344 | #ev | 655 | #liquidity | 966 | #starferry |
| 34 | #airpurifier | 345 | #evolutionary | 656 | #listing | 967 | #startup |
| 35 | #airquality | 346 | #exchange | 657 | #listingrules | 968 | #startupexpress |
| 36 | #aisummit | 347 | #exhibition | 658 | #literaryfestival | 969 | #startups |
| 37 | #aisummithongkong | 348 | #explorehk | 659 | #literature | 970 | #stationery |
| 38 | #aliceandolivia | 349 | #explorehongkong | 660 | #liuqing | 971 | #staysocial |
| 39 | #almachk | 350 | #factfriday | 661 | #live | 972 | #steam |
| 40 | #almaty | 351 | #fairwood | 662 | #lladro | 973 | #stockmarket |
| 41 | #alumni | 352 | #familyoffices | 663 | #lng | 974 | #stopthetrade |
| 42 | #amanescaped | 353 | #fannyandalexander | 664 | #logistics | 975 | #storagedensity |
| 43 | #amazingpics | 354 | #fashion | 665 | #logon | 976 | #storm |
| 44 | #amazon | 355 | #fastfood | 666 | #lohas | 977 | #storms |
| 45 | #ambiente | 356 | #fasttrack | 667 | #losangeles | 978 | #strategy |
| 46 | #americanchemicalsociety | 357 | #fellowships | 668 | #loveandbullets | 979 | #straws |
| 47 | #andylau | 358 | #ferriswheel | 669 | #lowcarbon | 980 | #streaming |
| 48 | #animals | 359 | #ferzanözpetek | 670 | #luxembourg | 981 | #street |
| 49 | #anniversary | 360 | #ffanfanchuu | 671 | #luxurytravel | 982 | #streetphotography |
| 50 | #antibiotics | 361 | #fic | 672 | #machinery | 983 | #stress |
| 51 | #anxiety | 362 | #fidelity | 673 | #madonnadicampiglio | 984 | #stroke |
| 52 | #apac | 363 | #film | 674 | #madrid | 985 | #students |
| 53 | #apchau | 364 | #filmart | 675 | #magic | 986 | #studioexperience |
| 54 | #api | 365 | #films | 676 | #mainboard | 987 | #studyabroad |
| 55 | #apidays | 366 | #finalist | 677 | #mainland | 988 | #studyinhongkong |
| 56 | #aplasticocean | 367 | #finance | 678 | #mainlandchina | 989 | #studyoverseas |
| 57 | #app | 368 | #financialinclusion | 679 | #malaysia | 990 | #sugarfina |
| 58 | #apparelindustry | 369 | #financialrisk | 680 | #maldives | 991 | #sugarfinahk |
| 59 | #aprigf | 370 | #finedining | 681 | #management | 992 | #suitelife |
| 60 | #aprigf2020 | 371 | #fintech | 682 | #mangkhut | 993 | #suits |
| 61 | #architecture | 372 | #fintechhk | 683 | #map1 | 994 | #sun |
| 62 | #argentina | 373 | #fintechhotpot | 684 | #marcellofonte | 995 | #sunset |
| 63 | #armanibeauty | 374 | #fintechnations | 685 | #marine | 996 | #supertyphoonmangkhut |
| 64 | #armanibeautyhk | 375 | #fintechs | 686 | #marinelitter | 997 | #survey |
| 65 | #armanibox | 376 | #firedragondance | 687 | #maritime | 998 | #sustainability |
| 66 | #armaniboxhk | 377 | #fireservicesdepartment | 688 | #marketentry | 999 | #sustainable |
| 67 | #armchairtravel | 378 | #fireworks | 689 | #marketing | 1000 | #sustainablefashion |
| 68 | #art | 379 | #first | 690 | #markets | 1001 | #sustainablefinance |
| 69 | #artificialmicroswarm | 380 | #firstdayofschool | 691 | #master | 1002 | #sustainableseafood |
| 70 | #artist | 381 | #firstsemester | 692 | #matthewcheung | 1003 | #sustainableshuihau |
| 71 | #arts | 382 | #fixedincome | 693 | #media | 1004 | #sutardja |
| 72 | #artwork | 383 | #flashbackfriday | 694 | #medical | 1005 | #suzhou |
| 73 | #asean | 384 | #flooding | 695 | #medicalphysics | 1006 | #svennykvist |
| 74 | #ashk30 | 385 | #flores | 696 | #medicomtoy | 1007 | #taiwan |
| 75 | #ashkaai80d | 386 | #flowing | 697 | #medtech | 1008 | #taxation |
| 76 | #ashkaroundasiain80days | 387 | #flysfo | 698 | #meettheauthor | 1009 | #tbfbizfest18 |
| 77 | #asia | 388 | #food | 699 | #meitheal | 1010 | #teachers |
| 78 | #asiabriefinglive | 389 | #foodfuture | 700 | #mentalhealth | 1011 | #teaching |
| 79 | #asiagamechangers | 390 | #foodfuturesummit | 701 | #mentorship | 1012 | #teawg |
| 80 | #asian | 391 | #foodie | 702 | #metoo | 1013 | #tech |
| 81 | #asianamerican | 392 | #foodiehk | 703 | #mfs20 | 1014 | #techchallenge |
| 82 | #asianamericans | 393 | #foodtech | 704 | #mickey90 | 1015 | #technologies |
| 83 | #asianfinancialforum | 394 | #foodtechnology | 705 | #midautumnbunnyseries | 1016 | #technology |
| 84 | #asiangames2018 | 395 | #football | 706 | #midautumnfestival | 1017 | #tenoneteahouse |
| 85 | #asiapacific | 396 | #forbescloud100 | 707 | #midnightexpress | 1018 | #teresacheng |
| 86 | #asiaresearchpics | 397 | #fossilfuels | 708 | #milanfintechsummit | 1019 | #tgif |
| 87 | #asiasocietyhk | 398 | #free | 709 | #milestone | 1020 | #thailand |
| 88 | #asiasworldcity | 399 | #freshbeauty | 710 | #millennials | 1021 | #thecoffeeacademics |
| 89 | #assessment | 400 | #freshman | 711 | #mim | 1022 | #thenorthface |
| 90 | #asset | 401 | #friday | 712 | #minimalist | 1023 | #thepeak |
| 91 | #asthma | 402 | #frontline | 713 | #miriamyeung | 1024 | #therapy |
| 92 | #aswo | 403 | #fswellness | 714 | #mobile | 1025 | #theresnoplacelikehome |
| 93 | #atlantic | 404 | #fubiz | 715 | #mobility | 1026 | #thermaltumbler |
| 94 | #australia | 405 | #fullmoon | 716 | #mondayenergy | 1027 | #theshawshankredemption |
| 95 | #autumn | 406 | #funding | 717 | #monetaryauthority | 1028 | #thesilence |
| 96 | #autumnsonata | 407 | #fundraising | 718 | #monitoring | 1029 | #theunirankings |
| 97 | #avgeek | 408 | #fx | 719 | #moon | 1030 | #thisissustainable |
| 98 | #aviation | 409 | #gallery | 720 | #mooncake | 1031 | #throughaglassdarkly |
| 99 | #awards | 410 | #garygilchrist | 721 | #mooncakes | 1032 | #thunderstorm |
| 100 | #awesomehongkong | 411 | #gastronomy | 722 | #moonfestival | 1033 | #tierradelgeoparque |
| 101 | #b2b | 412 | #gba | 723 | #movie | 1034 | #tiffany |
| 102 | #backtoschool | 413 | #gbai | 724 | #moviequote | 1035 | #tiger |
| 103 | #bangladesh | 414 | #geography | 725 | #movies | 1036 | #time100 |
| 104 | #banking | 415 | #geoparkoftheday | 726 | #mpa | 1037 | #tips |
| 105 | #barriertrials | 416 | #geoparks | 727 | #mscbm | 1038 | #togetherhk |
| 106 | #bearbrick | 417 | #geopolitics | 728 | #msci | 1039 | #tokillamockingbird |
| 107 | #beauty | 418 | #georgia | 729 | #mtr | 1040 | #tokushima |
| 108 | #beijing | 419 | #germagic | 730 | #muscle | 1041 | #tokyo |
| 109 | #bekindtoyourmind | 420 | #germany | 731 | #music | 1042 | #tomford |
| 110 | #beltandroad | 421 | #ggn2018 | 732 | #musicbox | 1043 | #tomfordbeauty |
| 111 | #beltandroadsummit | 422 | #gigantoraptor | 733 | #musicians | 1044 | #top |
| 112 | #benchmark | 423 | #gigileung | 734 | #myanmar | 1045 | #top100 |
| 113 | #benovelty | 424 | #giorgiomoroder | 735 | #mywellfeeling | 1046 | #toronto |
| 114 | #berkeley | 425 | #givingback | 736 | #nagoya | 1047 | #tourism |
| 115 | #bestvantagepoint | 426 | #global | 737 | #nameofawoman | 1048 | #tours |
| 116 | #beyondtheheadlines | 427 | #globalfilm | 738 | #nanorobot | 1049 | #toys |
| 117 | #beyondthewall | 428 | #globalgeopark | 739 | #naplesinveils | 1050 | #tracychu |
| 118 | #bigdata | 429 | #globalmarketsincubator | 740 | #narita | 1051 | #trade |
| 119 | #biodiversity | 430 | #glowsticks | 741 | #narusemikio | 1052 | #tradeasiainasia |
| 120 | #bioinformedicine | 431 | #godiva | 742 | #natgeo | 1053 | #tradefinance |
| 121 | #biotech | 432 | #goldenglobeaward | 743 | #natgeotravel | 1054 | #trademission |
| 122 | #birman | 433 | #gong | 744 | #nationalday | 1055 | #tradetech |
| 123 | #birthday | 434 | #goodseedprogramme | 745 | #nationality | 1056 | #trading |
| 124 | #blackandwhite | 435 | #graduation | 746 | #natural | 1057 | #trainguard |
| 125 | #bladerunner | 436 | #grandillusion | 747 | #nature | 1058 | #trami |
| 126 | #blockchain | 437 | #greaterbayarea | 748 | #navigatingbusiness | 1059 | #transformtion |
| 127 | #boat | 438 | #greatfriendgetaway | 749 | #neon | 1060 | #translational |
| 128 | #bodw | 439 | #green | 750 | #newsemester | 1061 | #transport |
| 129 | #bond | 440 | #greenfinance | 751 | #newstart | 1062 | #transportation |
| 130 | #bondconnect | 441 | #greenpeace | 752 | #newzealand | 1063 | #trauma |
| 131 | #book | 442 | #growth | 753 | #nextacthk | 1064 | #travel |
| 132 | #bookclub | 443 | #guangdong | 754 | #ngo | 1065 | #travel2hongkong |
| 133 | #booklaunch | 444 | #gufa | 755 | #nico | 1066 | #traveladdict |
| 134 | #books | 445 | #guggenheim | 756 | #nipt | 1067 | #travelblog |
| 135 | #bootcamp | 446 | #hainanshaoye | 757 | #nismo | 1068 | #travelblogger |
| 136 | #brandshk | 447 | #halloween | 758 | #nomoreplasticplease | 1069 | #travelbloggers |
| 137 | #breakfreefromplastic | 448 | #halloweenfest | 759 | #nongfuspring | 1070 | #travelgram |
| 138 | #breaking | 449 | #hambacherforest | 760 | #noplasticstraws | 1071 | #travelphotography |
| 139 | #brigittebardot | 450 | #hambacherforst | 761 | #novel | 1072 | #travelpic |
| 140 | #broadcast | 451 | #hambibleibt | 762 | #nrt | 1073 | #traveltip |
| 141 | #brownfield | 452 | #handicraft | 763 | #ntr | 1074 | #traveltips |
| 142 | #brunei | 453 | #haneda | 764 | #ocarina | 1075 | #traveltomorrow |
| 143 | #business | 454 | #happymidautumn | 765 | #occupational | 1076 | #traveltuesday |
| 144 | #businessclass | 455 | #happymidautumnfestival | 766 | #oceanpark | 1077 | #treasurehunt |
| 145 | #businesspriorities | 456 | #harbour | 767 | #office | 1078 | #treatment |
| 146 | #buybeforeyoufly | 457 | #harbourcity | 768 | #oi | 1079 | #trentino |
| 147 | #buyma | 458 | #hardware | 769 | #oka | 1080 | #tricks |
| 148 | #buyma_ps | 459 | #harvard | 770 | #okinawa | 1081 | #trueloveortruelies |
| 149 | #cafedecoral | 460 | #hbasia | 771 | #oldboy | 1082 | #trulyhk |
| 150 | #california | 461 | #hcart | 772 | #oman | 1083 | #trulyhongkong |
| 151 | #cantonese | 462 | #hcbeauty | 773 | #online | 1084 | #tsinghua |
| 152 | #cantoneseopera | 463 | #hcfashion | 774 | #onlinebooths | 1085 | #tuesdaytrivia |
| 153 | #canvastotebag | 464 | #hcfood | 775 | #onlineexhibition | 1086 | #tuina |
| 154 | #capitalmarkets | 465 | #hcsunset | 776 | #onlineguidedtours | 1087 | #turbulence |
| 155 | #career | 466 | #health | 777 | #onlinelecture | 1088 | #twitterstorians |
| 156 | #carfreeday | 467 | #healthcare | 778 | #onlinescam | 1089 | #tx2 |
| 157 | #carrielam | 468 | #healthtech | 779 | #onlineshop | 1090 | #typhoon |
| 158 | #casestudies | 469 | #healthtechnology | 780 | #onlinestore | 1091 | #typhoonmangkhut |
| 159 | #casestudy | 470 | #heimtextil | 781 | #ontario | 1092 | #typhoons |
| 160 | #cashflow | 471 | #hellokitty | 782 | #onthisday | 1093 | #uae |
| 161 | #cattledepotartpark | 472 | #herbal | 783 | #opdancejamfever | 1094 | #unesco |
| 162 | #cbre | 473 | #heritage | 784 | #openbanking | 1095 | #unga |
| 163 | #cdta | 474 | #highlights | 785 | #opendialogue | 1096 | #university |
| 164 | #central | 475 | #hiking | 786 | #openmuseums | 1097 | #universityfellows |
| 165 | #centralhk | 476 | #history | 787 | #opera | 1098 | #upr31 |
| 166 | #centrestage | 477 | #hk | 788 | #orientation | 1099 | #urbanoasis |
| 167 | #centrestagehk | 478 | #hkairkines | 789 | #osaka | 1100 | #urbansketchers |
| 168 | #ceramic | 479 | #hkairlines | 790 | #oscar | 1101 | #urbansketchershk |
| 169 | #champ | 480 | #hkbfilms | 791 | #outstandingstudent | 1102 | #us |
| 170 | #championship | 481 | #hkbu | 792 | #overlab | 1103 | #usa |
| 171 | #changethewaywelive | 482 | #hkbuaao | 793 | #ozone | 1104 | #uschina |
| 172 | #charlenechoi | 483 | #hkbuaf | 794 | #pakhochau | 1105 | #usd |
| 173 | #chemicals | 484 | #hkbuniversity | 795 | #pandemic | 1106 | #usrussia |
| 174 | #cheongsam | 485 | #hkcec | 796 | #papillon | 1107 | #vaccinationsubsidyscheme |
| 175 | #china | 486 | #hkcentral | 797 | #parking | 1108 | #vaporwave |
| 176 | #chinafintech | 487 | #hkchurch | 798 | #partnerships | 1109 | #vc |
| 177 | #chinanationalday | 488 | #hkex | 799 | #patricknip | 1110 | #venchi |
| 178 | #chinatang | 489 | #hkexrmbfic | 800 | #paulandjoe | 1111 | #victoriaharbour |
| 179 | #chinaus | 490 | #hkfintechweek | 801 | #paulchan | 1112 | #violin |
| 180 | #chinese | 491 | #hkg | 802 | #paymeforbusiness | 1113 | #virtual |
| 181 | #chinesecinema | 492 | #hkga | 803 | #paymeforgood | 1114 | #virtualbank |
| 182 | #chinesemedicine | 493 | #hkgolf | 804 | #payments | 1115 | #virtualbanking |
| 183 | #chinesepainting | 494 | #hkia | 805 | #peptides | 1116 | #virtualexihibitionbooths |
| 184 | #chinesewhitedolphin | 495 | #hkiff | 806 | #persona | 1117 | #visiongo |
| 185 | #chloechan | 496 | #hkig | 807 | #personalgrowth | 1118 | #visitkazakhstan |
| 186 | #chrisfujiwara | 497 | #hkilf | 808 | #pgatourchina | 1119 | #volcanoes |
| 187 | #chronicstroke | 498 | #hkla | 809 | #philanthropy | 1120 | #volunteer |
| 188 | #church | 499 | #hkpf | 810 | #philippines | 1121 | #vote |
| 189 | #churchhk | 500 | #hkpolicyaddress | 811 | #photodojo | 1122 | #vr |
| 190 | #chuu | 501 | #hkracing | 812 | #photographers | 1123 | #vsco |
| 191 | #cie | 502 | #hksar | 813 | #photography | 1124 | #waiver |
| 192 | #cinefan | 503 | #hkstp | 814 | #photooftheday | 1125 | #walkfornature |
| 193 | #cineitaliano | 504 | #hkteam | 815 | #physics | 1126 | #wanchai |
| 194 | #cineitaliano2018 | 505 | #hku | 816 | #piano | 1127 | #wanderlust |
| 195 | #city | 506 | #hkuinyourcountry | 817 | #pickupatairport | 1128 | #washington |
| 196 | #cityoflife | 507 | #hkusbs | 818 | #pitching | 1129 | #wcdhongkong |
| 197 | #cityscape | 508 | #hkust | 819 | #planet | 1130 | #wealth |
| 198 | #civilservice | 509 | #hnd | 820 | #plastic | 1131 | #wealthbeing |
| 199 | #clamdigging | 510 | #holisticeducation | 821 | #plasticfree | 1132 | #wealthmanagement |
| 200 | #cleanenergy | 511 | #homeaffairsdepartment | 822 | #plasticfreenow | 1133 | #wealthtech |
| 201 | #climate | 512 | #hongkong | 823 | #plastics | 1134 | #wearepolyu |
| 202 | #climateaction | 513 | #hongkongbaptistuniversity | 824 | #police | 1135 | #weather |
| 203 | #climatechange | 514 | #hongkongcyclothon | 825 | #policyaddress | 1136 | #webcast |
| 204 | #climatejustice | 515 | #hongkonger | 826 | #pollution | 1137 | #webinar |
| 205 | #climateweeknyc | 516 | #hongkongfashionsummit | 827 | #polyu | 1138 | #webinars |
| 206 | #cloud | 517 | #honia | 828 | #polyudesigndegreeshow | 1139 | #weddingsbymo |
| 207 | #cloudy | 518 | #honorary | 829 | #polyuresearch | 1140 | #weekendvibes |
| 208 | #clubautus | 519 | #horizon | 830 | #polyuresearcher | 1141 | #welcome |
| 209 | #cnh | 520 | #hospitality | 831 | #polyuresearchers | 1142 | #welcometosustainablelife |
| 210 | #coal | 521 | #hotel | 832 | #polyustudent | 1143 | #welcoming |
| 211 | #coffee | 522 | #hotels | 833 | #portfolio | 1144 | #wellbeing |
| 212 | #coffeeacademics | 523 | #hotlines | 834 | #pos | 1145 | #wellness |
| 213 | #coffeemug | 524 | #hotoffthepress | 835 | #postgraduate | 1146 | #wepickhk |
| 214 | #collaboration | 525 | #hsbc | 836 | #power | 1147 | #wherehkbegins |
| 215 | #collectibles | 526 | #hsbcbusinesstalks | 837 | #prada | 1148 | #whiskersandfriends |
| 216 | #colombia | 527 | #hsbccookingfriday | 838 | #prenatal | 1149 | #whisky |
| 217 | #colourbrown | 528 | #hsbcembracehk | 839 | #press | 1150 | #windy |
| 218 | #commencement | 529 | #hsbcglobalresearch | 840 | #priority | 1151 | #wine |
| 219 | #commodities | 530 | #hsbchkcommunity | 841 | #professor | 1152 | #winterlight |
| 220 | #communication | 531 | #hsbclife | 842 | #programmeinfoseminars | 1153 | #womeninfinance |
| 221 | #communications | 532 | #hsbcnavigator | 843 | #property | 1154 | #workday |
| 222 | #compliance | 533 | #hsbcone | 844 | #protectantarctic | 1155 | #workintegratededucation |
| 223 | #computer | 534 | #hsbcprivatebanking | 845 | #psychology | 1156 | #workwear |
| 224 | #concert | 535 | #hsbcresearch | 846 | #publichealth | 1157 | #world |
| 225 | #conference | 536 | #hsbcvolunteer | 847 | #pulitzer | 1158 | #worldanimalday |
| 226 | #conflict | 537 | #htls | 848 | #qinlan | 1159 | #worldinvestorweek |
| 227 | #congratulations | 538 | #htls2018 | 849 | #quanalley | 1160 | #worldmentalhealthday |
| 228 | #connect2earth | 539 | #huf | 850 | #raceagainstextinction | 1161 | #worldmigratorybirdday |
| 229 | #connecter | 540 | #humanities | 851 | #radio | 1162 | #worldteachersday |
| 230 | #conservation | 541 | #humanity | 852 | #rafflesfamilyoffice | 1163 | #writer |
| 231 | #construction | 542 | #humannervoussystem | 853 | #ranking | 1164 | #writing |
| 232 | #containers | 543 | #humans | 854 | #readabookday | 1165 | #wrldcty |
| 233 | #contemporaryart | 544 | #hurricaneflorence | 855 | #readwithhkilf | 1166 | #wu |
| 234 | #contributions | 545 | #ibbdigital | 856 | #realestate | 1167 | #wuchun |
| 235 | #convocation | 546 | #ibond | 857 | #realitygames | 1168 | #wuculture |
| 236 | #coolhandluke | 547 | #ican2020 | 858 | #reasonsforhope | 1169 | #yaumatei |
| 237 | #coral | 548 | #icoti18 | 859 | #reclamation | 1170 | #ydc |
| 238 | #cornet | 549 | #ifc | 860 | #recreational | 1171 | #yearofthedog |
| 239 | #corporategovernance | 550 | #ifec | 861 | #redchamberaward | 1172 | #yourshot |
| 240 | #costagavras | 551 | #ifta | 862 | #redressdesignaward | 1173 | #youth |
| 241 | #countdown | 552 | #illusion | 863 | #refusetobenumb | 1174 | #zenith |
| 242 | #countryparks | 553 | #ilovehongkong | 864 | #regatta | 1175 | #バイマ |
| 243 | #craftbeer | 554 | #imafan | 865 | #regtech | 1176 | #百勝中國 |
| 244 | #cre | 555 | #immersive3dshow | 866 | #regulation | 1177 | #北角 |
| 245 | #creative | 556 | #immigration | 867 | #rehabilitationprogramme | 1178 | #必勝客 |
| 246 | #criesandwhispers | 557 | #inclusion | 868 | #rehabilitationsciences | 1179 | #超級颱風山竹 |
| 247 | #crisis | 558 | #inclusive | 869 | #reindustrialization | 1180 | #陳俊樂 |
| 248 | #cruise | 559 | #incubator | 870 | #related_to | 1181 | #成田 |
| 249 | #cultural | 560 | #india | 871 | #renewable | 1182 | #沖繩 |
| 250 | #culture | 561 | #indiabythebay | 872 | #renewableenergy | 1183 | #創新療法 |
| 251 | #cupp2018 | 562 | #indonesia | 873 | #rescuedrill | 1184 | #唇典 |
| 252 | #currency | 563 | #influencer | 874 | #research | 1185 | #大阪 |
| 253 | #customs | 564 | #ingmarbergman | 875 | #researchers | 1186 | #電器 |
| 254 | #cvcf | 565 | #innovation | 876 | #resilience | 1187 | #東京成田 |
| 255 | #cvcf2020 | 566 | #innovations | 877 | #resilient | 1188 | #東京羽田 |
| 256 | #cxflypink | 567 | #inr | 878 | #retail | 1189 | #個人護理 |
| 257 | #cyberport | 568 | #insidertravel | 879 | #retailbanking | 1190 | #恭喜 |
| 258 | #cyberportcommunity | 569 | #insta_sky_lovers | 880 | #revitalisation | 1191 | #観覧車 |
| 259 | #cyberpunk | 570 | #instadaily | 881 | #ringthebell | 1192 | #觀塘 |
| 260 | #cycling | 571 | #instagram | 882 | #rise2018 | 1193 | #好丘 |
| 261 | #dalloyau | 572 | #instapic | 883 | #rmb | 1194 | #恒生科技指數 |
| 262 | #data | 573 | #instasky | 884 | #roaminghongkong | 1195 | #紅樓夢獎 |
| 263 | #dating | 574 | #instatravel | 885 | #robotics | 1196 | #歡迎 |
| 264 | #davidbeckham | 575 | #institutionalinvestors | 886 | #robots | 1197 | #環保 |
| 265 | #deathinvenice | 576 | #insurance | 887 | #romance | 1198 | #環保觸覺 |
| 266 | #degree | 577 | #insurtech | 888 | #roomwithaview | 1199 | #機場快綫 |
| 267 | #delayed | 578 | #insurtechworldtour | 889 | #rowing | 1200 | #交易所債券市場 |
| 268 | #depression | 579 | #interdisciplinary | 890 | #rubylectures | 1201 | #酒店 |
| 269 | #derivatives | 580 | #interiordesign | 891 | #ruleoflaw | 1202 | #開市前時段 |
| 270 | #design | 581 | #international | 892 | #run | 1203 | #肯德基 |
| 271 | #designdegreeshow | 582 | #internationalchurch | 893 | #russia | 1204 | #寬巷子 |
| 272 | #designinspire | 583 | #internationalisation | 894 | #safety | 1205 | #劉慶 |
| 273 | #designthinking | 584 | #internet | 895 | #samsunggalaxys8 | 1206 | #六個月交易費用 |
| 274 | #development | 585 | #internetgovernance | 896 | #sanfrancisco | 1207 | #名古屋 |
| 275 | #dfintech | 586 | #invention | 897 | #sanriocharacters | 1208 | #農夫山泉 |
| 276 | #dialogue | 587 | #investment | 898 | #sanriohk | 1209 | #敲鑼 |
| 277 | #digital | 588 | #iot | 899 | #saraband | 1210 | #人民幣定息及貨幣論壇 |
| 278 | #digitalpayment | 589 | #ipcc | 900 | #sarahlee | 1211 | #沙田至中環綫 |
| 279 | #digitaltransformation | 590 | #ipo | 901 | #saveourcountryparks | 1212 | #上市 |
| 280 | #digitisation | 591 | #isda | 902 | #savetheocean | 1213 | #上市直播 |
| 281 | #dimsum | 592 | #isdabenchmarks | 903 | #saynotoivory | 1214 | #上午交易時段 |
| 282 | #discoverhongkong | 593 | #isdavc | 904 | #saynotosharkfin | 1215 | #生物科技公司 |
| 283 | #discrimination | 594 | #islandhousefestival | 905 | #school | 1216 | #生物製藥公司 |
| 284 | #disinfection | 595 | #italy | 906 | #science | 1217 | #石硤尾 |
| 285 | #disneyland | 596 | #itshelloween | 907 | #scientists | 1218 | #數字化 |
| 286 | #disruption | 597 | #iww | 908 | #sclphase2 | 1219 | #塔可貝爾 |
| 287 | #distinguished | 598 | #jaimehayon | 909 | #scotland | 1220 | #台灣 |
| 288 | #diverse | 599 | #jaimesitalian | 910 | #sculpture | 1221 | #潭美 |
| 289 | #diversity | 600 | #jakarta | 911 | #sdi20 | 1222 | #唐人館 |
| 290 | #diversityandinclusion | 601 | #jaotsungi | 912 | #seasonalinfluenza | 1223 | #威威與好友 |
| 291 | #diy | 602 | #japan | 913 | #seasonopener | 1224 | #維修香港 |
| 292 | #doctoral | 603 | #jar | 914 | #secondlanguagelearning | 1225 | #無冷氣夜 |
| 293 | #doglover | 604 | #jars | 915 | #seminar | 1226 | #香港 |
| 294 | #dogman | 605 | #jasic | 916 | #seoul | 1227 | #香港大學 |
| 295 | #doingwellbydoinggood | 606 | #jccac | 917 | #september | 1228 | #香港金融市場 |
| 296 | #dolomites | 607 | #jccac10 | 918 | #service | 1229 | #香港中環 |
| 297 | #donation | 608 | #johnerni | 919 | #shameonba | 1230 | #新加坡 |
| 298 | #dondondonki | 609 | #johnlee | 920 | #shamshuipo | 1231 | #新興市場 |
| 299 | #dress | 610 | #joinoursmartcommunity | 921 | #shark | 1232 | #須予公佈的交易 |
| 300 | #drone | 611 | #journalism | 922 | #sharkfin | 1233 | #亞洲 |
| 301 | #dualtaskexercise | 612 | #jpy | 923 | #shibuya109 | 1234 | #衍生產品市場 |
| 302 | #dubai | 613 | #jupas | 924 | #shikoku | 1235 | #銀紫荊星章 |
| 303 | #dustinhoffman | 614 | #justtransition | 925 | #shinzoabe | 1236 | #羽田 |
| 304 | #dyinginplace | 615 | #justwalk | 926 | #shioriito | 1237 | #證券 |
| 305 | #dyk | 616 | #katespade | 927 | #ships | 1238 | #證券市場 |
| 306 | #easonchan | 617 | #kato | 928 | #shopping | 1239 | #直播 |
| 307 | #eastraillinenewtrains | 618 | #kazakh | 929 | #shoppingonline | 1240 | #中環 |
| 308 | #ebusiness | 619 | #kazakhstan | 930 | #sibos | 1241 | #主板 |
| 309 | #ecommerce | 620 | #kiehlshk | 931 | #silenceofgodtrilogy | 1242 | #注意個人衛生多啲洗手 |
| 310 | #economicfreedom | 621 | #kiehlshkmadebetter | 932 | #singapore | 1243 | #做好防疫大家努力 |
| 311 | #economy | 622 | #kikkik | 933 | #singing | 1244 | #희망의이유 |

**Weibo mention contacts identified from tweets posted by Shenzhen-based organizational actors**

| No | Mention contact | No | Mention contact | No | Mention contact | No | Mention contact |
| --- | --- | --- | --- | --- | --- | --- | --- |
| 1 | @__宋凯__ | 245 | @广东省教育厅 | 489 | @明天你好洲哥 | 733 | @天津交通广播 |
| 2 | @_是珍珠啊 | 246 | @广东天气 | 490 | @铭铭铭铭记 | 734 | @-天凉好个秋呀- |
| 3 | @0S曉 | 247 | @广东消防 | 491 | @沫MOMO沫 | 735 | @天使anson |
| 4 | @12321举报中心 | 248 | @广东志愿者 | 492 | @莫名_D719 | 736 | @天天美食推荐 |
| 5 | @26Lx4 | 249 | @广铁集团广九客运段 | 493 | @默克生命科学 | 737 | @调峰调频发电公司 |
| 6 | @5大自然 | 250 | @广铁集团深圳北火车站 | 494 | @默默的爱上了她却不敢拥有了她 | 738 | @跳跳可爱虎 |
| 7 | @8900万党员 | 251 | @广铁集团长沙南火车站 | 495 | @默燃符 | 739 | @通心岭派出所 |
| 8 | @a_lex_liu | 252 | @广铁警方在线 | 496 | @沐川公安 | 740 | @铜仁供电 |
| 9 | @akeunggg | 253 | @广铁长沙客运段 | 497 | @慕容湘若 | 741 | @童童的外婆XD |
| 10 | @A-Li033 | 254 | @广西电视台 | 498 | @耐心的黄呵呵 | 742 | @头条新闻 |
| 11 | @BabyBaby蛋蛋 | 255 | @广西电视台新闻在线 | 499 | @男左女右201610 | 743 | @图们交警 |
| 12 | @Beyond的地盘 | 256 | @广西电台私家车930 | 500 | @南方+ | 744 | @土豆阿夏 |
| 13 | @BOSB-Z-H | 257 | @广西电台新闻910 | 501 | @南方电网报 | 745 | @瓦嘜 |
| 14 | @BOWEN妈 | 258 | @广西电网 | 502 | @南方都市报 | 746 | @万科公益基金会 |
| 15 | @Carsten邹念深 | 259 | @广西电网来宾供电 | 503 | @南方日报 | 747 | @万岁爷K-K |
| 16 | @CATL宁德时代新能源 | 260 | @广西共青团 | 504 | @南国早报 | 748 | @汪胖子的小心思 |
| 17 | @cctv7 | 261 | @广西来宾供电 | 505 | @南海公安 | 749 | @王石 |
| 18 | @CCTV今日说法 | 262 | @广西日报 | 506 | @南京冠生园官方微博 | 750 | @网络解码 |
| 19 | @CCTV生活圈 | 263 | @广西日报桂林记者站 | 507 | @南宁安监 | 751 | @威风十二面 |
| 20 | @CERAMICMARK | 264 | @广州地铁 | 508 | @南宁供电 | 752 | @威远公安 |
| 21 | @ce超市 | 265 | @广州公安 | 509 | @南宁日报 | 753 | @微博抽奖平台 |
| 22 | @che1yz | 266 | @广州供电 | 510 | @南宁铁路 | 754 | @微博电影工厂 |
| 23 | @CHENGZIHONG_81124 | 267 | @广州海珠发布 | 511 | @南山交警大队 | 755 | @微博管理员 |
| 24 | @Chenxuzhi | 268 | @广州交警 | 512 | @南山石头人 | 756 | @微博科普 |
| 25 | @chowkoonwah | 269 | @广州交通 | 513 | @南网50Hz | 757 | @微博媒体直播 |
| 26 | @ChrisZhuang10 | 270 | @广州交通电台 | 514 | @南网的电粉 | 758 | @微公益 |
| 27 | @Cutie_Xu | 271 | @广州酒家集团 | 515 | @南园派出所 | 759 | @微信开门 |
| 28 | @devilchen_ | 272 | @广州民防发布 | 516 | @内含子 | 760 | @微言教育 |
| 29 | @easy怡 | 273 | @广州南车站 | 517 | @你 | 761 | @巍甜的澜瓜子 |
| 30 | @Flanker711 | 274 | @广州日报 | 518 | @你不准骂我因为我名字长 | 762 | @為何你的笑 |
| 31 | @FM1052羊城交通台 | 275 | @广州天气 | 519 | @你的温柔只许我拥有93889 | 763 | @维哪个荷哩 |
| 32 | @FuckingRabbit | 276 | @广州铁路 | 520 | @你忙吧我吃柠檬- | 764 | @潍坊公安 |
| 33 | @Grey瑞 | 277 | @贵港供电 | 521 | @你笑着就老了 | 765 | @卫龙食品 |
| 34 | @gromit | 278 | @贵阳供电 | 522 | @你只属于我20121314 | 766 | @未来网 |
| 35 | @guoxf008 | 279 | @贵州电网 | 523 | @匿名用户_91576 | 767 | @未命名的ID |
| 36 | @H_寶bao2小葱头_ | 280 | @桂林百事帮 | 524 | @宁POLICE汪 | 768 | @喂·你在吗 |
| 37 | @hlq0286 | 281 | @桂林供电 | 525 | @宁国特巡警在线 | 769 | @蔚来 |
| 38 | @HoT_BeLLa | 282 | @桂林气象 | 526 | @女王范的隔夜茶 | 770 | @温妮umms |
| 39 | @HuAnG427- | 283 | @桂林最新资讯 | 527 | @爬上河边的螃蟹横着散步 | 771 | @-温锐杰- |
| 40 | @Hwoi | 284 | @国家电网报 | 528 | @潘九堂 | 772 | @温州交警 |
| 41 | @iPanda熊猫频道 | 285 | @国家应急广播 | 529 | @澎湃新闻 | 773 | @文明广东 |
| 42 | @Ivy_bb默默 | 286 | @国网福建电力 | 530 | @平安宝安 | 774 | @文山供电局 |
| 43 | @JayneLam_ | 287 | @国网四川电力 | 531 | @平安滨江 | 775 | @我独自走在郊外的小路上_小红帽 |
| 44 | @JUMP在望THETOP | 288 | @国资小新 | 532 | @平安广元 | 776 | @我行MAXUS |
| 45 | @kalikashang | 289 | @过山左转 | 533 | @平安桂林 | 777 | @我们说电线掉下来了 |
| 46 | @kehen2000 | 290 | @哈尔滨铁路局 | 534 | @平安惠阳 | 778 | @我是迷__ |
| 47 | @Lanple大王 | 291 | @哈工大 | 535 | @平安茂名 | 779 | @我是小猪Babe |
| 48 | @Lanple大王儿 | 292 | @海口供电 | 536 | @平安梅州 | 780 | @我是一只兔沫沫 |
| 49 | @LC-suli @知世子- | 293 | @海口同城 | 537 | @平安纳溪 | 781 | @我只是贪官的传说 |
| 50 | @LeeSoo光 | 294 | @海南电网 | 538 | @平安南京 | 782 | @无言感激-谭咏麟 |
| 51 | @Lin健 | 295 | @海南航空 | 539 | @平安南粤 | 783 | @吴江经济技术开发区派出所 |
| 52 | @Lisa1874 | 296 | @海南微博 | 540 | @平安清远 | 784 | @梧桐生矣木子朝阳 |
| 53 | @LIUYIMEI928 | 297 | @海豚佩佩 | 541 | @平安汕尾 | 785 | @梧州发布 |
| 54 | @li-yuanqiang | 298 | @海洋之心LMH | 542 | @平安双流 | 786 | @梧州供电 |
| 55 | @LQA1 | 299 | @航旅圈 | 543 | @平安随州 | 787 | @梧州零距离网 |
| 56 | @Marco_258 | 300 | @好多餘小姐 | 544 | @平安新都 | 788 | @梧州日报 |
| 57 | @miss_菜菜菜菜 | 301 | @合肥庐阳交警 | 545 | @平安徐州 | 789 | @梧州时空网 |
| 58 | @MK5202 | 302 | @和县城北派出所在线 | 546 | @平安渝中 | 790 | @梧州新闻 |
| 59 | @mlk0ne | 303 | @河池交警 | 547 | @平湖市公安局交警大队 | 791 | @武志红 |
| 60 | @MMonster_r | 304 | @河源供电 | 548 | @坪地卫生监督分所 | 792 | @西安饭庄 |
| 61 | @M大王叫我来巡山 | 305 | @河源交警 | 549 | @葡萄藤下一只鹅 | 793 | @西安交警新城大队 |
| 62 | @naihemo | 306 | @贺州供电 | 550 | @普洱供电官博 | 794 | @西子湖畔 |
| 63 | @Namtiveyo | 307 | @贺州交警 | 551 | @气象北京 | 795 | @熹躍 |
| 64 | @n空城n | 308 | @贺州微新闻 | 552 | @气象知识 | 796 | @现在名字是真难取 |
| 65 | @N视频 | 309 | @贺州新闻网 | 553 | @浅水鱼7Q | 797 | @乡村教师代言人-马云 |
| 66 | @PickupChina | 310 | @赫山公安分局 | 554 | @乔富敏 | 798 | @香菜要吃一大碗 |
| 67 | @ReANKHagram | 311 | @衡阳车务段 | 555 | @钦州供电 | 799 | @香港商報網 |
| 68 | @R-sir | 312 | @衡阳发布 | 556 | @秦淮警方 | 800 | @香蜜湖派出所 |
| 69 | @saruno | 313 | @衡阳铁路公安处 | 557 | @青春木木胡 | 801 | @享受生活的享 |
| 70 | @skzq336699 | 314 | @衡阳蒸湘公安 | 558 | @青岛同三高速交警 | 802 | @肖朝兵 |
| 71 | @Ssylvialiu | 315 | @红尘如梦Mr | 559 | @青平高速交警 | 803 | @消除联萌天团 |
| 72 | @Sun容R | 316 | @红树林基金会 | 560 | @轻松公益 | 804 | @潇湘晨报 |
| 73 | @SY东北虎 | 317 | @红网 | 561 | @清远天气 | 805 | @小醋0722 |
| 74 | @S车视觉 | 318 | @胡椒视频 | 562 | @情义丰县 | 806 | @小甘 |
| 75 | @TED君学演讲 | 319 | @湖北高警下陆大队 | 563 | @屈楚萧 | 807 | @小柑橘超可爱 |
| 76 | @tenwanwhy | 320 | @湖北高速交警巴东大队 | 564 | @曲靖供电局 | 808 | @小高同學-Y |
| 77 | @thriller08435 | 321 | @湖北高速交警公安大队 | 565 | @全球视频大魔王 | 809 | @小黑兔不乖888 |
| 78 | @Tiger公子 | 322 | @湖北高速交警汉川大队 | 566 | @人民公安报 | 810 | @小惠惠灰hui_ |
| 79 | @tonyweeyee | 323 | @湖北高速交警黄梅大队 | 567 | @人民日报 | 811 | @小姐姐您憋闹 |
| 80 | @TURBOCHINA刘昊然中文站 | 324 | @湖北高速交警利川大队 | 568 | @人民铁道 | 812 | @小橘纸tangerine |
| 81 | @Uncle_虎三 | 325 | @湖北高速交警麻城大队 | 569 | @人民网 | 813 | @小了个楠 |
| 82 | @vikin-liu | 326 | @湖北高速交警随州大队 | 570 | @人人公益官微 | 814 | @小四哆哆 |
| 83 | @vwvw1234 | 327 | @湖北高速交警下陆大队 | 571 | @人人视频 | 815 | @小小小小熊熊熊熊熊熊 |
| 84 | @WHG温德姆 | 328 | @湖北高速交警兴山大队 | 572 | @肉肉肉肉肉肉肉肉... | 816 | @小小芋头君 |
| 85 | @Wilber汤 | 329 | @湖北高速交警郧西大队 | 573 | @肉肉肉肉肉肉肉肉肉肉肉肉S | 817 | @小小章鱼儿555 |
| 86 | @WorldCleanupDay | 330 | @湖北高速交警长阳大队 | 574 | @如东肖蜀黍 | 818 | @小杨杨水斌啦 |
| 87 | @WWF世界自然基金会 | 331 | @湖北高速交警钟祥大队 | 575 | @如果-瀞沚 | 819 | @新华视点 |
| 88 | @xiahua浅笑 | 332 | @湖北高速警察罗田大队 | 576 | @撒哈拉的一抹绿020 | 820 | @新华网 |
| 89 | @XXX259333XXX | 333 | @湖北随州交警 | 577 | @三沙供电 | 821 | @新京报 |
| 90 | @X恋恋洋葱的小媳妇22 | 334 | @湖南高速警察 | 578 | @三亚供电 | 822 | @新京报我们视频 |
| 91 | @Y_CHAN_Y仨點兒零版本 | 335 | @湖南公安 | 579 | @桑菜sunny | 823 | @新快报 |
| 92 | @yingtaobuding7 | 336 | @湖南交通频道官方微博 | 580 | @森野矢裕 | 824 | @新浪广东 |
| 93 | @yongmoor | 337 | @湖南省交警总队 | 581 | @沙头派出所 | 825 | @新浪广东惠州 |
| 94 | @yxl找不着北 | 338 | @花自有开期 | 582 | @厦门六中合唱团 | 826 | @新浪广东湛江 |
| 95 | @阿里巴巴公益 | 339 | @华齐 | 583 | @陕西消防 | 827 | @新浪广东珠海 |
| 96 | @阿里巴巴公益基金会 | 340 | @画满颜色的萝卜 | 584 | @汕头供电 | 828 | @新浪广西 |
| 97 | @唉呀妈呀脑瓜疼脑瓜疼 | 341 | @话痨er | 585 | @汕头市政府应急办 | 829 | @新浪海南 |
| 98 | @爱德基金会 | 342 | @淮北公安交警在线 | 586 | @汕尾发布 | 830 | @新浪湖南 |
| 99 | @爱民警长 | 343 | @淮北交警二大队 | 587 | @汕尾供电 | 831 | @新浪深圳 |
| 100 | @爱心特区 | 344 | @淮南公安交警在线 | 588 | @商铺老板们 | 832 | @新力地产 |
| 101 | @安德莉亚-- | 345 | @淮南公安在线 | 589 | @韶关发布 | 833 | @新时代新罗湖 |
| 102 | @安庆公安交警在线 | 346 | @淮南治安在线 | 590 | @韶关供电 | 834 | @新闻不发炎 |
| 103 | @安庆公安在线 | 347 | @环球网 | 591 | @蛇口消息报 | 835 | @星光公益联盟 |
| 104 | @安庆宿松公安在线 | 348 | @皇岗口岸派出所 | 592 | @蛇口邮轮中心king | 836 | @腥风血雨大裤衩 |
| 105 | @安州交警 | 349 | @黄岛交警 | 593 | @深房经纪人 | 837 | @兴义供电 |
| 106 | @黯黯猪 | 350 | @黄山公安在线 | 594 | @深圳宝安交警 | 838 | @修炼不到千年的夜猫子 |
| 107 | @八卦岭派出所 | 351 | @黄圣依 | 595 | @深圳宝安网 | 839 | @徐子烁HH |
| 108 | @白风男子 | 352 | @灰小哀onie | 596 | @深圳北站交通枢纽 | 840 | @宣城公安交警在线 |
| 109 | @白云机场 | 353 | @惠州电台988 | 597 | @深圳大剧院 | 841 | @宣城公安在线 |
| 110 | @百度公益 | 354 | @惠州公安 | 598 | @深圳大鹏半岛国家地质公园 | 842 | @寻萤者付新华 |
| 111 | @绑定原光明新区人民医院 | 355 | @惠州供电 | 599 | @深圳大鹏交警 | 843 | @闫阳 |
| 112 | @蚌埠公安龙子湖分局 | 356 | @惠州交警 | 600 | @深圳地铁集团 | 844 | @盐田发布 |
| 113 | @宝安发布 | 357 | @惠州天气 | 601 | @深圳地铁义工联 | 845 | @盐田公安 |
| 114 | @宝安日报 | 358 | @吉林发布 | 602 | @深圳地铁运营 | 846 | @盐田交警 |
| 115 | @宝安图书馆 | 359 | @吉林省交警总队 | 603 | @深圳电台先锋898 | 847 | @央企头条 |
| 116 | @宝洁中国 | 360 | @吉林微政务 | 604 | @深圳东部高速交警 | 848 | @央视财经 |
| 117 | @保尔森基金会 | 361 | @极限轮滑-ZK | 605 | @深圳镀膜材料批发 | 849 | @央视网 |
| 118 | @北海供电 | 362 | @疾奔的snail | 606 | @深圳反诈 | 850 | @央视新闻 |
| 119 | @北海交警 | 363 | @记得吃_早饭 | 607 | @深圳福田交警 | 851 | @羊城晚报 |
| 120 | @北京口腔医院 | 364 | @济南公安公共交通分局 | 608 | @深圳福田交警根据大数据分析研判 | 852 | @羊城晚报金羊网 |
| 121 | @本宫今天不回宫 | 365 | @绩溪县金沙派出所 | 609 | @深圳各生产经营单位 | 853 | @阳光宝安 |
| 122 | @笾笾笾笾笾笾c | 366 | @剪刀手黄小贱 | 610 | @深圳公安 | 854 | @阳江发布 |
| 123 | @博物小馆 | 367 | @简简单单俊 | 611 | @深圳公交公安 | 855 | @阳江供电 |
| 124 | @不爱拍车的飞机 | 368 | @建筑深圳 | 612 | @深圳共青团 | 856 | @杨sir在线 |
| 125 | @不爱也不会伤害你 | 369 | @健康宝安 | 613 | @深圳供电 | 857 | @杨幂 |
| 126 | @不买到320不改名字 | 370 | @健康-广东 | 614 | @深圳关爱行动 | 858 | @杨浦交警 |
| 127 | @不知名网友小王丶 | 371 | @健康深圳 | 615 | @深圳光明交警 | 859 | @楊祐寧YOYANG |
| 128 | @布吉街道 | 372 | @健康时报 | 616 | @深圳国资 | 860 | @叶荣添 |
| 129 | @擦肩而过的缘分 | 373 | @健康中国 | 617 | @深圳航空 | 861 | @叶踏汐 |
| 130 | @草根宅男Maple | 374 | @江江_江江江 | 618 | @深圳机场 | 862 | @叶叶叶聚 |
| 131 | @策马西风扬鞭 | 375 | @江门电台 | 619 | @深圳机场交警 | 863 | @夜夏半凉v |
| 132 | @常熟-辛庄派出所 | 376 | @江门供电 | 620 | @深圳家长 | 864 | @一风成形 |
| 133 | @唱歌的孤鲸 | 377 | @江门日报 | 621 | @深圳交警 | 865 | @一个来自未来的人 |
| 134 | @超电小魔女 ！ | 378 | @江宁公安在线 | 622 | @深圳交警车管所 | 866 | @一个全职司机 |
| 135 | @潮州供电 | 379 | @江苏气象 | 623 | @深圳交警根据大数据情报 | 867 | @一个热爱广州的80后 |
| 136 | @陈BigDiao | 380 | @江苏新闻 | 624 | @深圳交警机动训练大队 | 868 | @一和一传媒 |
| 137 | @陈花扬 | 381 | @江西高速交警一支队第五大队 | 625 | @深圳交警交通科技 | 869 | @一缕云朵 |
| 138 | @陈士渠 | 382 | @江油公安 | 626 | @深圳交警路况播报 | 870 | @一手Video |
| 139 | @成都地铁 | 383 | @江逾淮 | 627 | @深圳交警在金安路查获一酒驾司机江某 | 871 | @一位医院看门大爷的思考 |
| 140 | @成都地铁运营 | 384 | @姜丶成林 | 628 | @深圳教育 | 872 | @伊人忘归 |
| 141 | @成都商报1视频 | 385 | @狡猾的狐狸- | 629 | @深圳李玉芝 | 873 | @医学界网站 |
| 142 | @诚然不想 | 386 | @叫我朱叉叉 | 630 | @深圳凉薄青年 | 874 | @壹基金 |
| 143 | @吃菜虫的小扁 | 387 | @揭阳供电 | 631 | @深圳龙岗交警 | 875 | @壹基金官方公益店 |
| 144 | @池州公安交警直属二大队在线 | 388 | @揭阳同城网 | 632 | @深圳龙岗教育微博 | 876 | @壹基金联合救灾 |
| 145 | @崇左供电 | 389 | @揭阳新闻发布厅 | 633 | @深圳龙岗警营 | 877 | @宜宾县公安 |
| 146 | @宠崇7 | 390 | @今日头条 | 634 | @深圳龙华发布 | 878 | @宜州交警 |
| 147 | @楚天都市报 | 391 | @金色莲花2017 | 635 | @深圳龙华公安 | 879 | @义仓发展网络 |
| 148 | @楚雄供电局 | 392 | @金山警务百事通 | 636 | @深圳龙华交警 | 880 | @银dai- |
| 149 | @创新南山 | 393 | @金湾发布 | 637 | @深圳龙华教育 | 881 | @颖子爱三元 |
| 150 | @聪聪的美发用品店 | 394 | @金羊网 | 638 | @深圳论坛 | 882 | @喲癯 |
| 151 | @崔家的菇凉 | 395 | @锦州公安 | 639 | @深圳罗湖交警 | 883 | @永嘉交警 |
| 152 | @大海周游世界 | 396 | @晶报 | 640 | @深圳每日头条 | 884 | @詠c_c |
| 153 | @大理祥云 | 397 | @晶报公益周刊 | 641 | @深圳妙雨 | 885 | @用户地铁义工三组 |
| 154 | @大连户口身份证 | 398 | @晶报体育 | 642 | @深圳南山交警 | 886 | @用户海洋之杨洋兄弟 |
| 155 | @大连交警 | 399 | @景田派出所 | 643 | @深圳南山教育 | 887 | @有点傻乎乎的丫头 |
| 156 | @大貓TheGreatCat | 400 | @警花LiLy | 644 | @深圳南山网 | 888 | @鱼児有翅不会Fei |
| 157 | @大漠驿站188 | 401 | @警民携手同行 | 645 | @深圳胖纸蝈蝈 | 889 | @俞灏明 |
| 158 | @大鹏发布 | 402 | @警民直通车-浦东 | 646 | @深圳坪山交警 | 890 | @雨城交警 |
| 159 | @大鹏公安 | 403 | @警民直通车-上海 | 647 | @深圳侨报 | 891 | @玉林供电 |
| 160 | @大小姐丁 | 404 | @九牧Jomoo | 648 | @深圳轻描淡写画廊 | 892 | @玉林同城 |
| 161 | @大洋网 | 405 | @菊花馨 | 649 | @深圳热门情报站 | 893 | @玉林晚报 |
| 162 | @大自然保护协会TNC | 406 | @决不掉队 | 650 | @深圳人 | 894 | @远山红叶989 |
| 163 | @岱青山人 | 407 | @军报记者 | 651 | @深圳人力资源和社会保障局 | 895 | @岳屾山 |
| 164 | @带上梦想启航 | 408 | @俊興Gnome | 652 | @深圳山地救援队 | 896 | @云浮供电 |
| 165 | @戴冠宏教练 | 409 | @开心奕博 | 653 | @深圳商报 | 897 | @云浮同城 |
| 166 | @但斌 | 410 | @凯里供电 | 654 | @深圳蛇口港交警 | 898 | @云南电网公司 |
| 167 | @德宏供电局 | 411 | @看法新闻 | 655 | @深圳食品药品监管 | 899 | @云南电网红河供电局 |
| 168 | @德州高速交警 | 412 | @看看新闻KNEWS | 656 | @深圳市场和质量监管 | 900 | @再远也只是场梦 |
| 169 | @德州运河公安分局 | 413 | @看看新闻Knews | 657 | @深圳市城市管理局 | 901 | @湛江交警 |
| 170 | @地球人有什么可怕 | 414 | @科技view | 658 | @深圳市公园管理中心 | 902 | @湛江新闻网 |
| 171 | @地铁义工八组 | 415 | @科技日报 | 659 | @深圳市关爱行动公益基金会 | 903 | @张家港市公安局行政拘留 |
| 172 | @地铁义工二组 | 416 | @科普中国 | 660 | @深圳市规划和国土资源委 | 904 | @张胖吃不胖 |
| 173 | @地铁义工六组 | 417 | @可口可乐中国 | 661 | @深圳市交警大队_665 | 905 | @张天育啊 |
| 174 | @地铁义工七组 | 418 | @客户世界网 | 662 | @深圳市交通运输委员会 | 906 | @漳州消防 |
| 175 | @地铁义工四组 | 419 | @空中的士马宏 | 663 | @深圳市垃圾减量分类 | 907 | @长安交通 |
| 176 | @地铁义工五组 | 420 | @快乐1062 | 664 | @深圳市民政局 | 908 | @长塘阜 |
| 177 | @地铁义工一组 | 421 | @快乐的牛牛0755-023 | 665 | @深圳市青少年发展基金会 | 909 | @掌上肇庆 |
| 178 | @第一现场 | 422 | @来宾公安 | 666 | @深圳市人民检察院 | 910 | @昭通供电 |
| 179 | @丶枫丶枫丶 | 423 | @莱城公安 | 667 | @深圳市社会保险基金管理局 | 911 | @赵先生微言薄语 |
| 180 | @电台罗小刚 | 424 | @涞源交警 | 668 | @深圳市食品行业协会 | 912 | @肇庆发布 |
| 181 | @电网头条 | 425 | @赖艺Leon | 669 | @深圳市水务局 | 913 | @肇庆高要供电局团委 |
| 182 | @屌炸天的小学弟 | 426 | @兰州公安 | 670 | @深圳市消费者委员会 | 914 | @肇庆供电 |
| 183 | @丁香医生 | 427 | @烂鬼- | 671 | @深圳市血液中心 | 915 | @肇庆天气 |
| 184 | @丁香园 | 428 | @浪眼看深圳 | 672 | @深圳市应急管理办公室 | 916 | @折翼魔鬼520 |
| 185 | @东莞供电 | 429 | @乐纯的伙伴们 | 673 | @深圳市中国科学院仙湖植物园 | 917 | @这视频 |
| 186 | @东莞天气 | 430 | @乐乐宝贝2009 | 674 | @深圳市住房和建设局 | 918 | @浙江高速交警 |
| 187 | @东莞同城 | 431 | @了几次 | 675 | @深圳水务集团 | 919 | @浙江检察 |
| 188 | @冬天里的冻耳朵 | 432 | @梨视频 | 676 | @深圳特区报 | 920 | @浙视频 |
| 189 | @都市快报 | 433 | @梨重庆 | 677 | @深圳天气 | 921 | @郑州财经学院 |
| 190 | @斗门发布 | 434 | @李沧交警 | 678 | @深圳田雨 | 922 | @郑州工商学院 |
| 191 | @逗笼主锐sir | 435 | @李冬冬ah | 679 | @深圳同城 | 923 | @指旺财富 |
| 192 | @嘟嘟-菇娘 | 436 | @李连杰 | 680 | @深圳晚报 | 924 | @治安君 |
| 193 | @鹅组 | 437 | @李舒然 | 681 | @深圳网警 | 925 | @智者得爱 |
| 194 | @法治福田 | 438 | @李小咪-July | 682 | @深圳网警巡查执法 | 926 | @中车长春轨道客车股份有限公司 |
| 195 | @凡人普通用户 | 439 | @荔枝公寓 | 683 | @深圳微博发布厅 | 927 | @中国焙烤食品糖制品工业协会 |
| 196 | @樊鲜丽 | 440 | @荔直播 | 684 | @深圳西部高速交警 | 928 | @中国电力报 |
| 197 | @反恨国者联萌萌长 | 441 | @莲花派出所 | 685 | @深圳消防铁军 | 929 | @中国电力新闻网 |
| 198 | @防城港供电 | 442 | @恋随风来 | 686 | @深圳新安 | 930 | @中国东盟博览会 |
| 199 | @飞狼爱健身RockII | 443 | @辽宁交警 | 687 | @深圳新闻网 | 931 | @中国反邪教 |
| 200 | @肥肥啊飞 | 444 | @撩妹实力派阿猩 | 688 | @深圳刑侦局-深圳CID | 932 | @中国广核集团 |
| 201 | @匪猗不是仙女 | 445 | @廖琪七 | 689 | @深圳盐田交警 | 933 | @中国广州发布 |
| 202 | @封面新闻 | 446 | @灵儿的天空2010 | 690 | @深圳盐田警讯 | 934 | @中国警察网 |
| 203 | @佛山发布 | 447 | @凌雨PPT | 691 | @深圳义工联 | 935 | @中国南方航空 |
| 204 | @佛山供电 | 448 | @零度钢铁-yx | 692 | @深圳娱乐生活频道 | 936 | @中国气象局 |
| 205 | @佛山交警 | 449 | @零废弃联盟 | 693 | @深圳娱乐台 | 937 | @中国侨都-江门发布 |
| 206 | @佛山人社 | 450 | @领事之声 | 694 | @深圳渔夫2011 | 938 | @中国青年报 |
| 207 | @佛山天气 | 451 | @溜肉丁儿 | 695 | @深圳政府法制 | 939 | @中国人口福利基金会 |
| 208 | @佛山同城 | 452 | @浏阳公安 | 696 | @深圳之窗网 | 940 | @中国日报 |
| 209 | @福保派出所 | 453 | @柳州交警 | 697 | @深圳朱建平 | 941 | @中国社会福利基金会 |
| 210 | @福强派出所 | 454 | @六盘水供电 | 698 | @神大胖 | 942 | @中国天气 |
| 211 | @福田发布 | 455 | @龙岗发布 | 699 | @沈月 | 943 | @中国铁路 |
| 212 | @福田红树林生态公园 | 456 | @龙岗交警 | 700 | @生活如花花似梦 | 944 | @中国消防 |
| 213 | @福田华强北派出所 | 457 | @龙岗频道 | 701 | @生命时报 | 945 | @中国新闻网 |
| 214 | @福田警察 | 458 | @龙华的小伙伴 | 702 | @生态绿谷石岩 | 946 | @中国新闻周刊 |
| 215 | @福田派出所 | 459 | @龙华人 | 703 | @尸姐 | 947 | @中警安徽 |
| 216 | @福田区图书馆 | 460 | @陇上杨生 | 704 | @十堰市公安局东岳分局 | 948 | @中山发布 |
| 217 | @福田人 | 461 | @陆湘纯 | 705 | @时间视频 | 949 | @中山供电 |
| 218 | @傅警官漫画 | 462 | @路西法2816960175 | 706 | @世界的大姚的 | 950 | @中山同城 |
| 219 | @港铁深圳 | 463 | @罗湖城事 | 707 | @世界卫生组织 | 951 | @中新视频 |
| 220 | @高明公安 | 464 | @罗湖共青团 | 708 | @逝去的天空 | 952 | @中新网海南 |
| 221 | @高速交警嘉兴支队 | 465 | @罗雪儿在线 | 709 | @手户秦始皇 | 953 | @中央气象台 |
| 222 | @高速交警宁波支队 | 466 | @洛洛懒婆娘 | 710 | @手机用户1855854061 | 954 | @鐘小芳sell-fong |
| 223 | @高速交警绍兴支队 | 467 | @洛婉恩 | 711 | @首都机场 | 955 | @重庆晚报 |
| 224 | @戈蓝V | 468 | @骆嘉骏Jiajunlok | 712 | @首都网警 | 956 | @朱广权 |
| 225 | @各位老板 | 469 | @旅行走失的喵星人... | 713 | @书吧 | 957 | @珠海电视新闻 |
| 226 | @各位深圳家长们为确保孩子及时享受医保待遇 | 470 | @马鞍山交警 | 714 | @舒肤佳Safeguard | 958 | @珠海电台交通875 |
| 227 | @更北京 | 471 | @马大克先生 | 715 | @刷剧君 | 959 | @珠海电台先锋951 |
| 228 | @公安部打四黑除四害 | 472 | @蚂蚁金服 | 716 | @睡迟了对娃儿不好 | 960 | @珠海公安 |
| 229 | @公安部交通安全微发布 | 473 | @蚂蚁金服公益 | 717 | @顺顺儿的顺儿 | 961 | @珠海供电 |
| 230 | @公安主持人 | 474 | @罵乌鸦 | 718 | @四川达州消防 | 962 | @珠海交警 |
| 231 | @共青团中央 | 475 | @茂名供电 | 719 | @四川长安网 | 963 | @珠海天气 |
| 232 | @菇娘别惹恼我 | 476 | @茂名天气 | 720 | @苏s_s京 | 964 | @珠海新闻网官方微博 |
| 233 | @莞香花开 | 477 | @茂名网发布 | 721 | @苏炳添 | 965 | @株洲公安 |
| 234 | @光明发布 | 478 | @茂名网微博 | 722 | @苏州公安 | 966 | @猪娃娃520 |
| 235 | @光明家长 | 479 | @梅林派出所 | 723 | @所有驾驶员 | 967 | @竹语童话 |
| 236 | @光明建筑工务局 | 480 | @梅州供电 | 724 | @太原贴吧 | 968 | @专注于-小窝草YYY |
| 237 | @光明人 | 481 | @美联英语-立刻说-i... | 725 | @太原铁路 | 969 | @紫光阁 |
| 238 | @广东电网 | 482 | @美芝z | 726 | @体脂个位数再改名 | 970 | @宗小屁股是碎叫困难户 |
| 239 | @广东发布 | 483 | @魅影响 | 727 | @天安派出所 | 971 | @粽粽粽粽粽粽粽 |
| 240 | @广东交警 | 484 | @莔茵茵 | 728 | @天罡星2 | 972 | @醉卧枫林晚YL |
| 241 | @广东交通频道 | 485 | @梦的鱼最爱晴晴 | 729 | @天虹 | 973 | @遵义供电官微 |
| 242 | @广东教育 | 486 | @迷恋环岛路 | 730 | @天虹虹领巾 | 974 | @做什HD麼 |
| 243 | @广东民政 | 487 | @米歇尔任 | 731 | @天虹华南 |  |  |
| 244 | @广东省高级人民法院 | 488 | @秒拍 | 732 | @天津交警 |  |  |

**Twitter mention contacts identified from tweets posted by Hong Kong-based organizational actors**

| No | Mention contact | No | Mention contact | No | Mention contact | No | Mention contact | No | Mention contact | No | Mention contact |
| --- | --- | --- | --- | --- | --- | --- | --- | --- | --- | --- | --- |
| 1 | @500startups | 64 | @cghelenastorm | 127 | @fshongkong | 190 | @its_int_news | 253 | @piccone_tony | 316 | @techcrunch |
| 2 | @a_sinodinos | 65 | @chaunog | 128 | @ftahkofficial | 191 | @jamesdysonaward | 254 | @pierregagnaire | 317 | @technodeblog |
| 3 | @acciona | 66 | @chengxinpan | 129 | @ftserussell | 192 | @jameskondo | 255 | @pingan_group | 318 | @techstars |
| 4 | @accuitytweets | 67 | @chinabriefing | 130 | @garynobes | 193 | @jancisrobinson | 256 | @pingantech | 319 | @teph27 |
| 5 | @adamcampton | 68 | @chinawaterrisk | 131 | @gcunning12 | 194 | @jdphotography | 257 | @pizzaexpresshk | 320 | @thediacommunity |
| 6 | @adamposen | 69 | @circlepay | 132 | @geoparquegrutas | 195 | @jeffielam | 258 | @pnab_ | 321 | @thenamedicine |
| 7 | @aejmc | 70 | @cisco | 133 | @ggronvall | 196 | @jenbalisi | 259 | @pnasnews | 322 | @thepalass |
| 8 | @airwallex | 71 | @citibank | 134 | @globalgeoparks | 197 | @jennieorchard | 260 | @polyu_fb | 323 | @thestandardhk |
| 9 | @alexisbadel | 72 | @citymapper | 135 | @glorytam | 198 | @jerrychau15 | 261 | @polyushtm | 324 | @theworldunirank |
| 10 | @alifesjourneys | 73 | @citypossible | 136 | @goldmansachs | 199 | @jhsph_chs | 262 | @prenetics | 325 | @ticketflap |
| 11 | @aligstudios | 74 | @clavreulr | 137 | @gordonramsay | 200 | @jmschku | 263 | @pri_news | 326 | @time |
| 12 | @alipay | 75 | @climatecentral | 138 | @greenpeace | 201 | @johnwoodrtr | 264 | @pulitzerprizes | 327 | @timeouthk |
| 13 | @allianzgi_view | 76 | @clmannecon | 139 | @greenpeace_de | 202 | @karisteetan | 265 | @pwc_china | 328 | @timeshighered |
| 14 | @amcollegegastro | 77 | @cnbc | 140 | @greenpeaceeu | 203 | @kathymatsui | 266 | @pwma_hk | 329 | @tngfintech |
| 15 | @american_heart | 78 | @consensys | 141 | @gresb | 204 | @kpmgchina | 267 | @qantas | 330 | @tommentatornz |
| 16 | @amtdgroup | 79 | @corenetglobal | 142 | @gsma | 205 | @ladyandpups | 268 | @qftravelinsider | 331 | @tomorlik |
| 17 | @angustsui | 80 | @csapac | 143 | @guardianeco | 206 | @laligaen | 269 | @r_chl_ | 332 | @travelwithrandy |
| 18 | @anindya0909 | 81 | @csis | 144 | @guggenheim | 207 | @lch_clearing | 270 | @regulationasia | 333 | @tsinghua_uni |
| 19 | @anjalimittal | 82 | @cuhkmedicine | 145 | @haidilun | 208 | @lego_group | 271 | @repricklarsen | 334 | @tvbcom |
| 20 | @antoinehamelin | 83 | @cuhkofficial | 146 | @hamilton_hung | 209 | @lgim | 272 | @researchsea | 335 | @twfhk |
| 21 | @aprigf_ | 84 | @cxo_futureiot | 147 | @hammerseries | 210 | @lindsayparkrace | 273 | @rgaxglobal | 336 | @ubs |
| 22 | @arrowglobal | 85 | @cyberport_hk | 148 | @hansonrobotics | 211 | @linkreithk | 274 | @richardhoiles | 337 | @unep_fi |
| 23 | @asia_fruit | 86 | @dannyrrussel | 149 | @harvard | 212 | @lj__andrew | 275 | @riskdotnet | 338 | @unesco |
| 24 | @asia21leaders | 87 | @dannysmemo | 150 | @hashkeygroup | 213 | @longines | 276 | @rivieracentre | 339 | @unescoearth |
| 25 | @asiamoney | 88 | @daytaai | 151 | @helenziareal | 214 | @lunchactually | 277 | @robgmacfarlane | 340 | @unescouk |
| 26 | @asianbanking | 89 | @deakin | 152 | @hello_broadcast | 215 | @marketaxess | 278 | @rodplummer | 341 | @universitysa |
| 27 | @asiancha | 90 | @dealogic | 153 | @heraldlinde | 216 | @mastercard | 279 | @romygill_ | 342 | @upgradecredit |
| 28 | @asianpvtbanker | 91 | @dell | 154 | @hhksanderson | 217 | @mcfaul | 280 | @roomtoread | 343 | @urbansketchers |
| 29 | @asiapolicy | 92 | @desarthe | 155 | @hhkspeel | 218 | @megvii | 281 | @rosemontstud | 344 | @uruguay_natural |
| 30 | @asiasociety | 93 | @deveshsahai | 156 | @hinrichfdn | 219 | @mehvesh | 282 | @ruthshapirocaps | 345 | @usc |
| 31 | @asiasocietyaus | 94 | @dinoecosystems | 157 | @hkairlines | 220 | @messefrankfurt | 283 | @rwanderlands | 346 | @vectr_ventures |
| 32 | @asiasocietych | 95 | @djwhytetrainer | 158 | @hkairport | 221 | @metro_radio | 284 | @sanjoyroytwa | 347 | @victordzau |
| 33 | @asiasocietyhk | 96 | @donnieyenct | 159 | @hkartgal | 222 | @mh8s | 285 | @saskiaraodehaas | 348 | @viet_t_nguyen |
| 34 | @asiasocietyjp | 97 | @duncantang | 160 | @hkbaptistu | 223 | @microsoft | 286 | @schofieldchad | 349 | @vincenthocy |
| 35 | @asiasocietyny | 98 | @durham_uni | 161 | @hkejgroup | 224 | @mindhongkong | 287 | @schusterdeclan | 350 | @visa |
| 36 | @aws | 99 | @durhamchemistry | 162 | @hkexgroup | 225 | @mingtiandi | 288 | @scmp | 351 | @vmware |
| 37 | @bbcearth | 100 | @edward_sadler | 163 | @hkfyg | 226 | @mo_hkg | 289 | @scmpnews | 352 | @wef |
| 38 | @beautystablehk | 101 | @edxonline | 164 | @hkgeopark | 227 | @mo_hotels | 290 | @scotdevint | 353 | @wendyscutler |
| 39 | @behavioral_med | 102 | @eenewsupdates | 165 | @hkharbourcity | 228 | @morganstanley | 291 | @seannyboyng | 354 | @westernsydneyu |
| 40 | @bernarvenet | 103 | @egnetwork | 166 | @hkia | 229 | @mplusmuseum | 292 | @sequoia | 355 | @wework |
| 41 | @bioworld | 104 | @emilycnbc | 167 | @hkjc_racing | 230 | @mr_dr3w | 293 | @seraitrade | 356 | @wolters_kluwer |
| 42 | @blake_shinn | 105 | @englishriviera | 168 | @hkmagovhk | 231 | @mrkrudd | 294 | @sfi_hk | 357 | @womencorpdirs |
| 43 | @bloombergau | 106 | @epassoc | 169 | @hkpolyudesign | 232 | @msci_inc | 295 | @shashitharoor | 358 | @world_wildlife |
| 44 | @bloombergenv | 107 | @equidia | 170 | @hkpolyueie | 233 | @mtrupdate | 296 | @shubhendrarao | 359 | @wriclimate |
| 45 | @bmedlab | 108 | @euromoney | 171 | @hktakavic | 234 | @muhyiddinyassin | 297 | @sibos | 360 | @wrienergy |
| 46 | @bmw | 109 | @fashionunited | 172 | @hku_science | 235 | @mukulikab | 298 | @siemens | 361 | @wrifinance |
| 47 | @bnpparibasapac | 110 | @fclor_racing | 173 | @hkuniversity | 236 | @nab | 299 | @smartone_hk | 362 | @wwfhk |
| 48 | @bofa_business | 111 | @financialtimes | 174 | @hkuonline | 237 | @nanieshaziani | 300 | @socgen_hk | 363 | @wwfjapan |
| 49 | @bonnieglaser | 112 | @finnovasia | 175 | @hkust | 238 | @naomiosaka | 301 | @societegenerale | 364 | @xiaomi |
| 50 | @brand_hk | 113 | @finquiry | 176 | @hongkongctu | 239 | @natgallerysg | 302 | @sofi | 365 | @yahoofinance |
| 51 | @btasiapacific | 114 | @fitchratings | 177 | @hongkongfintech | 240 | @naturebiotech | 303 | @spencerstuart | 366 | @yingworld |
| 52 | @burrengeopark | 115 | @flexport | 178 | @hongkongpen | 241 | @neilcallan78 | 304 | @spotify | 367 | @yourstoryco |
| 53 | @businesstimes | 116 | @foodiehk | 179 | @hooverinst | 242 | @newsgovhk | 305 | @stanford | 368 | @youtube |
| 54 | @campaignasia | 117 | @foodlinkhk | 180 | @hsbc | 243 | @nexus_us | 306 | @stanfordbosp | 369 | @zestmoney |
| 55 | @campiglioapt | 118 | @forbes | 181 | @hsh_hongkong | 244 | @ngongping360 | 307 | @stcom | 370 | @zirosou |
| 56 | @caps_asia | 119 | @foreignaffairs | 182 | @iammarkelwin | 245 | @novaeureka | 308 | @stestock | 371 | @zpurton |
| 57 | @cataliize | 120 | @foundersfund | 183 | @idharker | 246 | @observingjapan | 309 | @stevem_moran | 372 | @zubinfoundation |
| 58 | @cavaleiro83 | 121 | @fourseasons | 184 | @insuretechnow | 247 | @officialjgn | 310 | @streets_julia | 373 | @zuibrahim |
| 59 | @cbrecapitalmkts | 122 | @fowgroup | 185 | @investhk | 248 | @omannewsagency | 311 | @svp_vertpaleo |  |  |
| 60 | @cbreresearch | 123 | @francegalop | 186 | @iproducetv | 249 | @orientationsmag | 312 | @taiwannews886 |  |  |
| 61 | @cbreretail | 124 | @fraserinstitute | 187 | @iqiyi | 250 | @ourcrowd | 313 | @tate |  |  |
| 62 | @cbreworkplace | 125 | @fredericneumann | 188 | @irenenychu | 251 | @outleadership | 314 | @tatlerasia |  |  |
| 63 | @cfainstitute | 126 | @fsdchk | 189 | @isdaconferences | 252 | @patniewoo | 315 | @teamworkarts |  |  |
